# Designing Cooperative Molecular Glues for FKBP12-MAPRE1 with Enhanced-Sampling Molecular Dynamics Simulations

**DOI:** 10.1021/jacs.6c12127

**Published:** 2026-10-01

**Authors:** Shu-Yu Chen, Riccardo Solazzo, Marianne Fouché, Hans-Jörg Roth, Birger Dittrich, Sereina Riniker

**Affiliations:** † Department of Chemistry and Applied Biosciences, 27219 ETH Zurich, Vladimir-Prelog-Weg 2, Zurich 8093, Switzerland; ‡ Novartis Biomedical Research, 111826 Novartis Campus, Basel 4002, Switzerland

## Abstract

Molecular glues (MGs) offer a unique modality for inducing protein–protein interactions (PPIs), yet their rational design is often hindered by the limitations of static structural data. Here, we present a computational pipeline for the design of cooperative MGs for the FKBP12-MAPRE1 system using replica exchange with solute scaling (REST2) for enhanced sampling in molecular dynamics (MD) simulations. By sampling the conformational ensembles of binary and ternary complexes, we identified a “stabilized configurational space” that facilitates ternary-complex formation through a combination of conformational selection and induced-fit mechanisms. Based on the simulations, we developed a model that correlates PPI induction information from the simulations with the experimental cooperativity. This model was subsequently used to select and rank a small library of compounds prospectively, of which five were selected for synthesis and testing. Three of the five new compounds exhibited apparent cooperativity equal to or greater than that of the best previously known variants. This study demonstrates that targeting shared configurations within dynamic ensembles is a viable strategy for the rational optimization of cooperative molecular glues.

## Graphical abstract

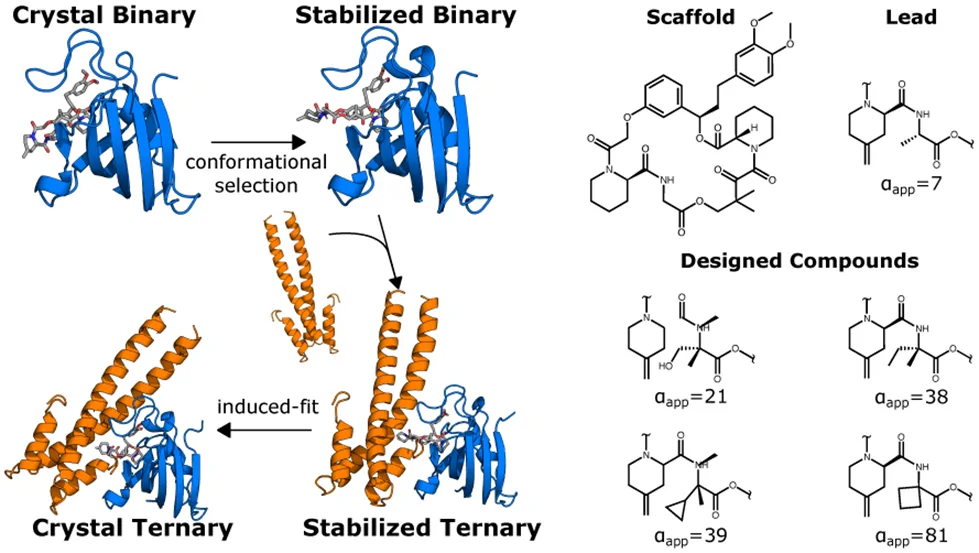

## Introduction

Molecular glues (MGs) have emerged as a powerful modality in drug discovery, offering the unique ability to induce novel protein–protein interactions (PPIs) and target flat protein surfaces that have been previously considered inaccessible.^1–4^ By stabilizing the interface between an E3 ligase (or another presenter protein) and a target protein, molecular glues can trigger downstream effects such as ubiquitination and degradation.^5,6^ Compared to bifunctional PPI inducers such as proteolysis targeting chimeras (PROTACs), MGs often display higher cooperativity and are less susceptible to the hook effect (or prozone effect).^7–9^ Cooperativity in induced-proximity systems has been described within the thermodynamic framework of three-body binding equilibria,^10^ while structural studies of PROTAC ternary complexes have demonstrated how favorable protein–protein interactions can drive cooperative ternary-complex formation.^11^ However, despite their potential, rational design and even optimization of molecular glues remain a significant challenge. While numerous computational approaches, including physics-based and machine learning methods, have been developed in recent years to facilitate ternary-complex prediction, these tools are still far from fully capturing the dynamics and determinants of cooperativity that govern ternary-complex formation (see the review in ref 12 and references therein). Consequently, discovery often continues to rely on serendipity or extensive empirical screening.^13–19^

An example of this challenge is the system involving FKBP12 (FK506-binding protein 12) and MAPRE1 (microtubule associated protein RP/EB family member 1). In the recent work by Salcius et al.,^20^ a macrocyclic MG R,S-SLF-1a (**1a**) was discovered to induce interactions between FKBP12 and MAPRE1 (Figure 1A). In the same work, the structure–activity relationship of the MAPRE1-recognition motif was studied by mutating two sites: the 4-piperidine moiety (Figure 1A–B, circled in yellow) and the α-position (Figure 1A–B, circled in red). Several modifications at the α-position of this site retain glue activity, but with a wide range of absolute cooperativity as determined by follow-up work^21^ (Figure 1C and Table 1). In contrast, modifications to the 4-piperidine moiety consistently impede MAPRE1 recruitment^20^ (Figure 1D). The static picture from the crystal structure could not explain how these subtle changes influence cooperativity and glue activity.

**Figure 1:**
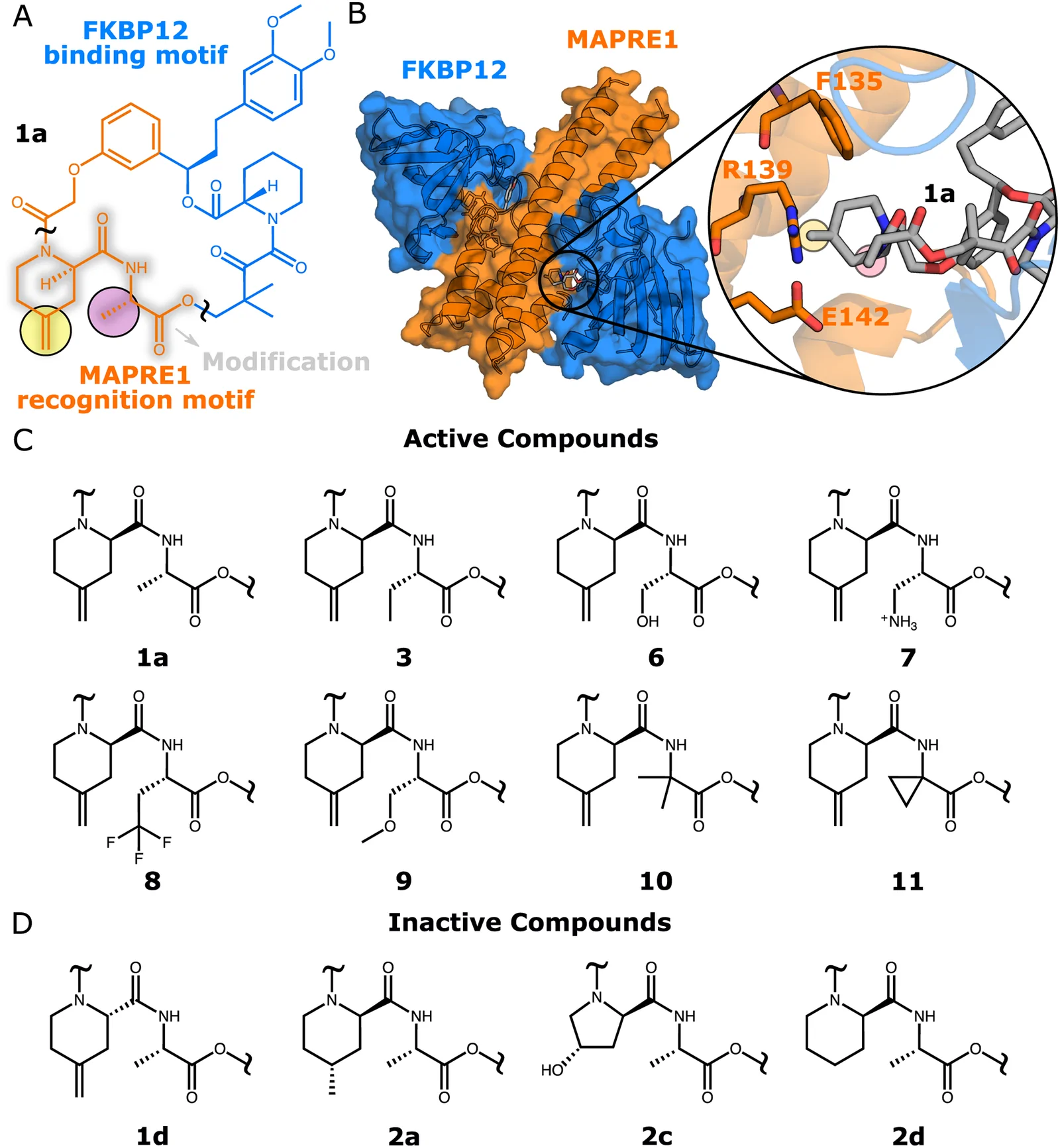
FKBP12-MG-MAPRE1 ternary complex. (A): 2D structure of MG **1a**, highlighting the FKBP12-binding motif (blue) and the MAPRE1-recognition motif (orange). (B): Crystal structure of the ternary complex (PDB ID: 9CO5 ^20^) with FKBP12 (blue), MG **1a** (gray), and MAPRE1 (orange). The zoom-in view highlights the MAPRE1 residues surrounding the 4-piperidine moiety (highlighted in yellow) and the α-position (highlighted in red) of **1a**. The methylene group of the 4-piperidine ring forms close contact with L138, R139, and E142 of MAPRE1. The methyl group at the α-position is exposed to the solvent and barely contacts the proteins. (C): 2D structures of selected active compounds from ref 20. (D): 2D structures of selected inactive compounds. The experimental data of compounds **1d** and **2a** are reported in ref 20, whereas the data of compounds **2c** and **2d** are shown in Section S1.1 of the Supporting Information.

**Table 1:** Absolute and Apparent Cooperativity of the Active Compounds Investigated in This Study (Figure 1C)^a^

Compound	1a	3	6	7	8	9^b^	10	11
α_*abs*_	1,951	2,200	4,484	237	1,509	–	5,405	10,405
$\alpha_{app}^{MAPRE1}$	1,942	2,177	4,386	232	1,490	232	5,305	10,000

a Data for all compounds except **9** were retrieved directly from ref 21.

b Because of the low quality of the FKBP12-binding data for compound **9**, apparent cooperativity from the MAPRE1-binding assays was used to approximate the absolute cooperativity for the fitting in the study. Experimental data on **9** are presented in Section S1.2 in the Supporting Information.

In this work, we bridge the gap between the static structural data and observed cooperativity by investigating the dynamics of binary and ternary complexes using enhanced-sampling molecular dynamics (MD) simulations. By extensively sampling the ensembles of the FKBP12-ligand complexes with and without MAPRE1 using replica exchange with solute scaling (REST2),^22^ we formulated the hypothesis that high cooperativity arises from the PPI in the specific “stabilized configurational space” that is shared in both ensembles. Based on the recently developed thermodynamic foundations of cooperativity,^23^ we established a computational pipeline to predict the cooperativity of new MG candidates. First, we proposed a model that correlates the experimental cooperativity and the computed PPI matrices by developing and evaluating the model using the previously reported variants of **1a**. Subsequently, we applied the model to prospectively design novel variants of **1a** and predict their cooperativity *in silico*. Five candidates were synthesized and experimentally tested (together with resynthesis and retesting of five previous compounds). Three of the novel compounds exhibit an apparent cooperativity similar to or higher than the previously known most cooperative retested compound **11**. Another one shows an apparent cooperativity similar to the second-most cooperative retested compound **6**. Our results demonstrate that incorporation of conformational dynamics and induced PPI analysis is a viable strategy for the rational design and optimization of cooperative MGs.

## Results and Discussion

### Modification of the 4-Piperidine Moiety Destabilizes the Ternary Complex

To investigate the difference between the inactive compounds and the active ones, four active compounds covering a wide range of apparent cooperativity (**1a**, **8**, **10**, and **11**, see Figure 1C) and four inactive compounds (**1d**, **2a**, **2c**, and **2d**, see Figure 1D) were studied in the ternary complex (FKBP12-ligand-MAPRE1 = FLM) with MD simulations for 1 μs (three replicates). In addition, a ligand-free (apo) system was also simulated for comparison. During the simulations, a dynamic interface between the two proteins was observed, which sees the fluctuating formation and disappearance of “native” and “nonnative” contacts between FKBP12 and MAPRE1, differing mainly by the tilt motion of MAPRE1 (Figure 2A–B). “Native” contacts are PPIs observed in the available crystal structure of the ternary complex, whereas PPIs only seen in the simulations are considered “nonnative”. While the apo system quickly lost most of the native contacts (as expected due to the very low affinity of FKBP12 to MAPRE1 in the absence of glues^20^), no significant differences in terms of PPI contacts were observed between inactive and active compounds within the simulation time scale (Figure 2C–D).

**Figure 2:**
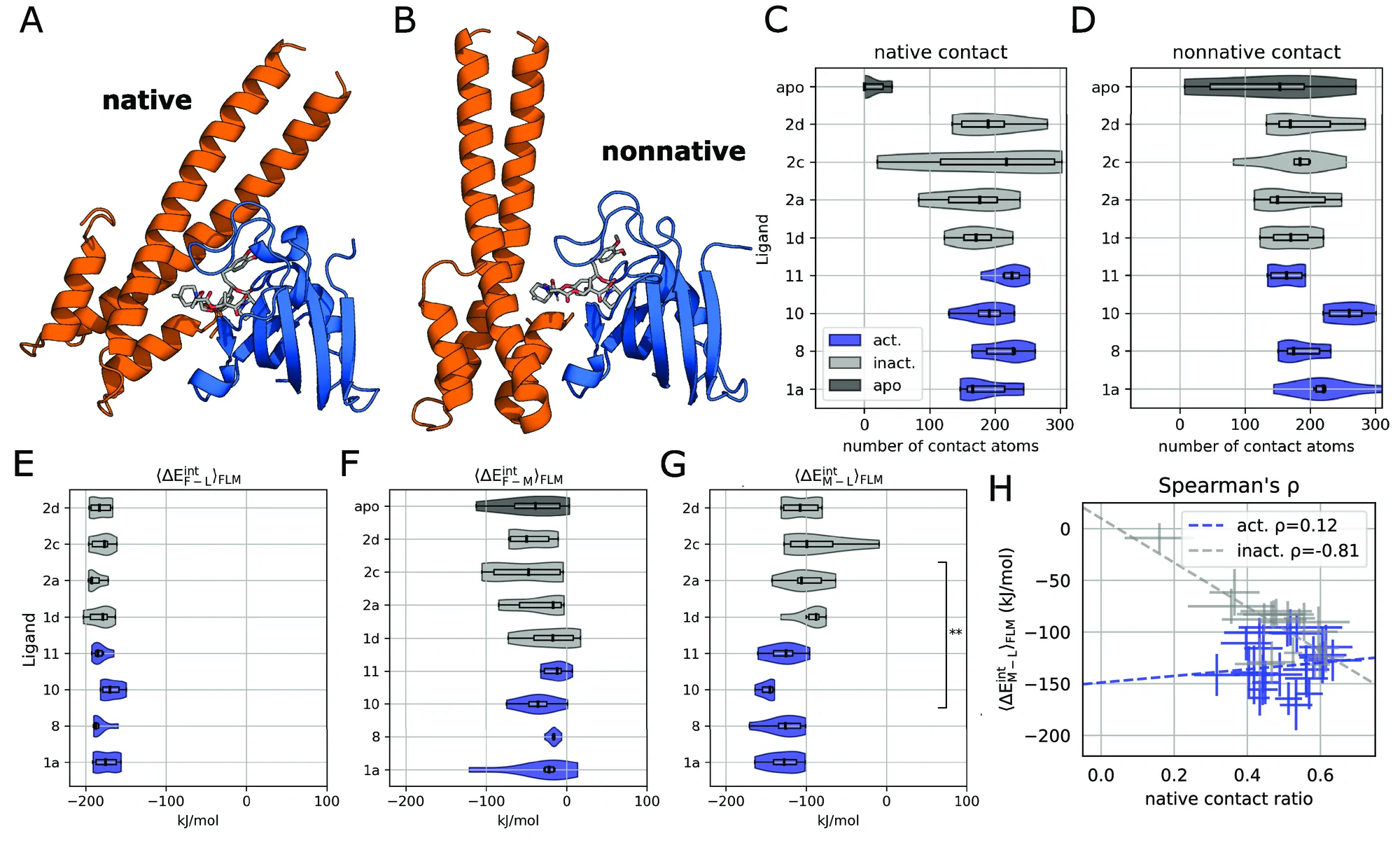
MD simulations of FKBP12-MG-MAPRE1 ternary complex (three replicates of 1 μs). Only the last 800 ns of each trajectory was used for the contact and energy evaluation. (A): Crystal structure with “native” contacts between FKBP12 (blue) and MAPRE1 (orange). (B): MD snapshot showing “nonnative” contacts between the protein partners. (C): Distribution of the number of native contacts between FKBP12 and MAPRE1 for apo (black), active compounds (blue), and inactive compounds (gray). (D): Distribution of the number of nonnative contacts between the proteins. (E): Interaction energy ${\left\langle \Delta E_{F-L}^{int}\right\rangle }_{FLM}$ between FKBP12 and the ligands in the ternary complex (*p*_comp_ = 0.1919, *p*_int_ = 0.1112). (F): Interaction energy ${\left\langle \Delta E_{F-M}^{int}\right\rangle }_{FLM}$ between FKBP12 and MAPRE1 in the ternary complex (*p*_comp_ = 0.2069, *p*_int_ = 0.1928). (G): Interaction energy ${\left\langle \Delta E_{M-L}^{int}\right\rangle }_{FLM}$ between MAPRE1 and the ligands in the ternary complex (*p*_comp_ = 1.881 × 10^–3^, *p*_int_ = 2.126 × 10^–5^). Note that *p*_comp_ denotes the primary compound-level Welch’s *t*-test (*n* = 4 compounds per activity group), and *p*_int_ denotes the exploratory interface-level Welch’s *t*-test (*n* = 24 interface observations per activity group). Details of the statistical analysis are provided in the Methods section. Violin plots in (C–G) show the statistics of three simulations with two interfaces in each simulation (*N* = 6). The inset box plot shows the median of the distribution ± 1.5 × interquartile range. (H): Correlation of ${\left\langle \Delta E_{M-L}^{int}\right\rangle }_{FLM}$ and the native contact ratio for active (blue) and inactive compounds (gray). The linear fits are shown as dashed lines. Each data point represents the mean and standard deviation of each interface (*N* = 48). Time series of the data shown in (C–G) are provided in Section S2 in the Supporting Information.

Next, we analyzed the pairwise interaction energies in the FLM ensembles with MM/GBSA.^24,25^ Note that the interaction energies measured here only account for the interactions in the ternary ⟨⟩_*FLM*_ state and do not take into account the entropic effects before the ternary complexation. All eight compounds exhibit similar interaction energies with FKBP12, ${\left\langle \Delta E_{F-L}^{int}\right\rangle }_{FLM}$ (Figure 2E), which agrees with the experimental findings that the modifications of **1a** in the 4-piperidine moiety do not alter its affinity for FKBP12.^20,21^ Similarly, the interaction energy between the protein partners, ${\left\langle {\Delta E}_{F-M}^{int}\right\rangle }_{FLM}$ , is largely unaffected by the type of ligand binding (Figure 2F). On the other hand, the MAPRE1-ligand interaction energy, ${\left\langle \Delta E_{M-L}^{int}\right\rangle }_{FLM}$ , shows statistical significance between active and inactive compounds (*p* = 1.881 × 10^–3^ at the compound level and *p* = 2.126 × 10^–5^ at the interface level, Figure 2G). This suggests that inactive compounds cannot induce the ternary complex due to the dual-binding criterion^26^ (i.e., a molecular stabilizer needs to possess some affinity for both proteins). Interestingly, while in most simulations of the active compounds more than 40% of the contacts between the two proteins were native (i.e., native contact ratio >0.4), the inactive compounds show an anticorrelation between the MAPRE1-ligand interaction strength and the native contact ratio (Spearman’s correlation coefficient ρ = −0.81 for inactive compounds, Figure 2H), which is consistent with the hypothesis that firm interaction between the ligand and MAPRE1 stabilizes ternary complex configurations that contain many native contacts. Further analysis of the time series of these features and the RMSD of each component is shown in Section S2 in the Supporting Information.

### Common Ligand Conformation between the Binary and Ternary Complexes

In contrast to other macrocyclic MGs such as cyclosporine A, FK506, and rapamycin, compound **1a** in the FKBP12-MAPRE1 system shows a significant conformational discrepancy between the crystal structures of the binary and ternary complexes^23^ (Figure 3A–B). Superposition of the crystal structures of FL (PDB ID: 9DCW^20^) and FLM (PDB ID: 9CO5 ^20^) shows that FKBP12 and the FKBP12-binding motif of **1a** (Figure 3A) remain rigid upon recruitment of MAPRE1 (TM score:^27^ 0.98). However, the MAPRE1-recognition motif of **1a** adopts an alternative conformation in the ternary complex to avoid a steric clash with MAPRE1 (Figure 3A). To characterize the conformational change of the MAPRE1-recognition motif between the binary and ternary complexes, we define the intermotif angle θ_*lig*_ by computing the angle formed by the planes fitted on the two motifs (Figure 3B). This angle θ_*lig*_ corresponds to −35.06° in the FL structure and 32.63° in the FLM structure. The difference in the ligand configuration implies the necessity of a conformational selection and/or induced-fit mechanism during the FL to FLM formation process, which is not immediately clear from the static structures. A detailed description of the protocol to calculate θ_*lig*_ is provided in Section S3 in the Supporting Information.

**Figure 3:**
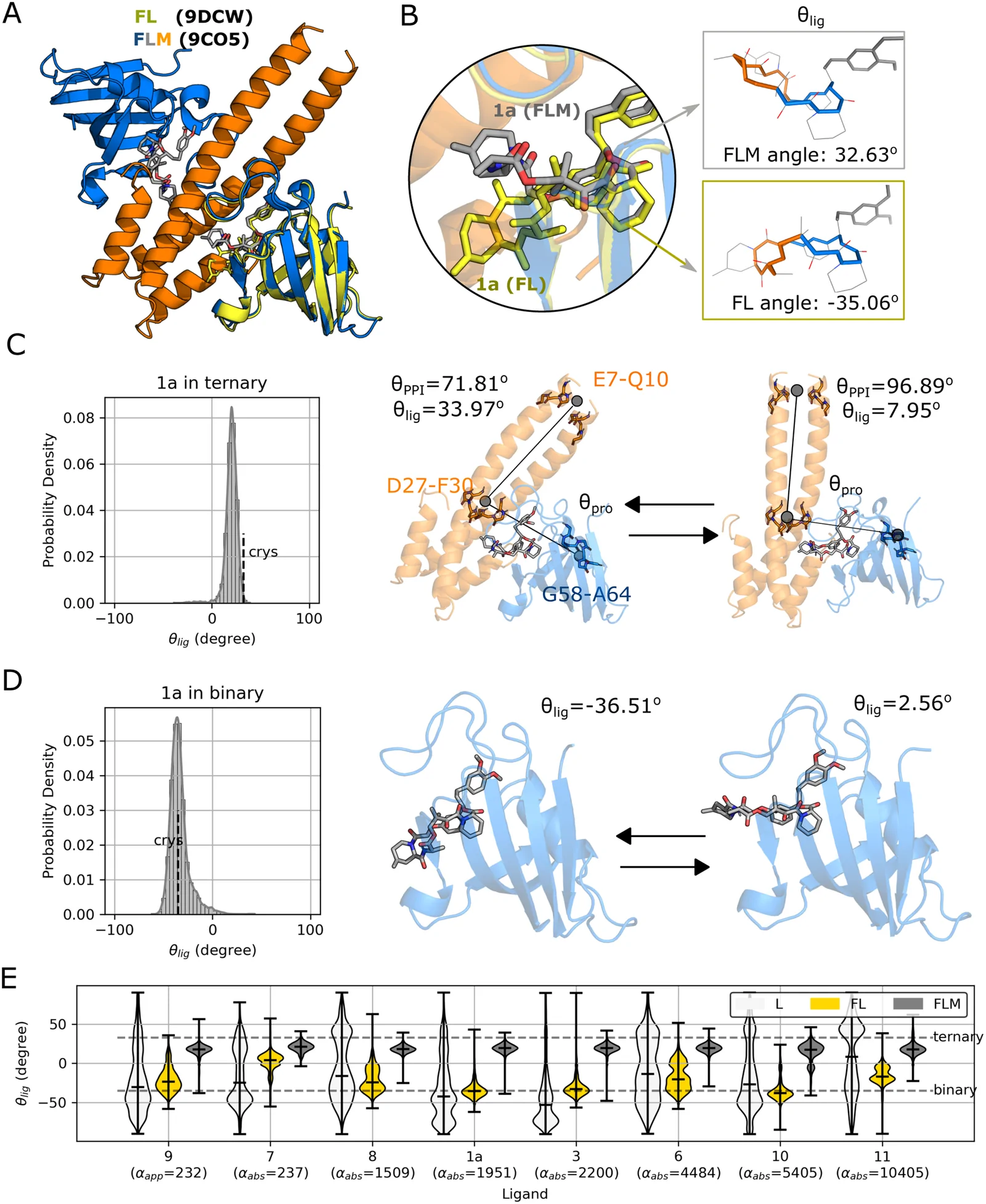
MAPRE1-induced conformational change of the ligand and the intermediate ligand conformation revealed in the REST2 ^22^ simulations. (A): Overlap of the binary (yellow) and ternary (blue, orange, gray) crystal structures with **1a** by aligning FKBP12. (B): Zoom-in view of the aligned crystal structures and the corresponding θ*_lig_* values. (C): Distribution of θ*_lig_* of **1a** in the ternary FLM simulations (left) and core-to-core angle of the ligand θ*_lig_* and the PPI tilting angle θ*_PPI_* characterizing the motion of the ternary complex (right). θ*_PPI_* measures the angle between the center of masses of E7-Q10 of MAPRE1, D27-F30 of MAPRE1, and G58-A64 of FKBP12. (D): Distribution of θ*_lig_* of **1a** in the binary FL simulations (left) and exemplary snapshots (right). (E): Distribution of θ*_lig_* of the ligands in the simulations of the ligand-only (white), binary FL (yellow), and ternary FLM (gray) systems. The ligands are shown in the order of increasing absolute cooperativity α*_abs_*. The two horizontal dashed lines indicate the θ*_lig_* observed in the binary and ternary crystal structures.

When monitoring θ_*lig*_ in the conventional MD simulations of the binary (FL) and ternary (FLM) complexes, we noted differences between the three replicates, which suggested convergence issues (Section S4 of the Supporting Information). Thus, we performed enhanced-sampling MD simulations using REST2 ^22^; only the REST2 results are shown in the following. In the FL and FLM simulations, θ_*lig*_ peaked around the values observed in the crystal conformations (Figure 3C–D). Notably, the simulations occasionally sampled a conformation with θ_*lig*_ around zero in both FL and FLM ensembles (Figure 3C–D), which we termed the intermediate ligand conformation. In the FLM complexes, this intermediate conformation coincides with the bending motion of MAPRE1, characterized by a large angle θ_*PPI*_ between three helical domains shown in Figure 3C. As a similar intermediate ligand conformation is occasionally sampled in the FL ensemble, we hypothesize that these low-probability intermediate conformations in both ensembles could explain a MAPRE1 recruitment pathway, as discussed in the next section.

To determine whether the intermediate conformations can also be adopted by other active compounds, we studied seven active compounds reported in ref 20: **3**, **6**, **7**, **8**, **9**, **10**, and **11**. In addition to the FL and FLM complexes, REST2 simulations were performed for the ligands alone in the aqueous solution. Figure 3E shows that all compounds can adopt a range of conformations with θ_*lig*_ between ±80^◦^ when free in solution. When bound to FKBP12 (FL ensemble), only conformations with θ_*lig*_ values between negative and around zero are accessible, which indicates that the binding pocket restricts the flexibility of the macrocycles. Intriguingly, conformations with negative θ_*lig*_ values are no longer reachable when MAPRE1 binds to the FL binary complex to form the ternary complex. Instead, zero to positive θ_*lig*_ are found for the ligands in the FLM ensemble. The fact that binding of MAPRE1 induces a stable ligand conformation that is rarely observed in the FL ensemble highlights the cooperative interplay between the ligand conformation, the ligand-MAPRE1 interaction, and native PPI contacts, which we shall discuss in the next section.

### Induced PPI in the Stabilized Configurational Space

The observation of a distinct yet overlapping conformational space in Figure 3E suggests that the formation of the ternary complex may be facilitated by both conformational selection and the induced-fit mechanism. This means that there is a subregion of the configurational space (ensemble) of the binary FKBP12-ligand complex that is suited for recruitment of MAPRE1. Similarly, there is a subregion of the configurational space of the ternary complex where the conformations of FKBP12 and the ligand overlap with conformations observed in the binary complex. In the following, we call these subregions of the binary and ternary ensembles the *stabilized configurational spaces*, which we denote with the superscript *s*. The superscript * is used to denote the minimal-energy configuration revealed by crystallography. Based on our observations from the MD simulations, we can formulate a hypothesis for the binding pathway, which is depicted in Figure 4A.

**Figure 4:**
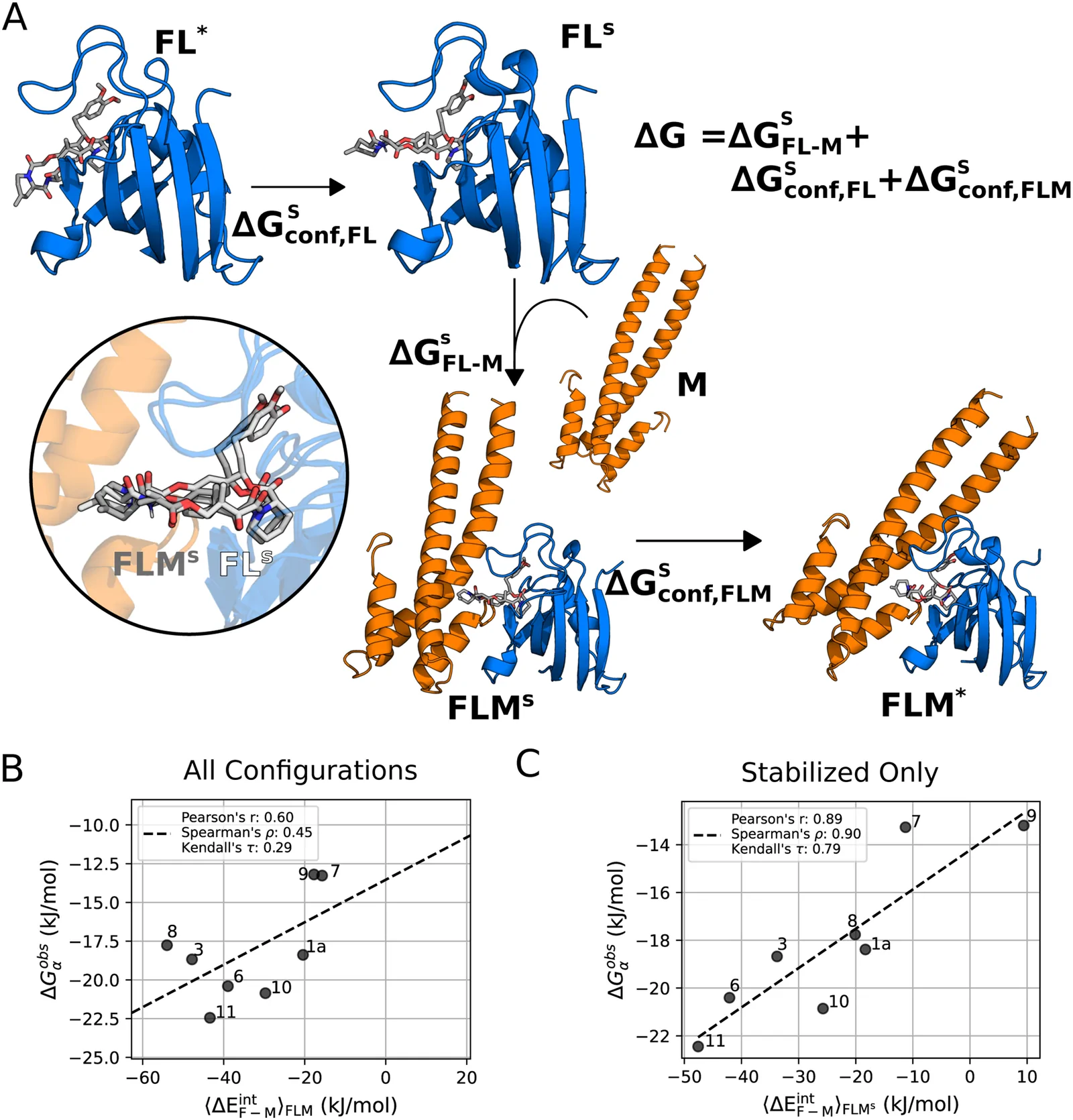
(A): Hypothesis of the FKBP12-ligand-MAPRE1 recruitment process. MAPRE1 (M) is shown in orange, ligand (L) in gray, and FKBP12 (F) in blue. The star superscript in FL* and FLM* indicates the minimal-energy configuration in the ensemble and the *s* superscript denotes the stabilized configurational space. (B–C): Correlation between the experimentally observed cooperativity and the computed PPIs between FKBP12 and MAPRE1 using all configurations (B) or using only the stabilized configurational space in the ternary ensemble (C).

First, the ligand in the FL complex could transition from the low-energy configuration (i.e., θ_*lig*_ ≤ −10°, FL*) to the stabilized configuration FL^*s*^ (where −10° ≤ θ_*lig*_ ≤ 10°). Second, MAPRE1 is recruited by weak ligand-MAPRE1 interactions and nonnative PPI contacts (this step can be viewed as conformational selection). Once the FLM complex is formed in the stabilized configurational space (FLM^*s*^, where −10° ≤ θ_*lig*_ ≤ 10°), the ligand undergoes another conformational change in the third step to strongly bind MAPRE1 and to induce native PPI contacts (FLM*, where θ_*lig*_ ≥ 10°). The common ligand conformation shared in the FL^*s*^ and FLM^*s*^ ensembles is highlighted in the inset box of Figure 4A. Note that the putative pathway presented here is derived from observations from the equilibrium simulations and does not exclude alternative pathways of ternary-complex formation.

According to the thermodynamics of cooperativity,^23^ the precise identification of such stabilized configurational space in the FLM ensemble provides us with an opportunity to compute (and subsequently predict, see below) the cooperativity of the system. If we do not consider this stabilized configurational space and naively compare the FKBP12-MAPRE1 interaction energy calculated over the full ensemble $\left({\left\langle {\Delta E}_{F-M}^{int}\right\rangle }_{FLM}\right)$ with the experimentally observed cooperativity, the correlation is not very good (Spearman coefficient ρ = 0.45, Figure 4B). The correlation is significantly improved (ρ = 0.90, Figure 4C) when considering only frames of the stabilized configurational space to compute the interaction energy between the two proteins $\left({\left\langle {\Delta E}_{F-M}^{int}\right\rangle }_{FLM^{s}}\right)$ . To determine this stabilized configurational space in practice, we aligned the FKBP12 structures frame-to-frame to compute whether a configuration of FL observed in the ternary FLM ensemble has a nonzero probability to occur in the binary FL ensemble (see Figure S41 in Section S5 in the Supporting Information for a schematic depiction of the procedure). Section S5 also contains more details about the frame-to-frame alignment procedure. Note that as the model was developed using only eight previously reported compounds, the retrospective correlation should be interpreted as model development rather than independent validation. The prospective compounds were selected before experimental testing, and their experimental results were not used to refit the model.

### Prospective Design of Cooperative Glues

Encouraged by the high correlation between the experimental cooperativity and ${\left\langle {\Delta E}_{F-M}^{int}\right\rangle }_{FLM^{s}}$ , our objective was to look for derivatives of **1a** that show similar or higher cooperativity. We noticed that the two most cooperative compounds **10** and **11** (Table 1) have two geminal substituents at the α-position in the MAPRE1-recognition motif (red circle in Figure 1A). Furthermore, the substituent(s) at the α-position of four of the five most cooperative compounds (**11**, **10**, **3**, and **1a**) are lipophilic. Based on these considerations, we screened commercial catalogues for amino-acid building blocks with two geminal substituents where the *D*-substituent is a methyl group (or part of a ring) and the *L*-substituent is lipophilic. As compound **6**, which showed the third highest cooperativity of the full set has a hydroxymethyl substituent at the α-position, we also considered some polar *L*-substituents. This produced 17 candidates, from which 12 were retained for prospective simulations based on their size and compatibility with the molecular-glue scaffold (**p0-pB**, Figure 5A). We used the same procedure as described in the previous section and the model fitted from the parent compounds to estimate their cooperativity (Figure 5B). From this, compounds **p0**, **p2**, **p3**, **p4**, and **p6** were predicted as the most cooperative compounds and were submitted for synthesis and experimental validation. As a control, the lead compound (**1a**), a moderately cooperative compound (**6**), a weakly cooperative compound (**9**), the most cooperative compound (**11**), and an inactive compound (**1d**) from the literature^20^ were also resynthesized and tested in the same assays.

**Figure 5:**
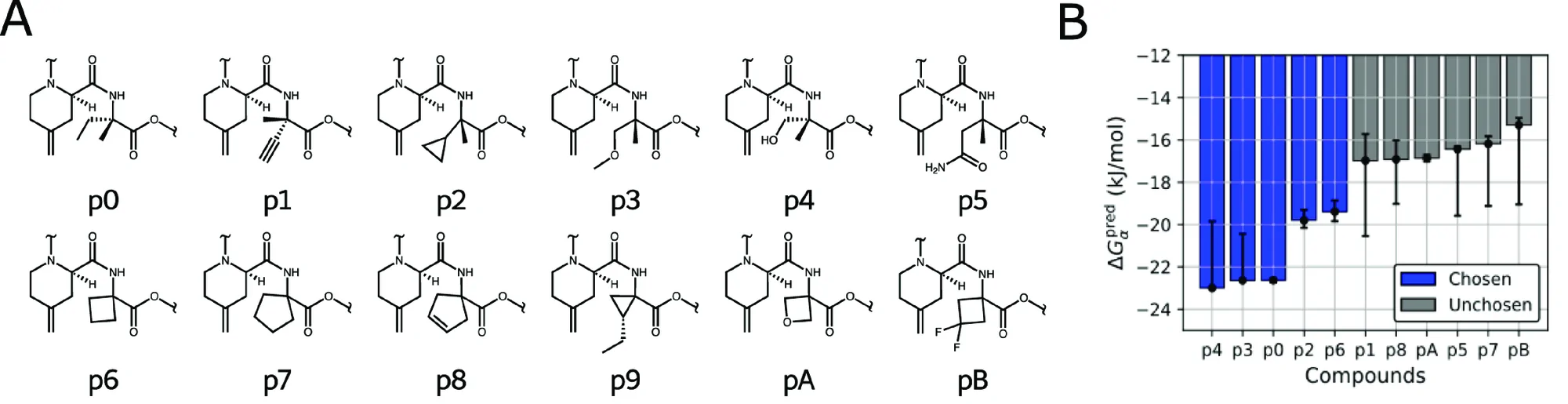
Prospective design of cooperative MGs for FKBP12-MAPRE1. (A): 12 derivatives of **1a** with two geminal substituents at the α-position. (B): Estimated cooperativity using the fitted model from the previously known compound set (see Figure 4C). The error bars show the 95% confidence interval using bootstrapping (*B* = 2000). The compounds chosen for experimental testing are colored in blue while the rest are colored in gray. The distributions of θ*_lig_* in the binary and ternary complexes are shown in Figure S43 in the Supporting Information.

### Experimental Testing of the Prospective Compounds

Absolute cooperativity α can be determined experimentally by either measuring *K*_*F‑L*_ and *K*_*F‑LM*_ or by measuring *K*_*M‑L*_ and *K*_*FL‑M*_ (Figure 6A). However, due to the limits of the experimental assay, no exact binary binding affinity between the ligands and MAPRE1, *K*_*M‑L*_, could be determined $\left(EC50_{binary}^{MAPRE1}>500\mu M\right)$ and thus no absolute cooperativity could be obtained. The MAPRE1-binding titration experiment is discussed in Section S6.2 in the Supporting Information. Instead, we calculate the apparent cooperativity as the ratio of the binary and ternary EC50 values $\left(\alpha_{app}^{FKBP12}=EC50_{binary}^{FKBP12}/EC50_{ternary}^{FKBP12}\right)$ by titrating the corresponding ligand to 40 nM labeled FKBP12 with or without the presence of 10 μM MAPRE1. For example, an apparent cooperativity $\alpha_{app}^{FKBP12}=7$ is observed for compound **1a**, indicated by the shift in EC50 values in the presence of 10 μM MAPRE1 (Figure 6B). The ranking of the $\alpha_{app}^{FKBP12}$ values of the known compounds agrees overall well with the absolute cooperativity α_*abs*_ reported in ref 21 (Figure 6C). Figure 6D summarizes $\alpha_{app}^{FKBP12}$ for each compound computed from the FKBP12-binding assay. In line with previous findings that compound **1d**, an epimer of **1a**, binds strongly to FKBP12 but cannot recruit MAPRE1, we find weak or no cooperativity $\left(\alpha_{app}^{FKBP12}<1\right)$ for **1d** in the new experiments. The active compounds **11**, **9**, and **6** exhibit apparent cooperativity ranging from 8 to 41. From the five prospective compounds, only **p3** shows little cooperativity $(\alpha_{app}^{FKBP12}=2.2\;\pm \;0.4$ , Figure 6D). Three compounds yielded apparent cooperativity similar to or greater than that of the best parent compound **11** $\left(\alpha_{\mathrm{app}}^{\mathrm{FKBP}12}=41\pm 13\right)$ : **p0** (38 ± 11), **p2** (39 ± 9), and **p6** (81 ± 44). Compound **p4** (21 ± 7) showed apparent cooperativity similar to that of the second-best retested parent compound **6** (24 ± 7).

**Figure 6:**
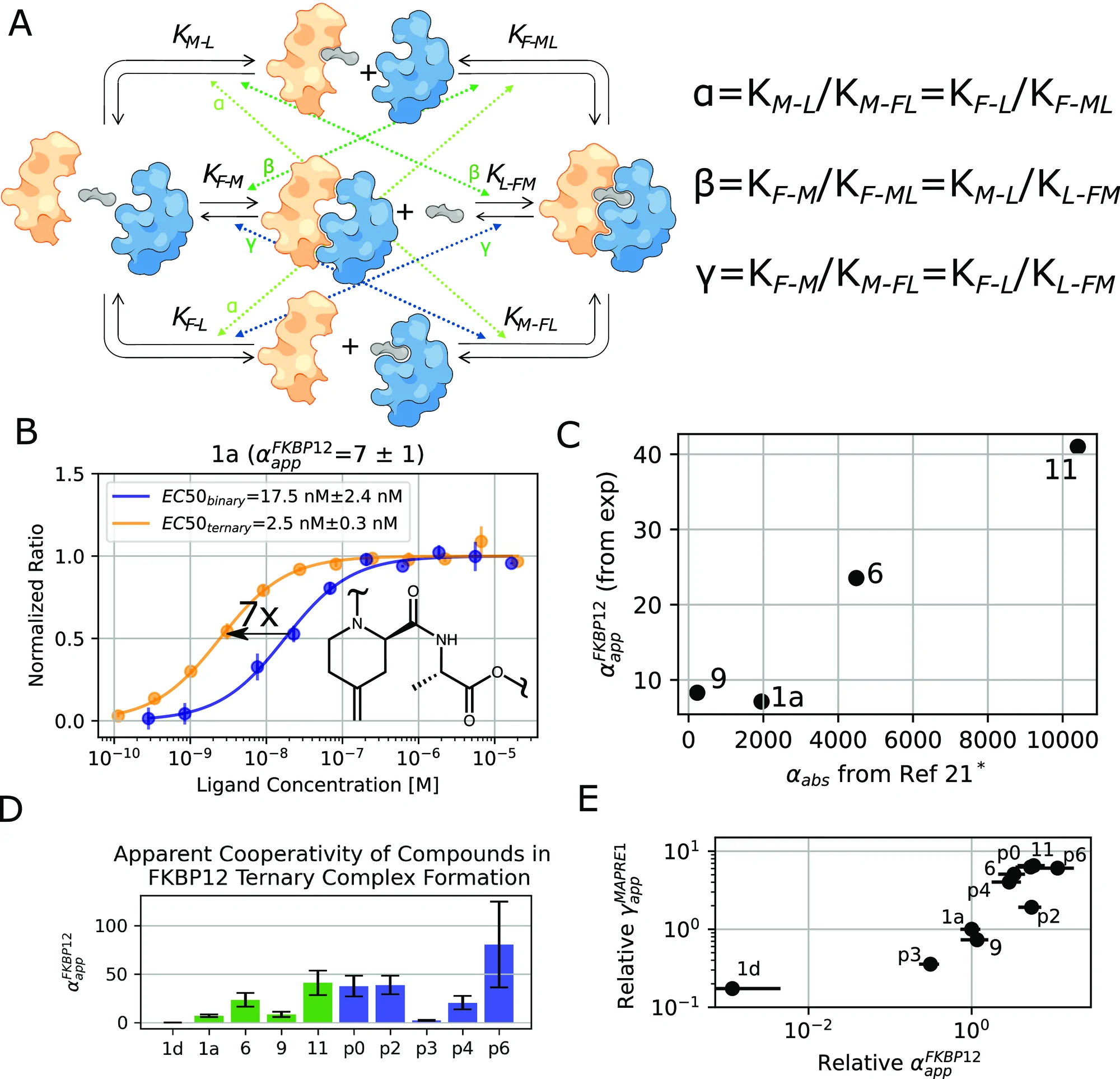
Experimental results of the prospective molecular glues for FKBP12-MAPRE1. (A): Schematic illustration of the different cooperativities α, β, and γ that can be retrieved for a ternary complex. (B): Ligand-response titration experiment with 40 nM labeled FKBP12 with 10 μM MAPRE1 (ternary assay in orange) and without MAPRE1 (binary assay in dark blue) of the lead compound **1a**. (C): Comparison between the apparent cooperativity $\alpha_{app}^{FKBP12}$ measured in the present work and the absolute cooperativity α*_abs_* taken from ref 21 (except **9**, see Table 1). (D): Apparent cooperativity $\alpha_{app}^{FKBP12}$ of the parent compounds (green) and the prospective compounds (blue) derived from the fitted EC50 values. Fitting of the experimental curves on other compounds and the non-normalized signals are discussed in Sections S6.3 and S6.4 in the Supporting Information. (E): Correlation between the relative apparent cooperativities $\alpha_{app}^{FKBP12}$ derived from the FKBP12-binding assays and $\gamma_{app}^{MAPRE1}$ derived from the MAPRE1-binding assays.

Besides the ligand cooperativity α, protein cooperativity γ can also be calculated (Figure 6A). Since the dissociation constant between the two proteins $K_{F-M}^{D}$ is independent of the compound, the relative value of the protein cooperativity is equivalent to the ratio of ternary dissociation constants $\gamma_{1}/\gamma_{2}=K_{FL_{2}-M}^{D}/K_{FL_{1}-M}^{D}$ . This gives us the opportunity to compare $\alpha_{app}^{FKBP12}$ with $\gamma_{app}^{MAPRE1}$ computed from the MAPRE1-binding assays using

1 $$\frac{\gamma_{app,1}^{MAPRE1}}{\gamma_{app,2}^{MAPRE1}}=\frac{EC50_{ternary,2}^{MAPRE1}}{EC50_{ternary,1}^{MAPRE1}}$$

where $EC50_{ternary,1}^{MAPRE1}$ and $EC50_{ternary,2}^{MAPRE1}$ are the EC50 values derived from the ternary titration experiments with MAPRE1. The MAPRE1-reporter EC50 ratio is used as an assay-derived relative apparent-cooperativity metric under the same assay conditions and is not interpreted as absolute thermodynamic cooperativity. In Figure 6E, we show a good correlation (Pearson coefficient *r* = 0.77) between the apparent cooperativities $\alpha_{app}^{FKBP12}$ and $\gamma_{app}^{MAPRE1}$ relative to the lead compound **1a** measured by different labeled proteins, providing a strong cross-assay concordance.

In summary, we achieved our goal to rationally design novel variants with higher cooperativity based on the observations from the MD simulations. These positive results highlight the power of considering a dynamic picture in the design of molecular glues.

## Conclusions

The rational design and optimization of molecular glues has long been hindered by the complexity of predicting the protein–protein interface dynamics. In this study, we demonstrated that the cooperativity of macrocyclic molecular glues for the FKBP12-MAPRE1 system cannot be adequately explained by static crystal structures alone. By employing MD simulations, we could formulate a putative pathway for the formation of the ternary complex and successfully designed variants of the lead compound **1a** with similar or higher cooperativity.

Our MD simulations suggest that the modifications of the 4-piperidine moiety fail to form the ternary complex because of the less favorable MM/GBSA interaction energies, which is consistent with weaker MAPRE1 engagement. Using enhanced sampling, we further revealed an intermediate conformation of the ligand, which is sampled in both the binary FKBP12-ligand and the ternary FKBP12-ligand-MAPRE1 ensembles. The observed ensemble overlap is consistent with a putative pathway involving conformational selection followed by structural relaxation, although the equilibrium simulations do not establish the kinetic sequence. By only considering the configurations in the intermediate state, which we call the stabilized configurational space, a high correlation (Spearman coefficient ρ = 0.90) was found between the computed interaction energy between the two proteins in the ternary complex and the experimentally measured absolute cooperativity. Using this model, we estimated the cooperativity of a library of 12 prospective variants of lead **1a** and selected five for synthesis and experimental testing. Of these five novel compounds, four showed higher apparent cooperativity than the parent lead compound **1a**, and three of them showed similar or greater apparent cooperativity than the previously most cooperative compound **11**.

To the best of our knowledge, this is the first study to elaborate on the conformational changes of molecular glues upon the third-body binding event and to prospectively design highly cooperative glues by establishing and targeting the stabilized configurational space shared between binary and ternary ensembles using MD simulations. We anticipate that this work will encourage researchers in the field to include considerations of protein and ligand dynamics in the design of cooperative molecular glues.

## Experimental Section

### Structure Preparation and MD Simulations

#### Conformer Generation

The initial conformations of the active molecular glue compounds were generated using the *ConstrainedEmbed* function of RDKit version 2023.03 ^28^ using as a template the conformation of **1a** in the crystal structure of the binary complex with FKBP12 (PDB ID: 9DCW^20^) and of the ternary complex (PDB ID: 9CO5 ^20^) respectively. The generated conformers were exported as an SDF file and converted to mol2 files and energy-minimized with the MMFF94 force field^29^ using OpenBabel 3.1.0.^30^ Because of the change in stereochemistry, inactive compounds **1d**, **2a**, **2c**, and **2d** were generated by the Builder in PyMOL v2.5.5 ^31^ and saved as a mol2 file for later parametrization.

#### Simulation Details

The initial coordinates of the complexes are taken from the crystal structures with ligand **1a** (PDB IDs: 9DCW^20^ and 9CO5 ^20^) either retained (**1a**), removed (apo system), or replaced (other compounds). The AMBER ff19SB force field^32^ was used for proteins, GAFF2 ^33,34^ for the ligands, OPC^35^ for water molecules, and Joung/Cheatham ion parameters^36^ for Na^+^ and Cl^–^ ions. Partial charges of the ligands were assigned with the AM1-BCC^37^ method implemented in the program *antechamber*.^38^

The constructed complexes were solvated in a water box with a length of 1.2 nm from the complex edge in each dimension with 0.15 M NaCl using the program *tleap* from AmberTools22.^34^ All simulations were performed with AMBER 22.^34^

Energy minimization was performed for a maximum of 5,000 cycles, comprising 2,500 steps of steepest descent followed by up to 2,500 steps of conjugate-gradient minimization. A positional restraint with a force constant of 418.4 kJ mol^–1^ nm^–2^ was applied to proteins and ligand atoms during minimization. Equilibration comprised two consecutive 125 ps NVT stages followed by two consecutive 125 ps NPT stages. A time step of 1 fs was used during equilibration. Positional-restraint force constants of 418.4, 209.2, 209.2, and 41.84 kJ mol^–1^ nm^–2^ were applied to protein and ligand atoms during the four successive equilibration stages. Following equilibration, three production replicas were initiated from the final restart file of the second NPT stage. Although the replicas shared the same initial coordinates and velocities, they were propagated using independently initialized Langevin random streams generated with the AMBER 22 default setting ig = −1.

Simulations were carried out with a Langevin thermostat^39^ to keep the temperature at 295.15 K with a collision frequency of 1.0 ps^–1^ and a Monte Carlo barostat^34^ to keep the pressure at 1 bar. A nonbonded cutoff distance of 1 nm was used with PME^40,41^ to treat the long-range electrostatic interaction. The simulations were integrated with a time step of 2 fs, using the SHAKE algorithm^42^ to constrain the bond lengths of bonds involving hydrogen with a relative geometrical tolerance of 10^–6^. The translational and rotational center-of-mass motion was removed every 1000 steps. Trajectories were printed every 500 ps in the production simulation for analysis.

#### Conventional Simulations

Each system was submitted to a 1 μs production run from the second NPT equilibrated structure.

#### REST2 Enhanced-Sampling Simulations

For the enhanced-sampling simulations, the REST2 ^22^ implementation in Amber22 ^34^ called *Replica Exchange with Arbitrary Degree of Freedom (REAF)* was used by setting the flag *gti_add_re = 6*. The scaling was only applied on the ligand degrees of freedom. A scaling factor (1-τ) between 1 and 0.4 was used with an even spacing in the linear scale between 12 replicas, i.e., τ_*k*_ = {0.000, 0.05454, 0.10908, 0.16362, 0.21816, 0.27270, 0.32724, 0.38178, 0.43632, 0.49086, 0.54540, 0.59994}, where *k* indicates the replica index, was used for the binary and ternary complex simulations, and a linear scale between 14 replicas ranging between 1 and 0.25, i.e., τ_*k*_ = {0.000, 0.05769, 0.11538, 0.17307, 0.23076, 0.28845, 0.34614, 0.40383, 0.46152, 0.51921, 0.57690, 0.63459, 0.69228, 0.74997}, was used for the ligand-only simulations. The enhanced-sampling simulations were initiated from the last frame of the second NPT equilibration and propagated for 200 ns with replica exchange attempted at every 100 steps. The exchange acceptance ratios were around 35% in the ligand-only and binary complexes, and around 20% in the ternary complexes between all neighboring replicas. The per-replica-pair exchange ratio is shown in Figure S37 in the Supporting Information. Trajectories of the REST2 simulations were printed every 20 ps. The analyses on the REST2 simulations were performed on the physical replica (τ_0_ = 0).

### Analysis

#### “Native” and “Nonnative” Protein–Protein Contacts

From the crystal structure of the ternary complex with compound **1a**, the “native” contacts between the two proteins were defined as the number of atoms from one protein within 5 Å of any heavy atom of the second protein using the *nativecontact* command from cpptraj in AmberTools22,^34^ which provides both native and nonnative contact counts. The same procedure was applied to the frames of the MD simulations. If an identified contact is not part of the “native” set obtained from the crystal structure, it was counted as “nonnative”. The native contact ratio was calculated as *N*_*nat*_/(*N*_*nat*_ + *N*_*non‑nat*_), where *N*_*nat*_ and *N*_*non‑nat*_ are the number of native and nonnative contact atoms. It indicates whether the interactions between the two proteins are similar to the ones observed in the ternary crystal structure.

#### Inter-Core Angle of the Ligand Conformation

As a simplified descriptor of the bending motion of the MAPRE1-binding core of the ligand observed between the binary and ternary crystal structures, the angle between the MAPRE1-binding core and the FKBP12-binding core (Figure 1A) was calculated by measuring the angle between the two fitting planes, termed the intercore angle θ_*lig*_. A detailed description and illustration of the plane-fitting procedure is provided in Section S3 in the Supporting Information.

#### Pairwise Interaction Energy with MM/GBSA

Molecular mechanics coupled generalized-Born and surface area (MM/GBSA)^43,44^ is an end-point energy evaluation method that estimates the system energy by replacing the noisy explicit water molecules with an implicit solvent continuum. The pairwise interaction energy between entities *A* and *B* in the ensemble X is calculated as,

2 $${\left\langle \Delta E_{A-B}^{int}\right\rangle }_{X}={\left\langle E_{AB}\right\rangle }_{X}-{\left\langle E_{A}\right\rangle }_{X}-{\left\langle E_{B}\right\rangle }_{X}$$

where ${\left\langle \cdot \right\rangle }_{X}$ denotes the ensemble average in X and *E*_*AB*_ is the potential energy of a complex configuration. The potential energy of the system was evaluated with the classical force fields, the GB implicit solvent model,^45^ and the solvent-accessible surface-area (SASA) for the solute–solvent interactions. GB and SASA were computed with the OBC-2 model^34,45^ (*igb = 5* in Amber22) and GB radii assigned by *mbondi2* using MMPBSA.py^46^ and ante-MMPBSA.py from AmberTools22.^34^

In case of the conventional MD simulations, only the last 800 ns were used for the MM/GBSA energy evaluation, whereas the last 180 ns were used in case of the REST2 simulations.

#### Statistical Analysis

Active and inactive compounds were compared at both the compound and interface levels. For the primary compound-level analysis, the six interface values obtained from three simulations with two interfaces each were averaged for each compound, producing four compound-level observations per activity group. For the exploratory interface-level analysis, the six interface values from each compound were treated as independent dynamical realizations, producing 24 observations per activity group. Two-sided Welch’s *t*-tests were performed using the scipy.stats.ttest_ind function of the Python package *scipy* ^47^ with equal_var = False. The corresponding *p*-values are denoted *p*_comp_ and *p*_int_, respectively.

#### Calculation of the Induced PPIs in the Stabilized Configurational Space

The stabilized configurational space was defined as the configurations in the ternary complex simulations that are also seen in the simulations of the monomers and binary complexes, respectively.^23^ The high rigidity of FKBP12 in both binary and ternary complexes allows us to approximate the stabilized configurational space using a frame-to-frame alignment scheme as a geometric filter.

For this, each frame from the ternary-complex simulation (coordinates **r**_*FLM*_) was aligned to each frame of the binary complex simulation (coordinates **r**_*FL*_) using FKBP12 as reference. Based on the aligned frames, the minimal distance between the ligand from coordinates **r**_*FL*_ and MAPRE1 from coordinates **r**_*FLM*_ was calculated as $d_{min}^{L-M}$ . The geometric compatibility fraction of a configuration **r**_*FLM*_ was,

3 $$P^{acc}\left(r_{FLM}\right)={\left\langle \chi \left(d_{min}^{L-M}-d_{cut}^{L-M}\right)\right\rangle }_{FL}$$

where $d_{cut}^{L-M}$ is the cutoff distance for the indicator function $\chi \left(d_{min}^{L-M}-d_{cut}^{L-M}\right)$ , which returns 1 if $d_{min}^{L-M}>d_{cut}^{L-M}$ or 0 otherwise. $d_{cut}^{L-M}=0.26\;\mathrm{nm}$ was used based on the analysis of the FLM ensembles (see Section S5.2 in the Supporting Information). If *P* ^*acc*^(r_*FLM*_) was higher than 1%, the configuration was considered part of the stabilized configurational space (1% was determined manually). Sensitivity analysis at the threshold of *P* ^*acc*^ is shown in Figure S42 in the Supporting Information. With the stabilized configurational space in hand, the interaction energy between the two proteins was calculated using eq 2. Examples of the alignment process are provided in Figure S41 in Section S5.2 in the Supporting Information.

### Compound Synthesis

Compounds **1a**, **1d**, **6**, **9**, and **11** were resynthesized by WuXi AppTec according to the synthesis route described in ref 20.

Novel compounds **p0**, **p2**, **p3**, **p4**, and **p6** were prepared in a similar manner. The detailed synthesis description is provided in Section S7 in the Supporting Information. The crude products were purified by preparative supercritical fluid chromatography (SFC). The identity and purity were confirmed by liquid chromatography–mass spectrometry (LC–MS) and ^1^H NMR spectroscopy (Sections S8 and S9 in the Supporting Information).

### Spectral Shift Assays

Spectral shift assays were performed by Crelux.

#### Protein Labeling

MAPRE1 (FVD1, PC24359–1) was labeled using an NHS-NT650 fluorophore according to the manufacturer’s instructions. Briefly, 20 μM MAPRE1 was incubated with 60 μM NHS-NT650 dye in NHS labeling buffer supplemented with 0.01% Pluronic for 30 min at room temperature. Following labeling, samples were centrifuged for 10 min at 1500 g to remove aggregates. The degree of labeling was determined to be approximately 86%.

FKBP12 (CKU8, PD24502–1) was labeled using RED-tris-NTA second generation dye, exploiting the affinity of the tris-NTA moiety for the His-tag. For labeling, FKBP12 was incubated with RED-tris-NTA dye for 20 min at room temperature, followed by centrifugation for 10 min at 15000 g prior to use.

### Binary Binding Assays

#### Compound Preparation

All compounds were prepared as stock solutions in dimethyl sulfoxide (DMSO). For MAPRE1-binding experiments, a 1:2 serial dilution series consisting of 12 concentrations was prepared, starting from a highest compound concentration of 400 μM. For FKBP12 binding experiments, a 1:3 serial dilution series of 12 concentrations was prepared, starting from a highest compound concentration of 50 μM. Final DMSO concentrations were maintained at 2% (v/v) in all assay conditions.

#### Assay Setup

All measurements were performed in assay buffer consisting of 20 mM HEPES (pH 7.5), 150 mM NaCl, 1 mM DTT, 0.01% Pluronic (nonionic triblock copolymers). Compound dilutions were mixed with concentrated labeled protein solutions to yield the final assay concentrations. Each condition was prepared in duplicate technical replicates within the same experiment. For FKBP12-binding assays, 80 nM FKBP12 was incubated with 10 nM RED-tris-NTA dye and subsequently assayed at a final concentration of 40 nM FKBP12 in the presence of 5 nM NTA fluorophore. For MAPRE1-binding assays, the labeled protein concentration was 5 nM.

After equilibration at room temperature, samples were measured at LED powers of 39.9%–100% depending on protein concentration (39.9% for 5 nM MAPRE1, 80.9% for 40 nM FKBP12). Fluorescence emission ratios at 670 and 650 nm were recorded. The ratio (670 nm/650 nm) was plotted as a function of compound concentration to determine EC50.

### Ternary Binding Assays

#### FKBP12-Labeled Binding Assay

Labeled FKBP12 (CKU8, PD24502–1) was used as the fluorescent reporter protein at a final concentration of 40.0 nM. MAPRE1 (FVD1, PC24359–1) was added as an unlabeled binding partner at a fixed concentration of 10 μM.

The assay buffer consisted of 20 mM HEPES (pH 7.5), 150 mM NaCl, 1 mM DTT, and 0.01% Pluronic F-127. Final DMSO concentrations were maintained at 2% (v/v) across all conditions. All compounds were prepared as DMSO stock solutions and assayed as 1:3 serial dilution series consisting of 12 concentrations, starting from a highest compound concentration of 20.0 μM and decreasing to 112.9 pM. Compound dilutions were mixed with concentrated labeled FKBP12 and MAPRE1 solutions to achieve the final assay concentrations. Each condition was measured once in duplicate technical replicates within the same experiment.

All measurements were performed on a Dianthus NT.23 PicoDuo instrument using an LED power of 77.6% (pico detector setting). Fluorescence emission ratios at 670 and 650 nm were recorded. The spectral shift ratio (670 nm/650 nm) was plotted as a function of compound concentration to assess ternary complex formation and to determine apparent binding affinities.

#### MAPRE1-Labeled Binding Assay

Ternary binding interactions were additionally characterized using fluorescently labeled MAPRE1 at a final concentration of 5.0 nM in the assay.

Ternary complex formation was assessed in the presence of unlabeled FKBP12 (CKU9, PD24504–1), which was included directly in the assay buffer at a fixed final concentration of 10 μM. The assay buffer consisted of 20 mM HEPES (pH 7.4), 150 mM NaCl, 1 mM DTT, and 0.01% Pluronic F-127. The final DMSO concentration was maintained at 2% (v/v) in all samples. Compounds were prepared as DMSO stock solutions and tested in a 1:3 serial dilution series comprising 12 concentrations, starting from a highest final compound concentration of 50 μM and decreasing to 282.3 pM. Compound dilutions were mixed with labeled MAPRE1 and FKBP12-containing assay buffer to reach the indicated final concentrations. Each condition was measured once in duplicate technical replicates within the same experiment.

All measurements were performed on a Dianthus NT.23 PicoDuo instrument. The LED power was set to 26.9% (pico detector setting), and fluorescence emission was recorded at 650 and 670 nm. Changes in the 670 nm/650 nm ratio were used to monitor ternary complex formation and to determine apparent binding affinities.

#### Binding Curve Fitting

Data points from the spectral shift assays with a standard deviation across the two replicates greater than 10% of the min-max difference were discarded to prevent instabilities during fitting. Because of the low ligand-MAPRE1 affinity, as shown in ref 21 and Section S6.2 of the Supporting Information, and because no downturn was observed within the fitted concentration range, the ternary hook effect is neglected. The measured signal was treated as the binary binding event and fitted to a Hill binding equation,

4 $$S=S_{min}+\left(S_{max}-S_{min}\right)\times \frac{C}{C+EC50}$$

with *S*_*min*_ and *S*_*max*_ being the minimal and maximal signals and *C* the titrant concentration. The signal was normalized by,

$$\mathrm{S̅}=\frac{S-S_{min}}{S_{max}-S_{min}}$$ 5

The fitting was performed with the *optimize.curve_fit* function of the Python package *scipy* ^47^ that minimizes the least-squares loss.

Apparent cooperativities were calculated using,

6 $$\alpha_{app}=\frac{EC50_{binary}}{EC50_{ternary}}$$

The standard error σ of the *EC*50 value was estimated from the fitting, and the error of the apparent cooperativity was computed using error propagation,

7 $$\sigma \left(\alpha_{app}\right)=\alpha_{app}\sqrt{{\left(\frac{\sigma \left(EC50_{binary}\right)}{EC50_{binary}}\right)}^{2}+{\left(\frac{\sigma \left(EC50_{ternary}\right)}{EC50_{ternary}}\right)}^{2}}$$

As the binary and ternary measurements were performed in separate experiments, eq 7 assumes zero covariance. The resulting uncertainties are propagated fitting standard errors and do not include between-experiment variability.

## Supplementary Material



## Data Availability

Topologies, coordinates of the minimized structures in PDB format, simulation and analysis scripts, and experimental data (.csv) are available at the GitHub repository: https://github.com/rinikerlab/MAPRE1-FKBP12-Molecular-Glue.

## Notes

Experimental data for compounds **9**, **2c**, and **2d**; time evolution of the protein–protein contacts and interaction energy; intercore angle fitting; convergence analysis in conventional MD and REST2 simulations; details of frame-to-frame alignment to determine stabilized geometries; details on the titration experiments for binary and ternary assays; synthesis details of prospective compounds; LC-MS chromatograms and ^1^H NMR spectra of prospective compounds (PDF)
