# Supplementary material for: Designing Cooperative Molecular Glues for FKBP12-MAPRE1 with Enhanced-Sampling Molecular Dynamics Simulations
Source: J Am Chem Soc. 2026 Oct 1;148(40):43321–33. doi: 10.1021/jacs.6c12127 (PMC13674235; doi:10.1021/jacs.6c12127)
Supplement: Supplementary file 1 [file ja6c12127_si_001.pdf]

SUPPORTING INFORMATION
Designing Cooperative Molecular Glues for FKBP12-MAPRE1 with Enhanced-Sampling Molecular Dynamics Simulations

Shu-Yu Chen,[a,c] Riccardo Solazzo,[a,c] Marianne Fouché,[b] Hans-Jörg Roth,[b] Birger Dittrich[b] and Sereina Riniker*[a]

[a] *Department of Chemistry and Applied Biosciences, ETH Zürich, Vladimir-Prelog-Weg 2, 8093 Zürich, Switzerland.*
[b] *Novartis Biomedical Research, Novartis Campus, 4002 Basel, Switzerland*
[c] *These authors contributed equally.*
E-mail: sriniker@ethz.ch

Contents

S1 Binding Measurement of Compounds 1a, 2c, 2d, and 9 S3
S1.1 Compounds 1a, 2c, and 2d . . . S3
S1.2 Apparent Cooperativity of Compound 9 from MAPRE1-Binding Assay . . . S3

S2 Additional Analysis on the Simulations of the Ternary Complexes S4
S2.1 Time Evolution of the Protein-Protein Contacts and Interaction Energy . . . S4
S2.2 RMSD of the Ternary Components in the Unbiased MD Simulations . . . S7

S3 Inter-Core Angle Fitting S7
S3.1 Inter-core Angle Definition . . . S7
S3.2 Correlation Between the MAPRE1 Tilting Angle and the Ligand Core-to-Core Angle . . S8

S4 Convergence in Conventional MD and REST2 Enhanced Sampling S9
S4.1 Conventional MD Simulations Show Kinetic Traps . . . S9
S4.2 Fast θ_{lig} Transitions Observed in REST2 Simulations . . . S10
S4.3 Additional Convergence Check of the REST2 Simulations . . . S13

S5 Determination of Stabilized Geometries from the Binary and Ternary Complex Simulations by Frame-to-Frame Alignment S38
S5.1 Oversimplification of the θ_{lig} Representation . . . S38
S5.2 Low Flexibility of FKBP12 in FL and FLM Complexes . . . S39

S6 Computational Results and Titration Experiments for Binary and Ternary Assays for the Prospective Compounds S42
S6.1 θ_{lig} Distribution of the Prospective Compounds . . . S42
S6.2 Titration Experiment with MAPRE1-Binding Assay . . . S42

S6.3 EC50 Fitting of the FKBP12-Binding Titration Data S43
S6.4 Non-normalized Fitting Curves and Filtered Data Points S44

S7 Synthesis of Prospective Compounds **S47**
S7.1 Synthesis of Compound p0 . . . . S47
S7.2 Synthesis of Compound p2 . . . . S49
S7.3 Synthesis of Compound p3 . . . . S50
S7.4 Synthesis of Compound p4 . . . . S52
S7.5 Synthesis of Compound p6 . . . . S54

S8 LC-MS of Prospective Compounds **S56**

S9 ^{1}H-NMR Spectra of Prospective Compounds **S66**

S10Analytical Characterization of Final Compounds **S69**

S1 Binding Measurement of Compounds 1a, 2c, 2d, and 9

S1.1 Compounds 1a, 2c, and 2d

The ability of compounds **1a**, **2c**, and **2d** to induce interactions between FKBP12 and MAPRE1 was measured in the same way as described in Ref. [1]. Ligands were titrated over the indicated concentration range and assayed either for binary FKBP12 binding or for FKBP12-MAPRE1 ternary-complex formation. In the binary FKBP12-binding assay, decreasing activity indicates concentration-dependent displacement of the FKBP12-binding tracer. All three compounds reduce the assay signal, indicating that **1a**, **2c**, and **2d** engage FKBP12 to different extents (Figure S1, left).

In contrast, the ternary recruitment assay distinguishes FKBP12 binding from productive FKBP12-MAPRE1 recruitment. In this assay, increased activity reflects compound-induced proximity between FKBP12 and MAPRE1. Compound **1a** produces a clear concentration-dependent increase in activity, consistent with molecular-glue-mediated formation of the FKBP12:**1a**:MAPRE1 complex described in Ref. [1]. By comparison, **2c** and **2d** remain close to baseline across the concentration range, despite showing binary FKBP12 binding. These data therefore indicate that binary FKBP12 engagement alone is not sufficient for MAPRE1 recruitment, and that productive ternary-complex formation is specific to **1a** under these assay conditions (Figure S1, right).

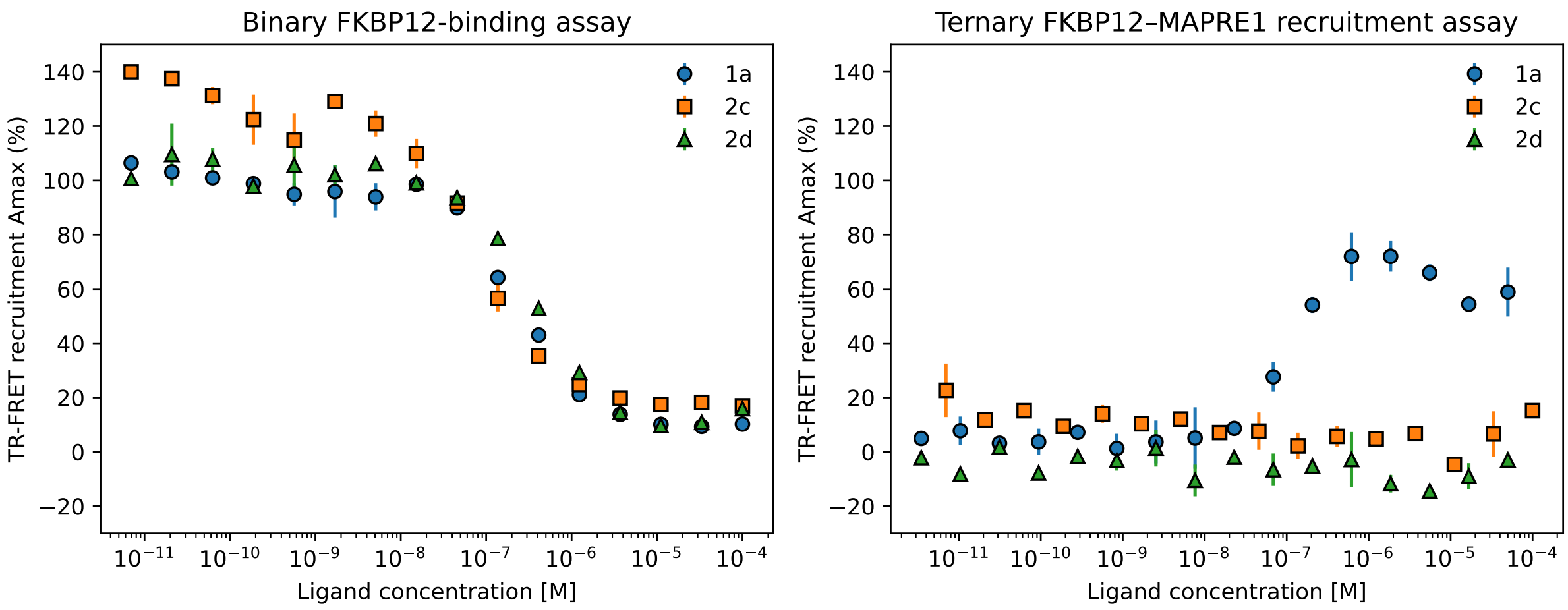

Figure S1: Binary FKBP12-binding (left) and ternary FKBP12-MAPRE1 recruitment (right) responses for compounds **1a** (blue), **2c** (orange), and **2d** (green). Data points show mean activity values, and error bars indicate the standard deviation of replicate measurements.

S1.2 Apparent Cooperativity of Compound 9 from MAPRE1-Binding Assay

The cooperativity of compound **9** was measured in the same way as described in Ref. [2]. The EC_{50} of the %$\text{FKBP12}_{\text{bound}}$ binary curve is 186 nM. Correcting for 50% of the total FKBP12 concentration ($[FKBP12_{\text{tot}}] = 40$ nM) yields $K_{d,\text{FKBP12,binary}} = 166$ nM. When the experiment was performed in the presence of 10 μM MAPRE1, probing ternary complex formation via FKBP12, the EC_{50} of the %$\text{FKBP12}_{\text{bound}}$ ternary curve is 110 nM, corresponding to an apparent cooperativity (EC50 shift) of 1.7. This value underestimates the cooperativity inferred from MAPRE1-based measurements, consistent with trends observed for the other compounds. The EC_{50} of the %$\text{MAPRE1}_{\text{bound}}$ binary curve is 4.6×10^5 nM.

As the total MAPRE1 concentration is low ($[MAPRE1_{tot}] = 5$ nM), this EC_{50} was taken directly as $K_{T,1}$. Addition of 10 μM FKBP12 shifts the EC_{50} of the %$MAPRE1_{bound}$ ternary curve to 1985 nM, corresponding to an apparent cooperativity of 232.

Based on these results and together with data from the other compounds reported in Ref. [2], the apparent cooperativity measured in the MAPRE1-binding assay provides a reliable proxy for intrinsic cooperativity. Accordingly, we use MAPRE1 apparent cooperativity when comparing active and inactive compounds.

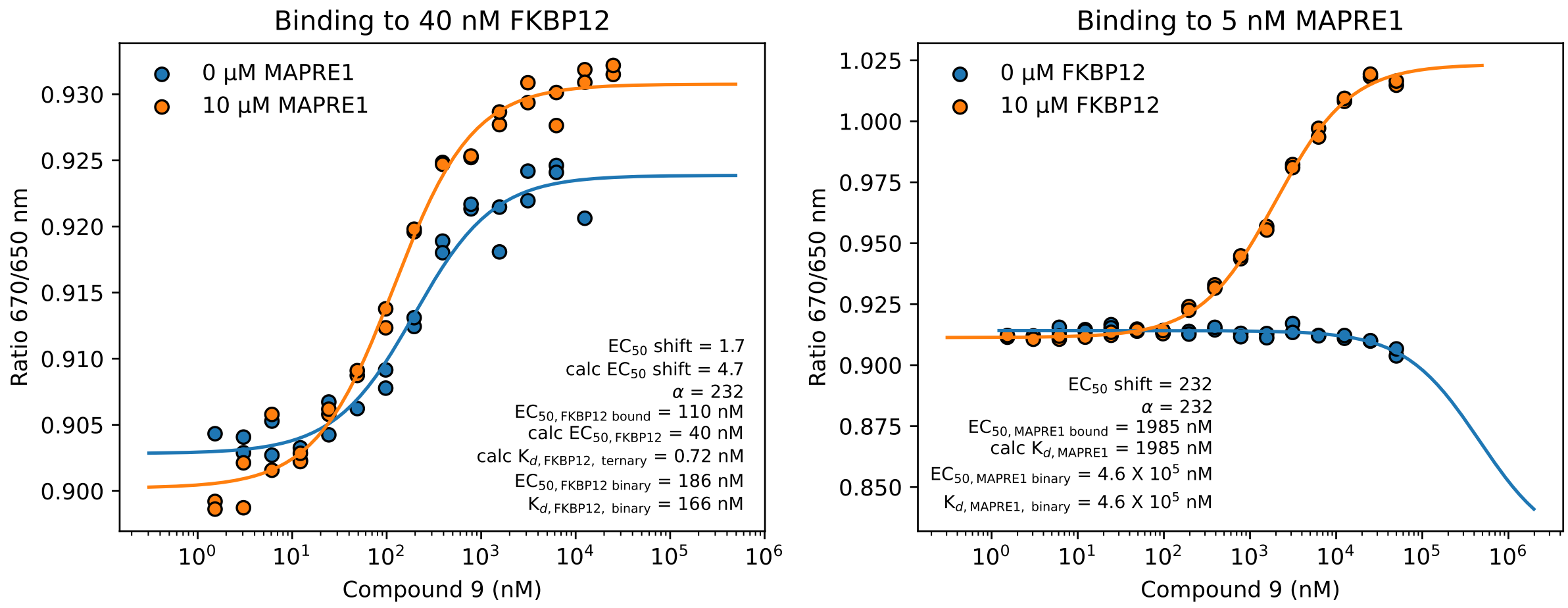

Figure S2: Binding data with experimentally measured and calculated apparent cooperativity and K_d values for compound **9**.

S2 Additional Analysis on the Simulations of the Ternary Complexes

S2.1 Time Evolution of the Protein-Protein Contacts and Interaction Energy

In the main text, we used box plots with the average value of the six interface trajectories sampled in the FLM complexes. In Figure S3 and Figure S4, we show the time evolution of the PPI contacts and the interaction energy evaluated by MM/GBSA, respectively.

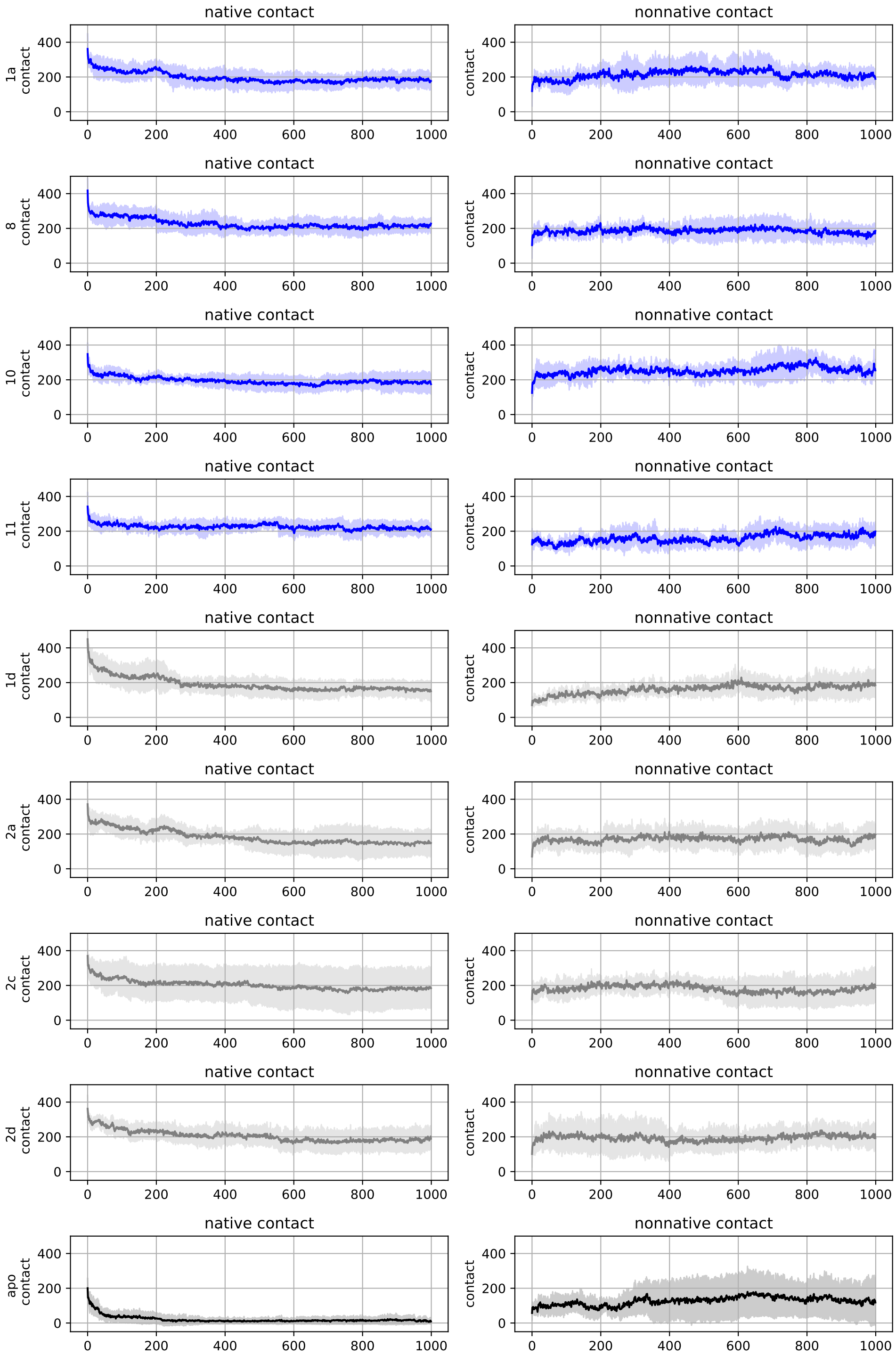

Figure S3: Time evolution of the native and nonnative contacts in the 1 μs conventional MD simulations. The solid lines and shaded areas show the average and standard deviation of the corresponding feature at time t over the six interfaces, respectively.

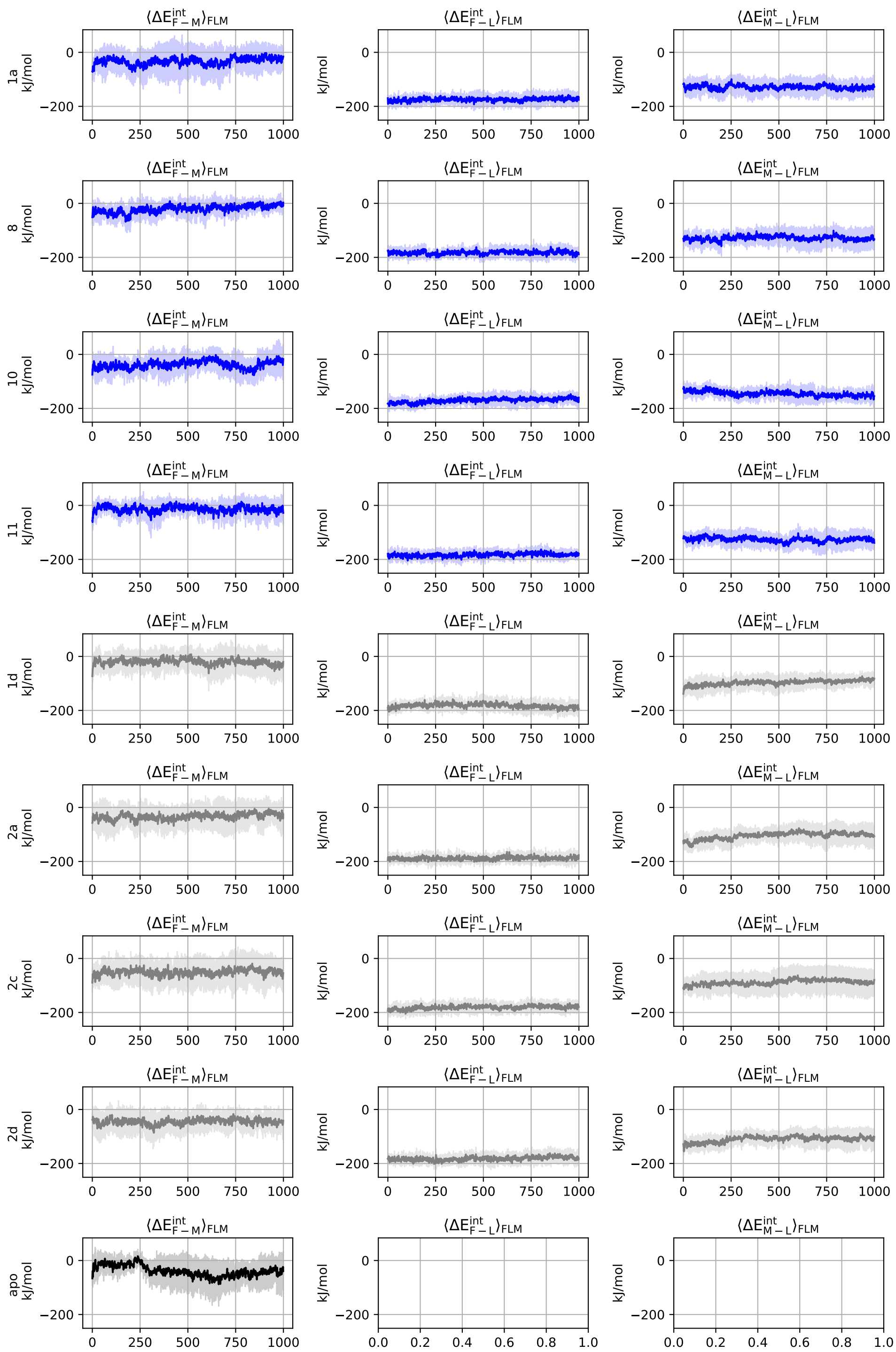

Figure S4: Time evolution of the pairwise interaction energy computed with MM/GBSA for the 1 μs conventional MD simulations. The solid lines and shaded areas show the average and standard deviation of the corresponding feature at time t over the six interfaces, respectively.

S2.2 RMSD of the Ternary Components in the Unbiased MD Simulations

In addition to the contact and energy analysis, the RMSD of FKBP12, MAPRE1, and the ligand in each ternary complex simulation at each interface is also reported in Figure S5. No ligand dissociation was observed from any interface within 1 μs. This is expected as the FKBP12 binding motif of the lead compound 1a is not modified in any of the variants. This is also supported by the nanomolar binary K_D values for FKBP12 (see Figure S1 and Ref. 1). In contrast, the Cα-RMSD of MAPRE1 shows the biggest difference between the active and inactive compounds with $p < 0.05$ at both the compound level and interface level.

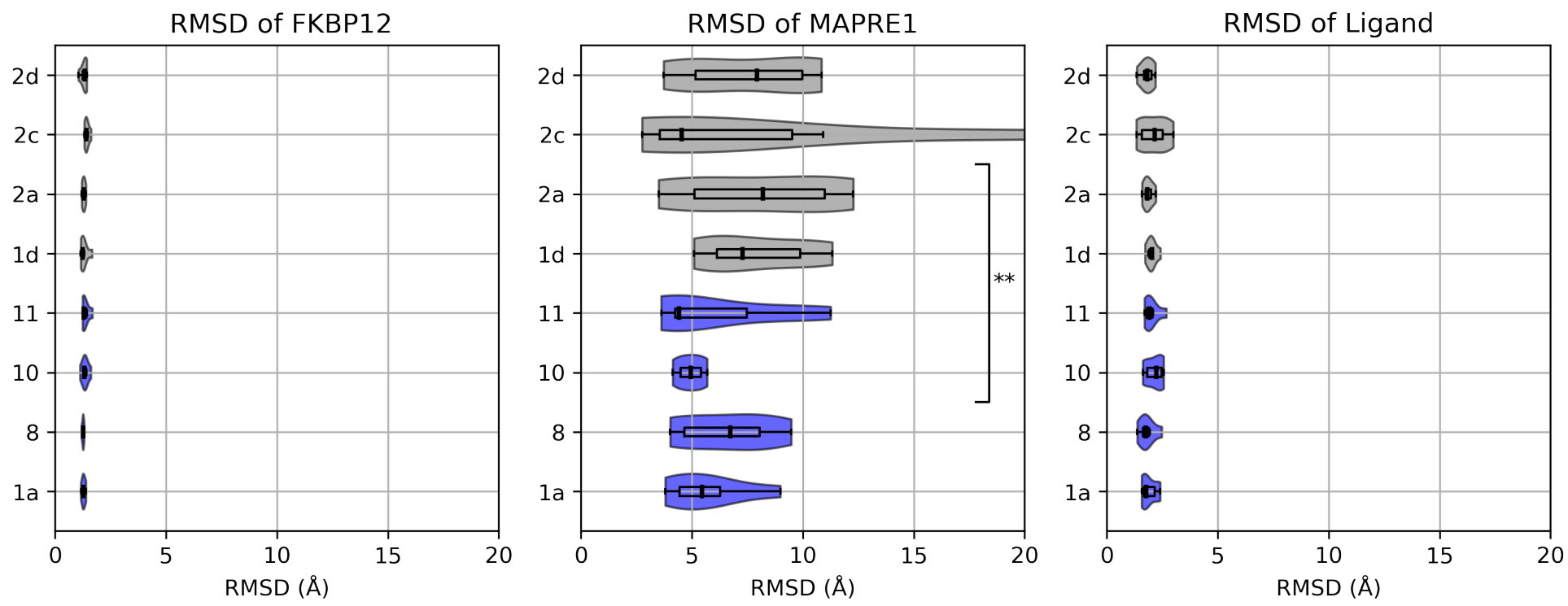

Figure S5: Root-mean-square deviation (RMSD) of the FKBP12 Cα atoms ($p_{\text{comp}} = 0.6386$, $p_{\text{int}} = 0.5711$), MAPRE1 Cα atoms ($p_{\text{comp}} = 0.00732$, $p_{\text{int}} = 0.0398$), and the ligand ($p_{\text{comp}} = 0.867$, $p_{\text{int}} = 0.8753$) at each interface, calculated after aligning the FKBP12 Cα atoms of the corresponding interface. The violin plots show the distributions obtained from three simulations with two interfaces per simulation ($N = 6$ per compound). The overlaid box plots show the median $\pm$ 1.5 $\times$ interquartile range. The p_{comp} and p_{int} values correspond to comparisons between the active and inactive compounds at the compound and interface levels, respectively.

S3 Inter-Core Angle Fitting

S3.1 Inter-core Angle Definition

To simplify the conformational analysis of the macrocycles, the inter-core angle was calculated as the angle between the two fitting planes on the FKBP12-binding core (colored blue in Figure S6) and the MAPRE1-recognition core (colored red in Figure S6). In addition, to distinguish the direction of bending, the 1,2-dimethoxybenzene group (colored yellow in Figure S6) was used to assign the sign of the angle.

First, the two atoms shared by both cores were aligned with the positive x-axis and centered at the origin. Each core was then fitted independently to a plane constrained to pass through the origin. This ensures that the planes fitted on both cores pass through the origin and intersect at the x-axis. Such a plane can be described by three coefficients:

$$ax + by + cz = 0, \tag{1}$$

from which the normal vector of the plane, $\hat{\mathbf{n}} = \frac{1}{\sqrt{a^2+b^2+c^2}}(a, b, c)$, is defined. The Euclidean distance between any point $\mathbf{p} = (x_p, y_p, z_p)$ and this plane is:

$$d_p = |\hat{\mathbf{n}} \cdot \mathbf{p}|. \tag{2}$$

The coefficients of each plane were obtained by minimizing the root-mean-square distances (RMSD) from the corresponding core atoms. After the FKBP12-fitting plane was constructed, the molecule was rotated so that the normal vector was aligned with the z-axis, with the 1,2-dimethoxybenzene group placed at $z > 0$ (colored yellow in Figure S6). Lastly, the MAPRE1-recognition core was fitted, and the inter-core angle was determined by the z-component of the normal vector and the sign of the z-component of the center-of-mass of the MAPRE1-recognition core (COM^z_{MAPRE1}):

$$\theta_{\text{lig}} = \arccos\left(\frac{c_{\text{MAPRE1}}}{\sqrt{a^2_{\text{MAPRE1}} + b^2_{\text{MAPRE1}} + c^2_{\text{MAPRE1}}}}\right) \cdot \text{sign}\left(\text{COM}^z_{\text{MAPRE1}}\right). \tag{3}$$

This means that a positive θ_{lig} is assigned when the MAPRE1-recognition core is on the same side of the FKBP12-fitting plane as the 1,2-dimethoxybenzene group; otherwise, it is negative.

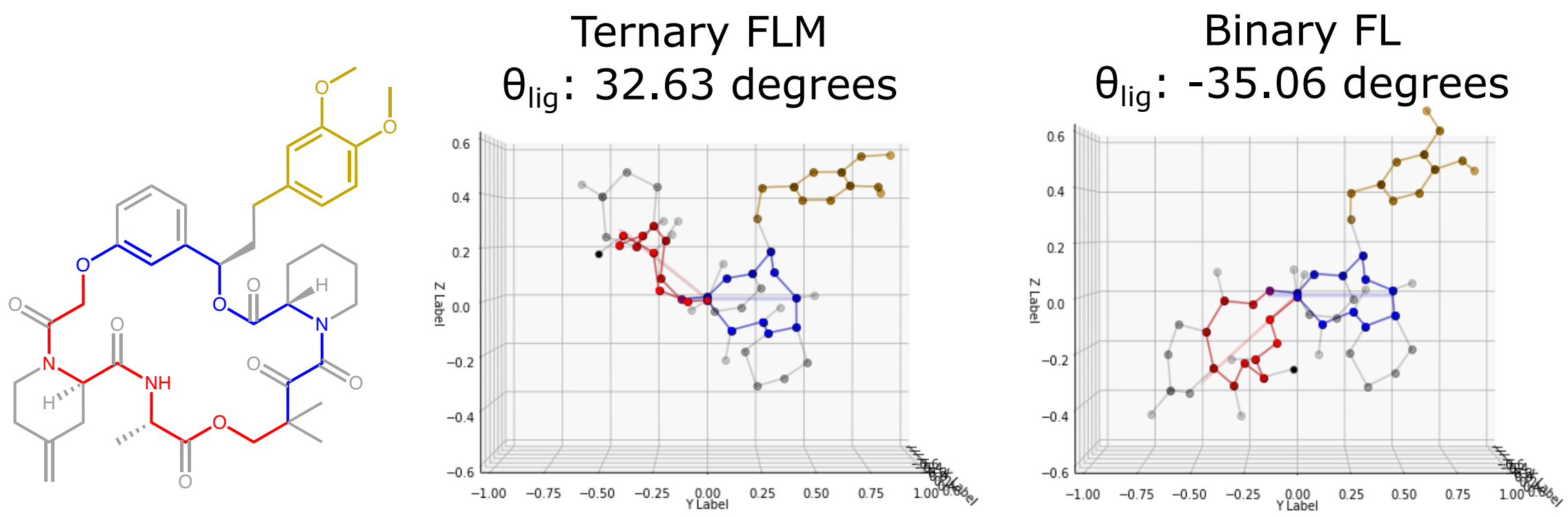

Figure S6: Schematic illustration of the plane-fitting protocol to compute θ_{lig} using compound **1a**. The FKBP12-plane (blue plane) is always aligned with the z-axis, with the 1,2-dimethoxybenzene group (yellow) being placed in the positive z-region. The z-component of the MAPRE1-fitting plane normal vector determines θ_{lig}, where the sign is defined by the location of the center-of-mass of the MAPRE1-recognition core (red). Examples of the fitting of a conformation from a MD snapshot from the ternary FLM and binary FL simulations with the ligand conformation in the ternary (PDB ID: 9CO5) and binary (PDB ID: 9DCW) crystal structures, respectively.

S3.2 Correlation Between the MAPRE1 Tilting Angle and the Ligand Core-to-Core Angle

In the main text, we showed that the rise of the tilting angle of MAPRE1 with respect to the FKBP12-**1a**, characterized by the angle θ_{PPI}, coincides with the decrease of the ligand core-to-core angle θ_{lig}. In Figures S7 and S8, we show the correlations measured by Spearman's ρ in the conventional MD simulations for the four active compounds (1a, 8, 10, and 11) and the REST2 simulation for the eight active compounds (1a, 3, 6, 7, 8, 9, 10, and 11), respectively. While moderate anti-correlations in conventional MD (Spearman's ρ around -0.4) and weak anti-correlations in REST2 (Spearman's ρ around -0.2) were observed in most of the compounds, weak correlations can be found because of the rare population of the tilted state (high θ_{PPI} and θ_{lig}).

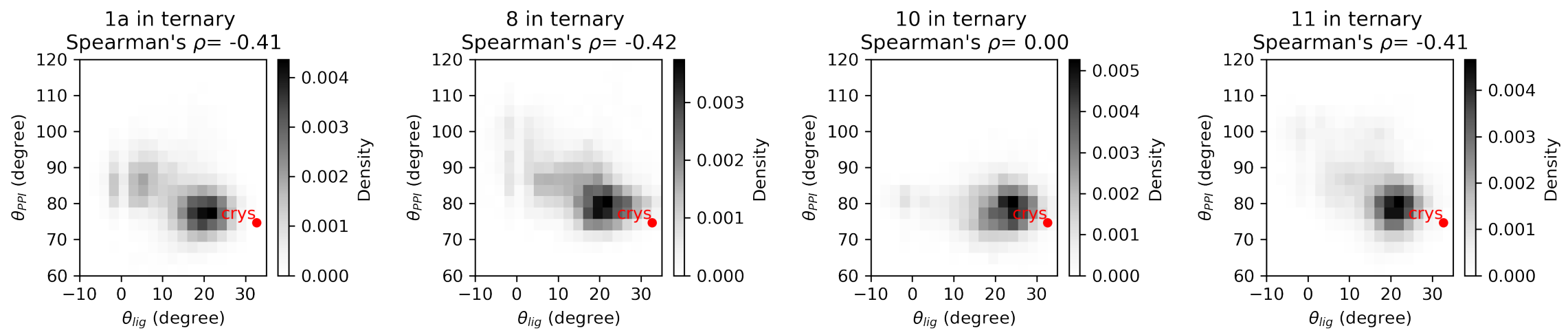

Figure S7: Correlation between the MAPRE1 tilting angle, with respect to FKBP12, and the ligand core-to-core angle observed from the three 1 μs unbiased MD simulations of the four active compounds (1a, 8, 10, and 11). The red point indicates angles measured from the FKBP12-1a-MAPRE1 crystal structure (PDB ID 9CO5).

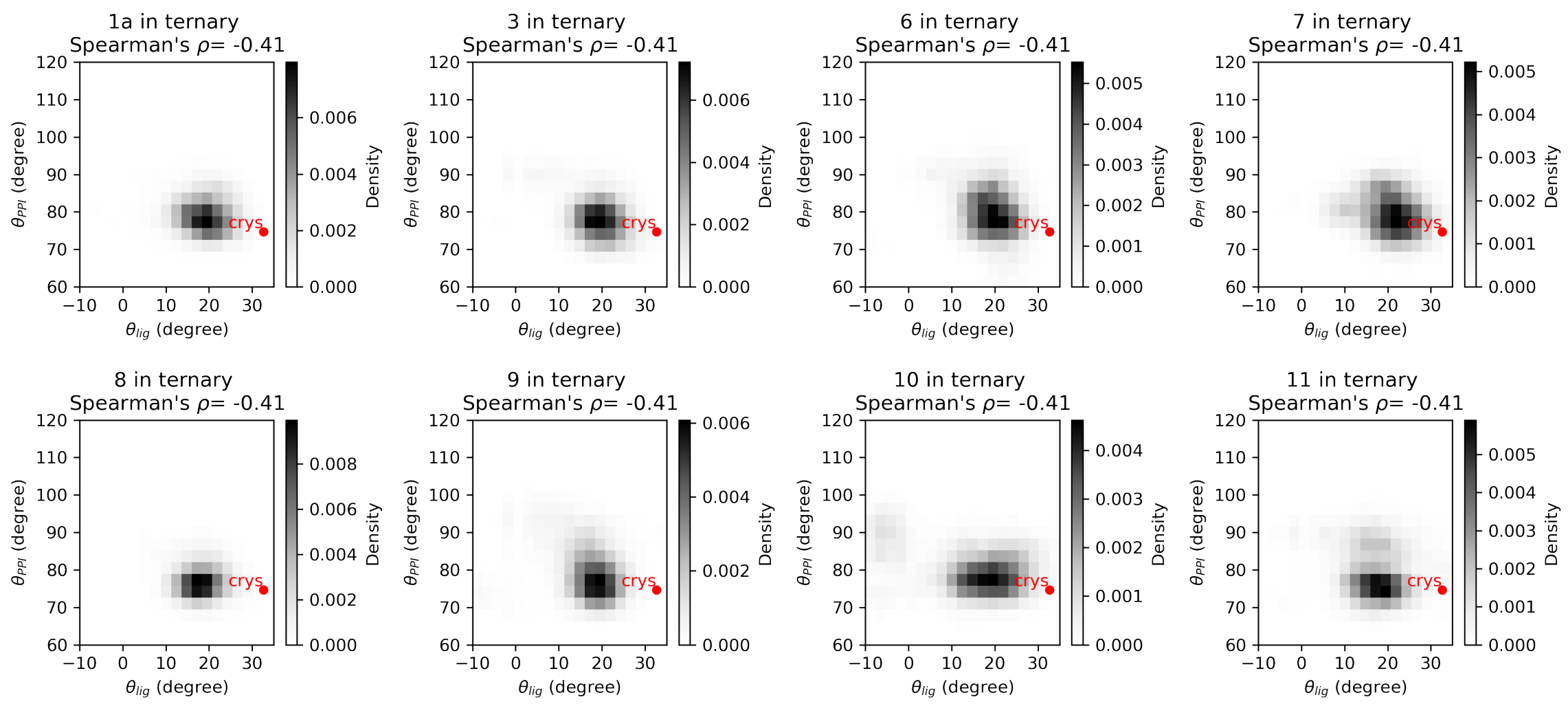

Figure S8: Correlation between the MAPRE1 tilting angle, with respect to FKBP12, and the ligand core-to-core angle observed from the 200 ns REST2 simulations of the eight active compounds (1a, 3, 6, 7, 8, 9, 10, and 11). The red point indicates angles measured from the FKBP12-1a-MAPRE1 crystal structure (PDB ID 9CO5).

S4 Convergence in Conventional MD and REST2 Enhanced Sampling

S4.1 Conventional MD Simulations Show Kinetic Traps

In the conventional MD simulations, compounds **1a**, **8**, **10** and **11** were simulated for 1 μs in binary complexes (FL) and ternary complexes (FLM) for three replicates. With two interfaces per ternary complex, the FLM simulations provided six examples of ligand dynamics, whereas the FL simulations provided three examples of ligand dynamics. We found that the conformations of the ligand can become kinetically trapped in the conventional MD simulations. For example, in the FL simulations of compounds **8**, **10**, and **11**, θ_{lig} was sampled at around 0° or at around $-30°$ for a long period of time before switching to another angle (Figure S9A). A similar kinetic trap was also seen in the FLM simulations (Figure S9B).

The slow transitions between the conformational states make the statistics of the θ_{lig} distribution dependent on the length of the simulation.

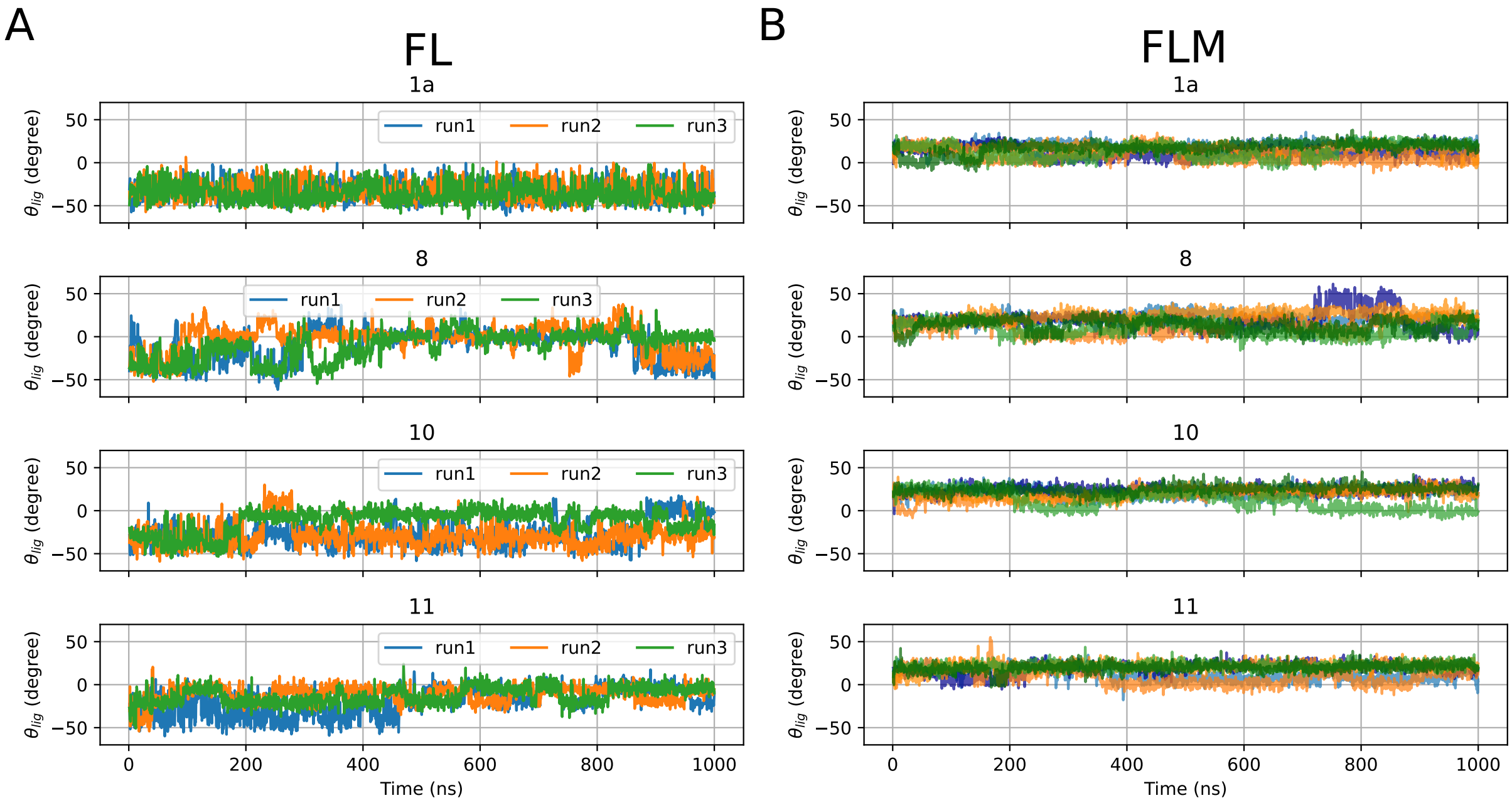

Figure S9: Time evolution of θ_{lig} in the conventional MD simulations of 1 μs with three replicates (blue, orange, and green). Simulations were performed for compounds **1a**, **8**, **10**, and **11** in the FL complex (A) and FLM complex (B). Data from the two interfaces in the FLM complexes from the same simulations are shown in different shades of the same color.

S4.2 Fast θ_{lig} Transitions Observed in REST2 Simulations

To ensure convergence, REST2 simulations were performed for 200 ns each. In the FL simulations, the transitions of θ_{lig} between about $0°$ or at about $-50°$ occurred on the sub-nanosecond timescale (Figure S10A). Although some extreme values (such as $96°$ for compound **3** and $-95°$ for compound **11**) were sampled, they only occupy a small fraction and did not affect overall statistics (Figure S10A). All simulations showed similar distributions of θ_{lig} when comparing the data between 50-150 ns and between 50-200 ns, suggesting decent convergence (Figure S10B).

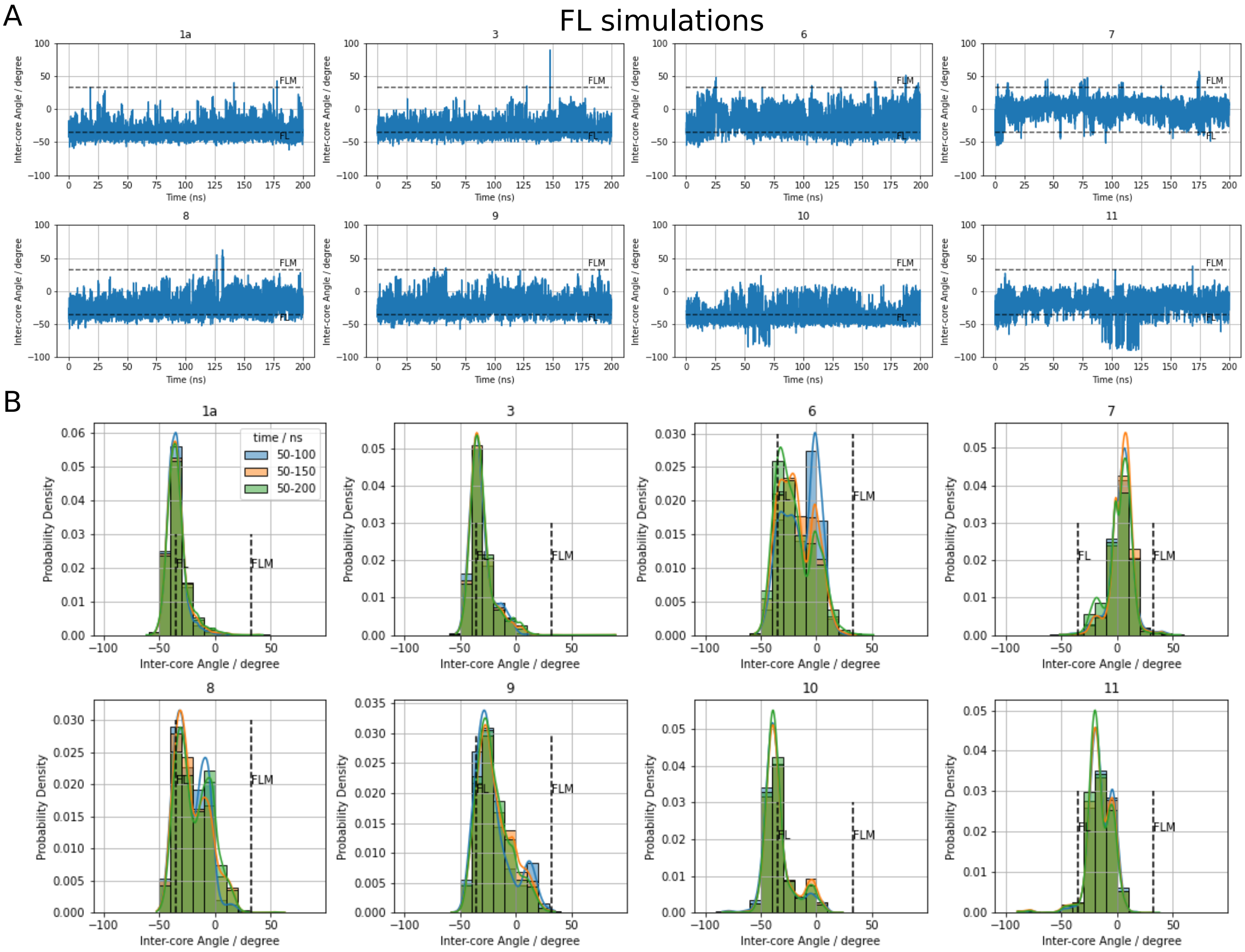

Figure S10: Analysis of the 200 ns REST2 simulations of the FL complexes. (A): Time evolution of θ_{lig} in replica 1. The dashed horizontal lines show the θ_{lig} measured in the crystal structures. (B): Distribution of θ_{lig} for the time window 50-100 ns (blue), 50-150 ns (orange), and 50-200 ns (green). The dashed vertical lines show the θ_{lig} measured in the crystal structures.

Similarly, a fast transition between 0° and 50° was observed in FLM complex simulations. Again, θ_{lig} distributions were similar for 50-150 ns and 50-200 ns (Figure S11).

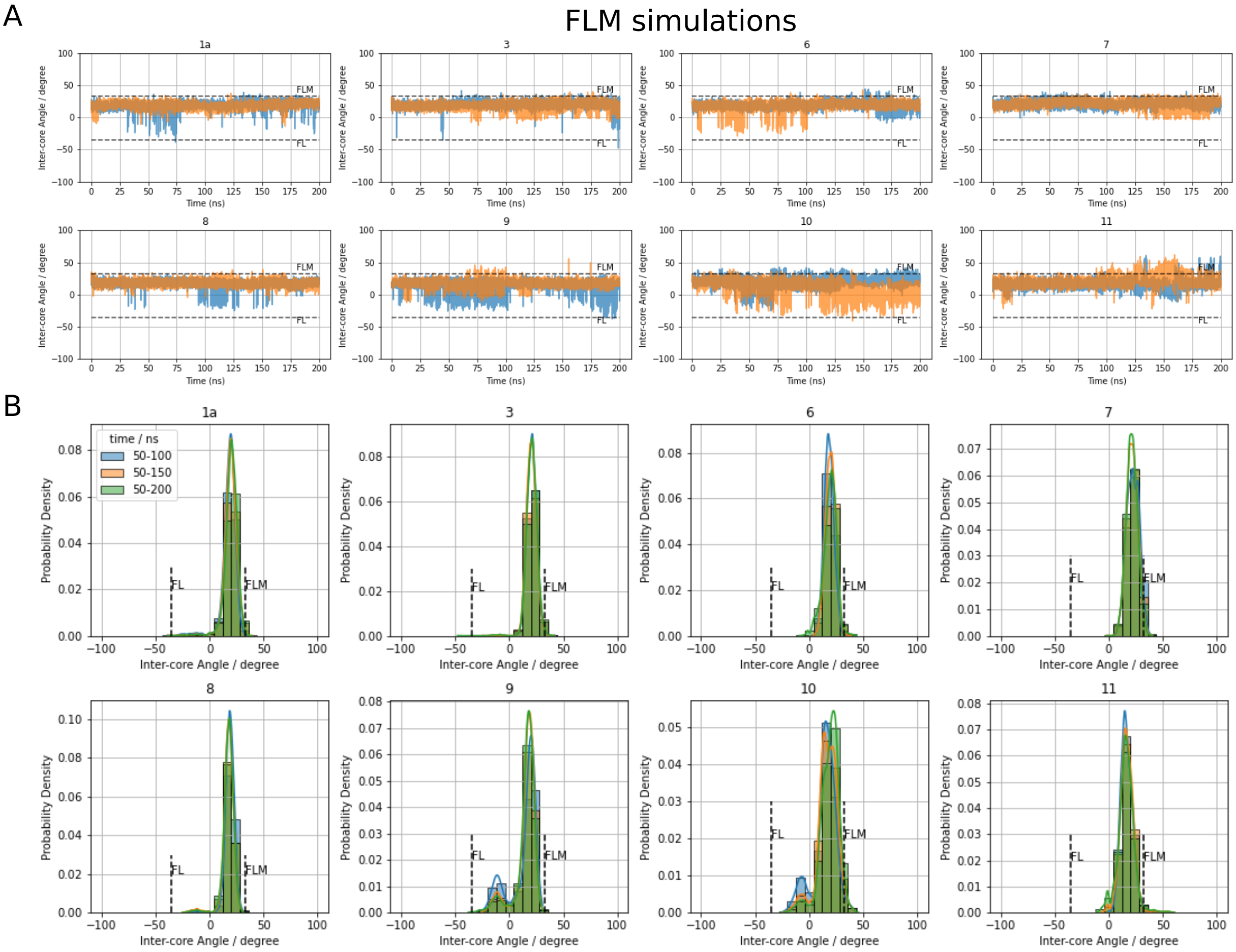

Figure S11: Analysis of the 200 ns REST2 simulations of the FLM complexes. (A): Time evolution of θ_{lig} in replica 1. The values of the two interfaces are shown in blue and orange. The dashed horizontal lines show the θ_{lig} measured in the crystal structures. (B): Distribution of θ_{lig} for the time window 50-100 ns (blue), 50-150 ns (orange), and 50-200 ns (green). The dashed vertical lines show the θ_{lig} measured in the crystal structures.

In the REST2 simulations of the ligand free in solution, fast transitions between θ_{lig} values were observed across the full range of angles observed in the FL and FLM complexes (Figure S12A), and the statistics suggest convergence of the distribution θ_{lig} after 200 ns.

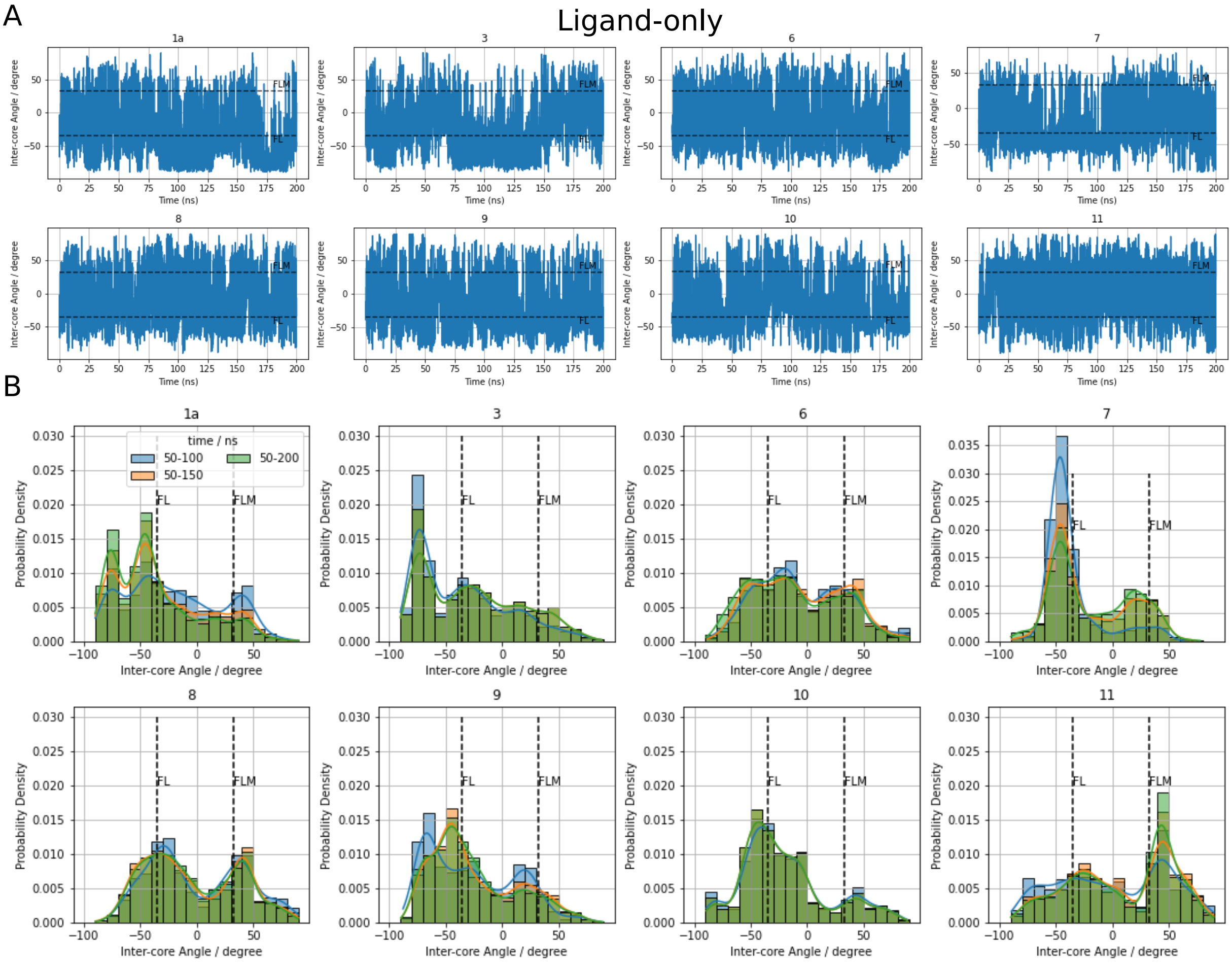

Figure S12: Analysis of the 200 ns REST2 simulations of the ligands in solution. (A): Time evolution of θ_{lig} in replica 1. (B): Distribution of θ_{lig} for the time window 50-100 ns (blue), 50-150 ns (orange), and 50-200 ns (green).

S4.3 Additional Convergence Check of the REST2 Simulations

Besides the observable θ_{lig}, the convergence of the 22 backbone dihedral angles from the eight compounds were examined in the protein-free form (Figures S13-S20), binary complex (Figures S21-S28), and ternary complex (Figures S29-S36). The acceptance ratio between the neighboring replicas in the REST2 simulations is shown in Figure S37.

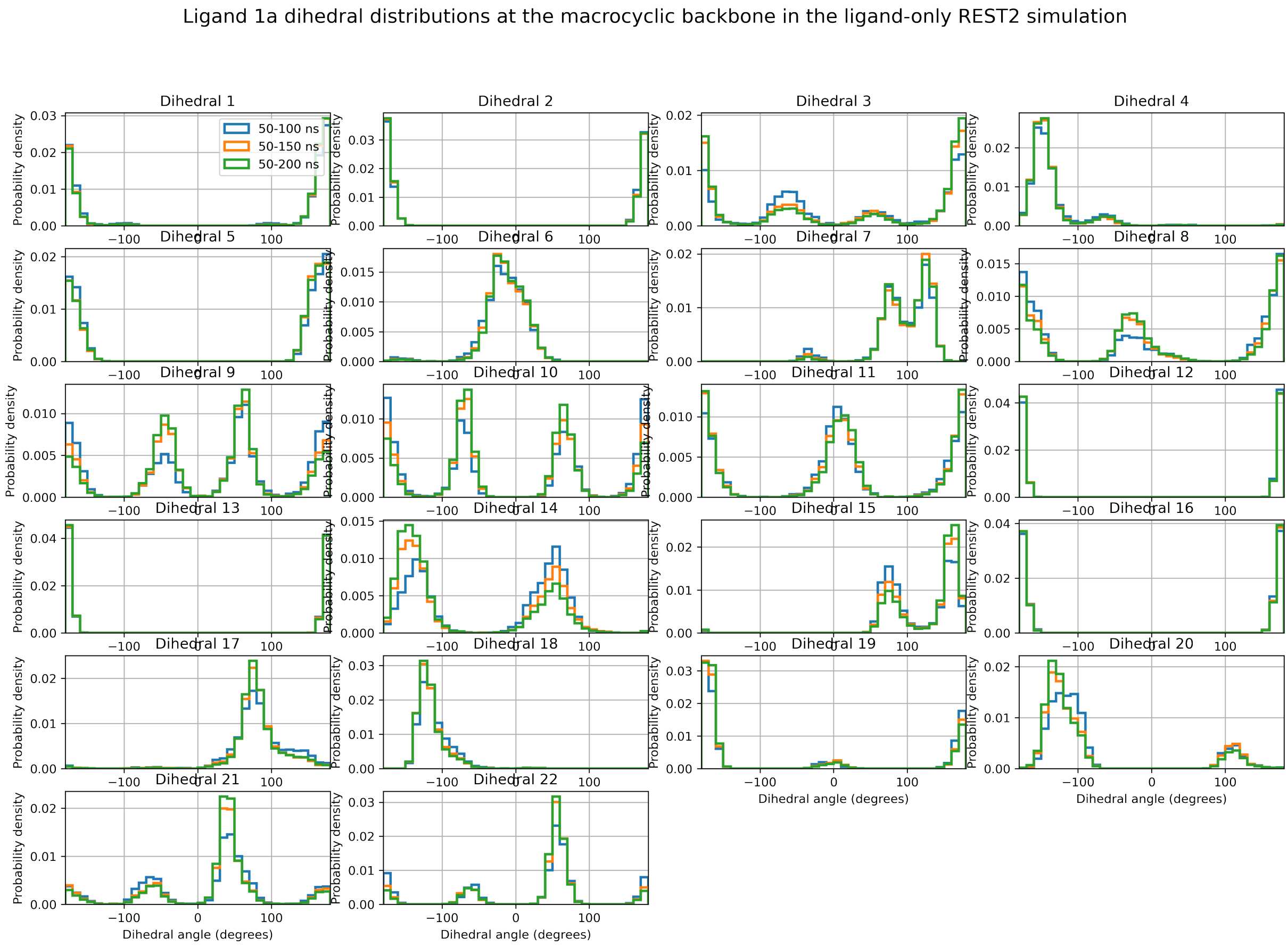

Figure S13: Convergence analysis of the 200 ns REST2 simulations of compound 1a on the backbone dihedral angle in the protein-free form by distribution for the time window 50-100 ns (blue), 50-150 ns (orange), and 50-200 ns (green).

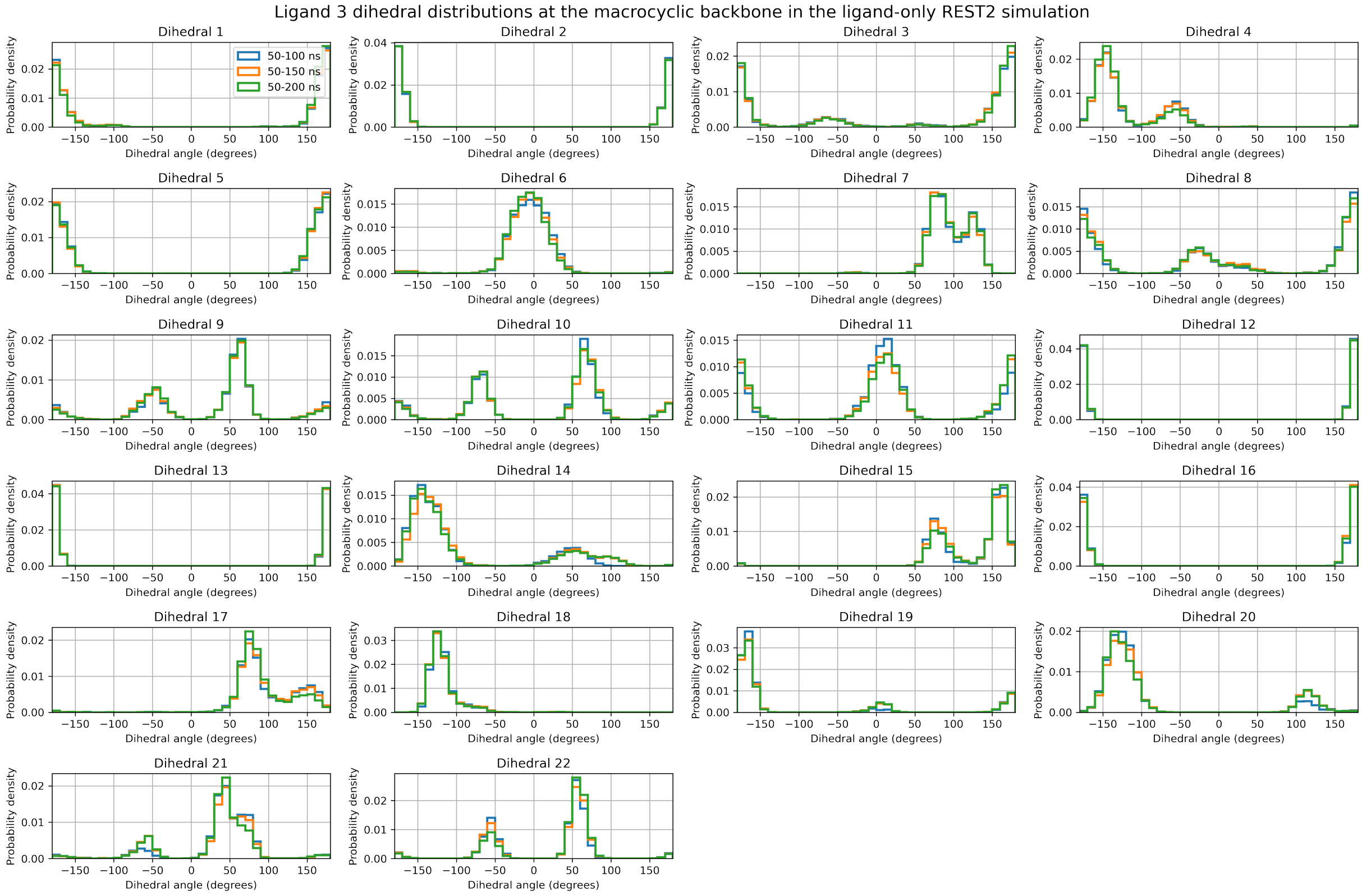

Figure S14: Convergence analysis of the 200 ns REST2 simulations of compound 3 on the backbone dihedral angle in the protein-free form by distribution for the time window 50-100 ns (blue), 50-150 ns (orange), and 50-200 ns (green).

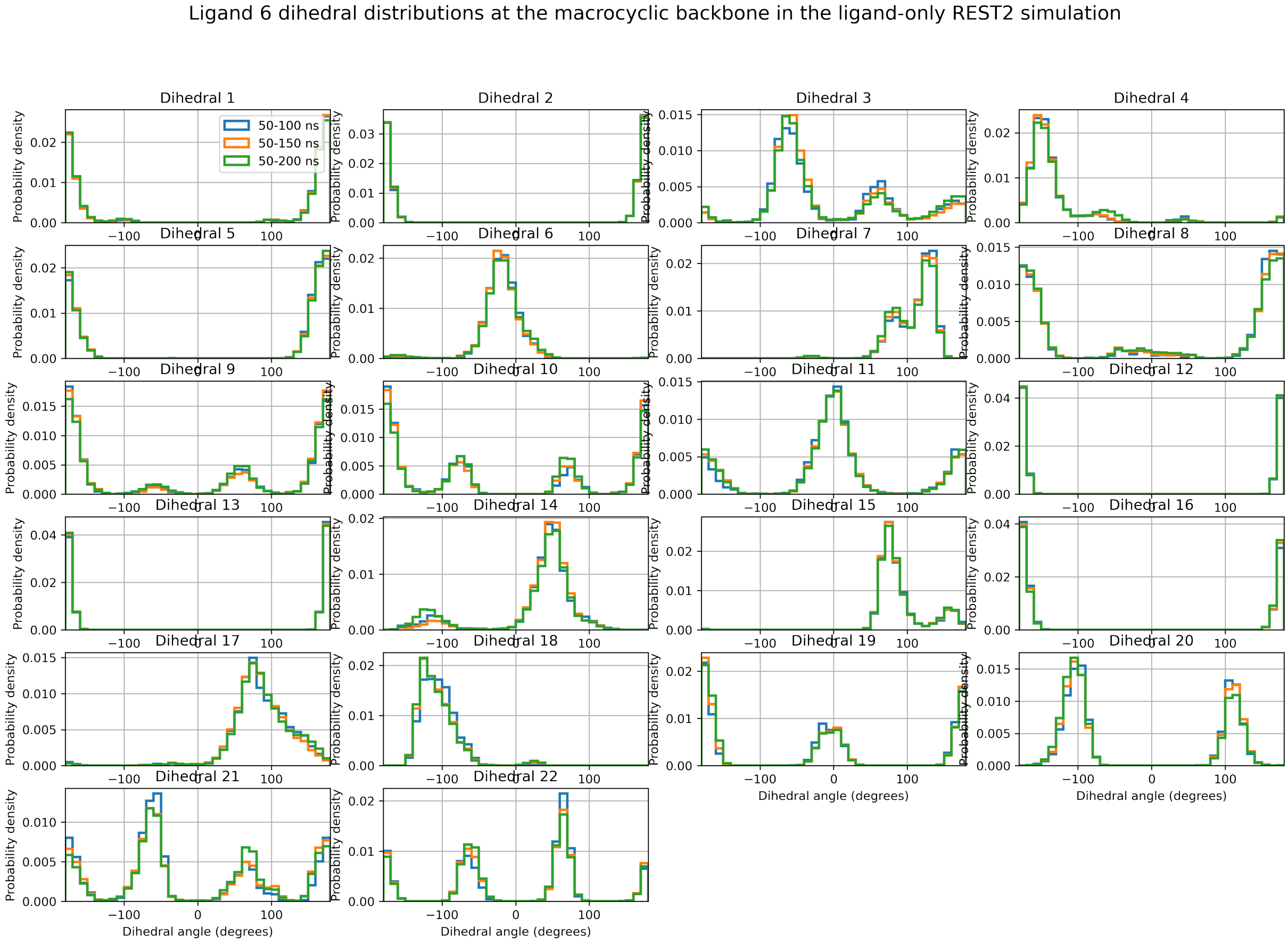

Figure S15: Convergence analysis of the 200 ns REST2 simulations of compound 6 on the backbone dihedral angle in the protein-free form by distribution for the time window 50-100 ns (blue), 50-150 ns (orange), and 50-200 ns (green).

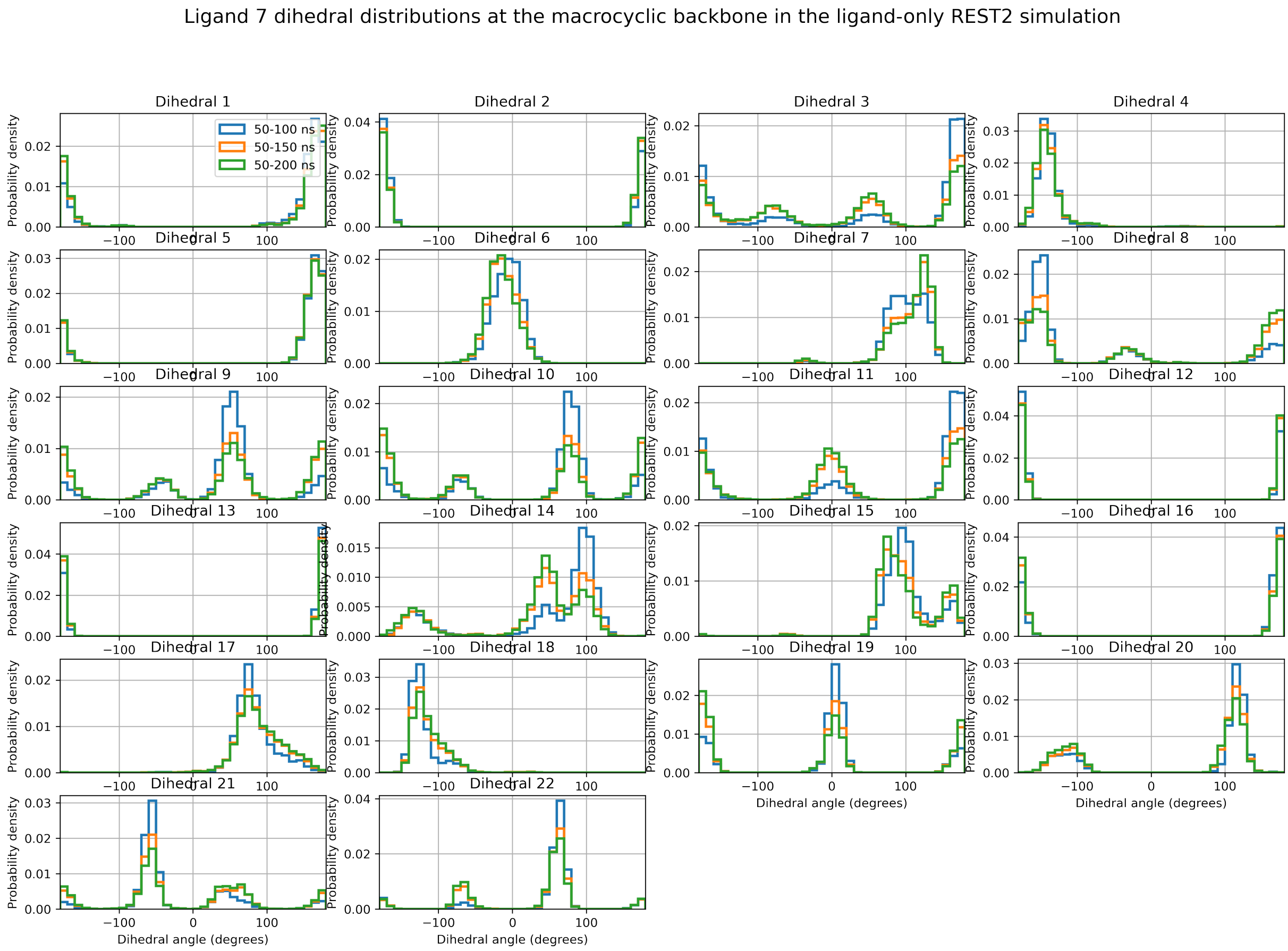

Figure S16: Convergence analysis of the 200 ns REST2 simulations of compound 7 on the backbone dihedral angle in the protein-free form by distribution for the time window 50-100 ns (blue), 50-150 ns (orange), and 50-200 ns (green).

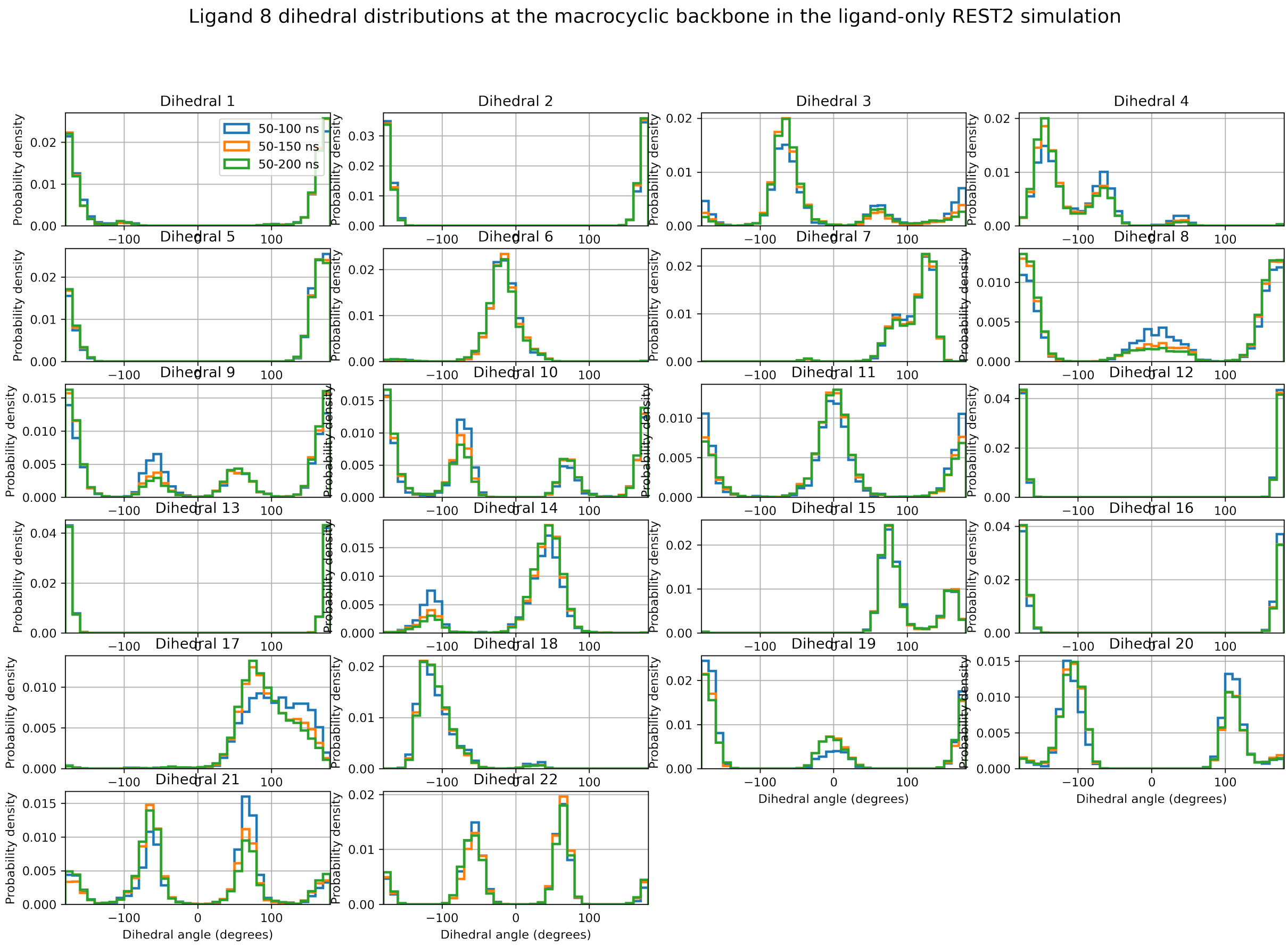

Figure S17: Convergence analysis of the 200 ns REST2 simulations of compound 8 on the backbone dihedral angle in the protein-free form by distribution for the time window 50-100 ns (blue), 50-150 ns (orange), and 50-200 ns (green).

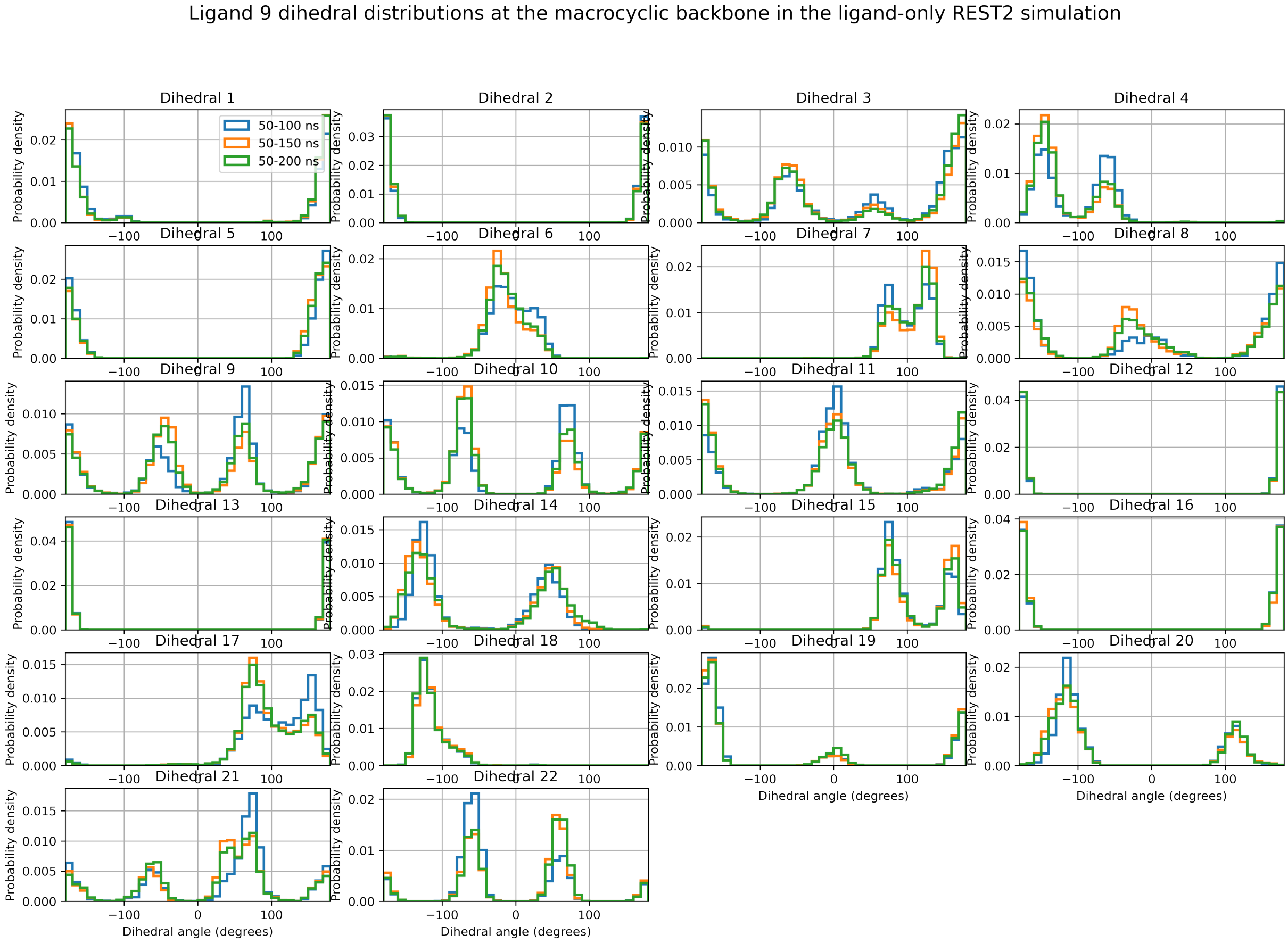

Figure S18: Convergence analysis of the 200 ns REST2 simulations of compound 9 on the backbone dihedral angle in the protein-free form by distribution for the time window 50-100 ns (blue), 50-150 ns (orange), and 50-200 ns (green).

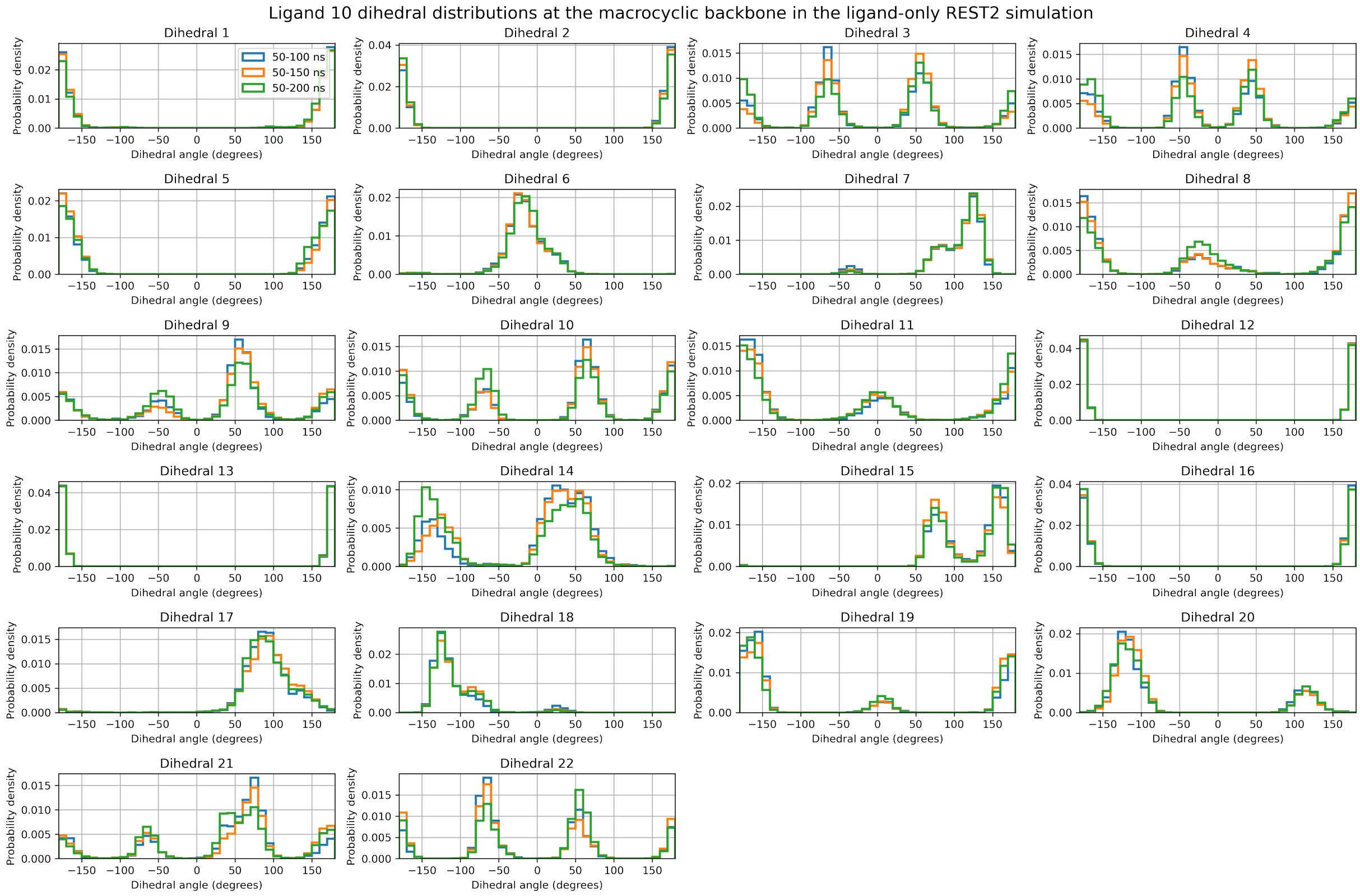

Figure S19: Convergence analysis of the 200 ns REST2 simulations of compound 10 on the backbone dihedral angle in the protein-free form by distribution for the time window 50-100 ns (blue), 50-150 ns (orange), and 50-200 ns (green).

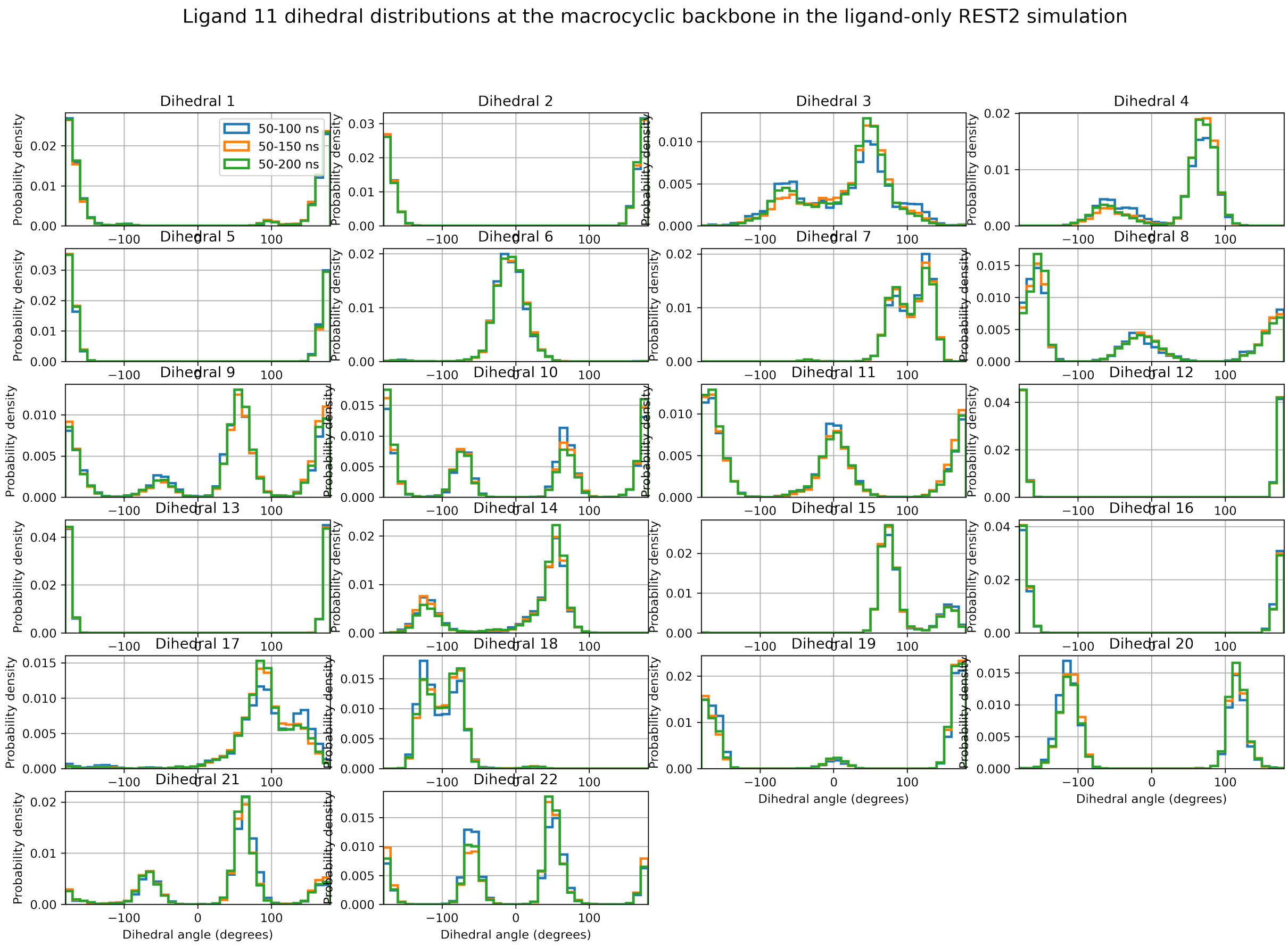

Figure S20: Convergence analysis of the 200 ns REST2 simulations of compound 11 on the backbone dihedral angle in the protein-free form by distribution for the time window 50-100 ns (blue), 50-150 ns (orange), and 50-200 ns (green).

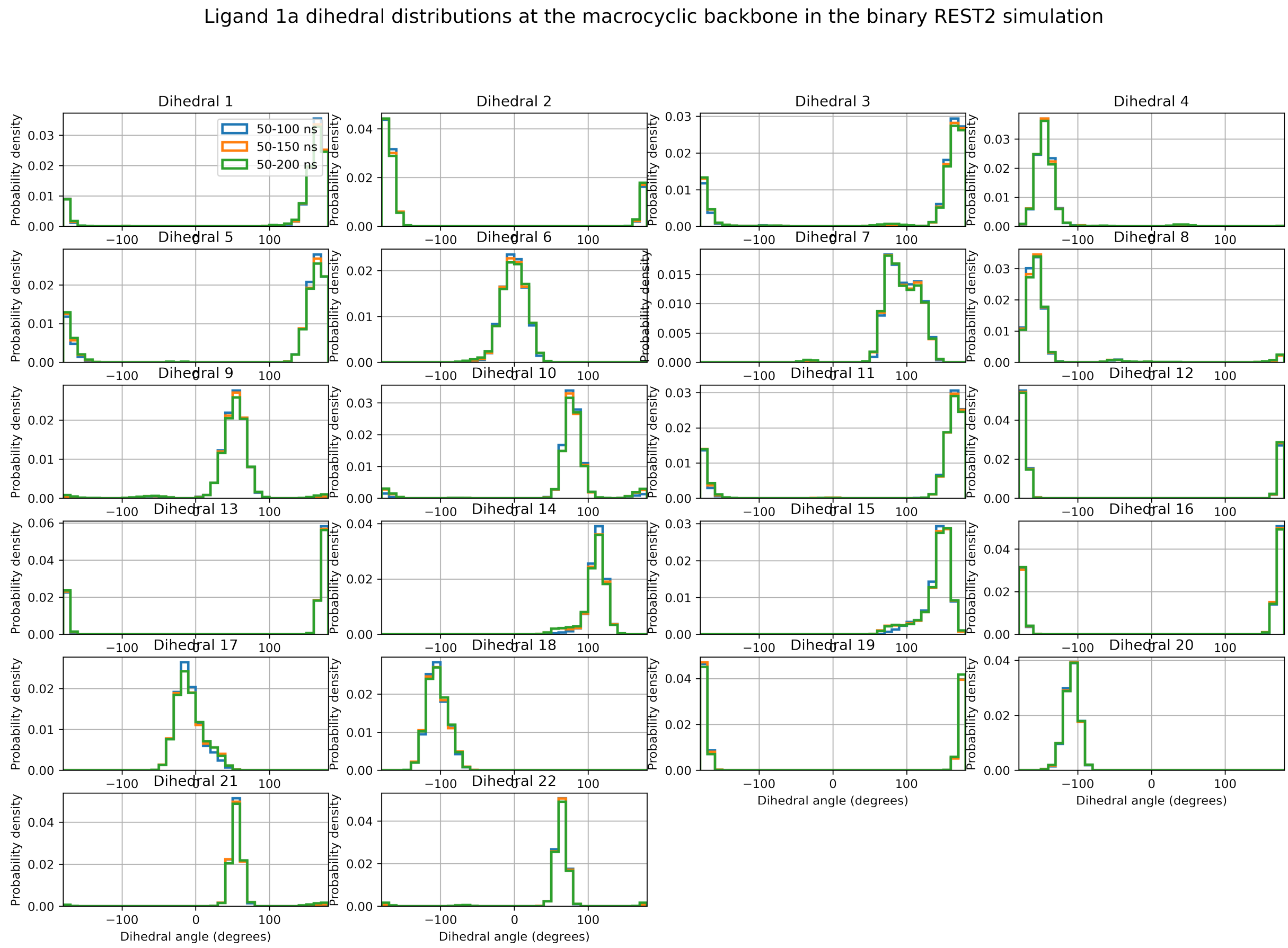

Figure S21: Convergence analysis of the 200 ns REST2 simulations of compound 1a on the backbone dihedral angle in the binary complex by distribution for the time window 50-100 ns (blue), 50-150 ns (orange), and 50-200 ns (green).

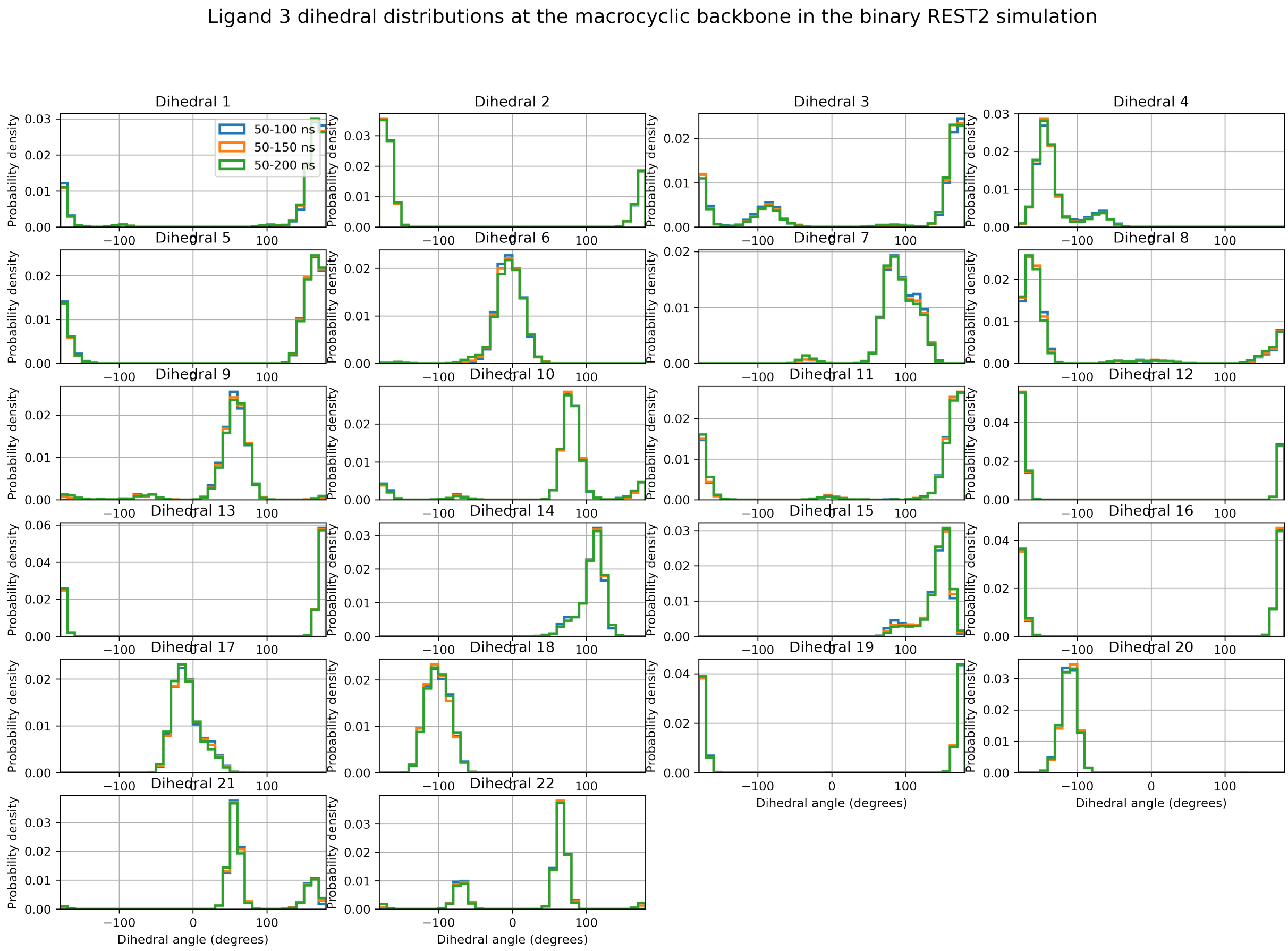

Figure S22: Convergence analysis of the 200 ns REST2 simulations of the ligand 3 on the backbone dihedral angle in the binary complex by distribution for the time window 50-100 ns (blue), 50-150 ns (orange), and 50-200 ns (green).

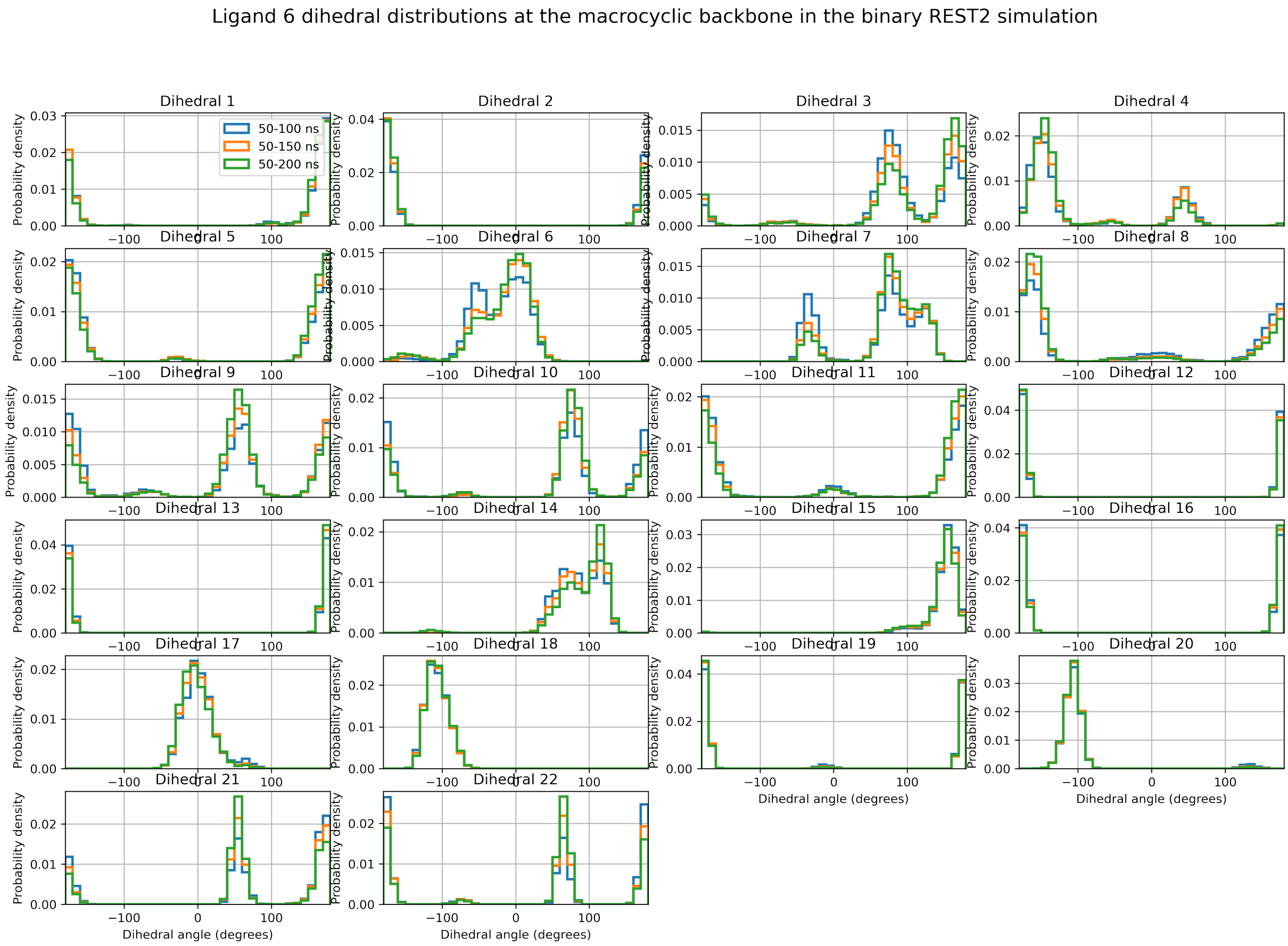

Figure S23: Convergence analysis of the 200 ns REST2 simulations of compound 6 on the backbone dihedral angle in the binary complex by distribution for the time window 50-100 ns (blue), 50-150 ns (orange), and 50-200 ns (green).

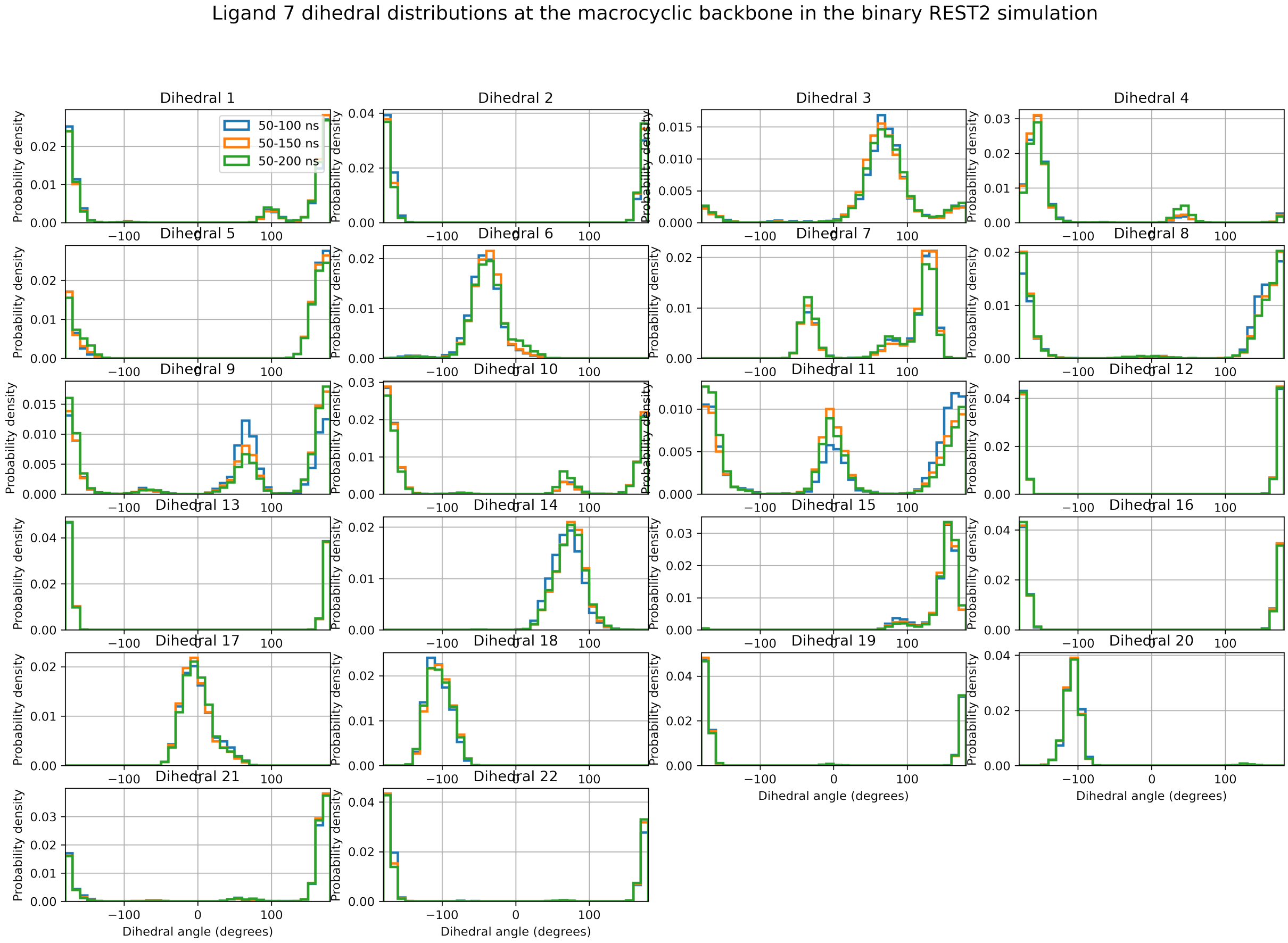

Figure S24: Convergence analysis of the 200 ns REST2 simulations of compound 7 on the backbone dihedral angle in the binary complex by distribution for the time window 50-100 ns (blue), 50-150 ns (orange), and 50-200 ns (green).

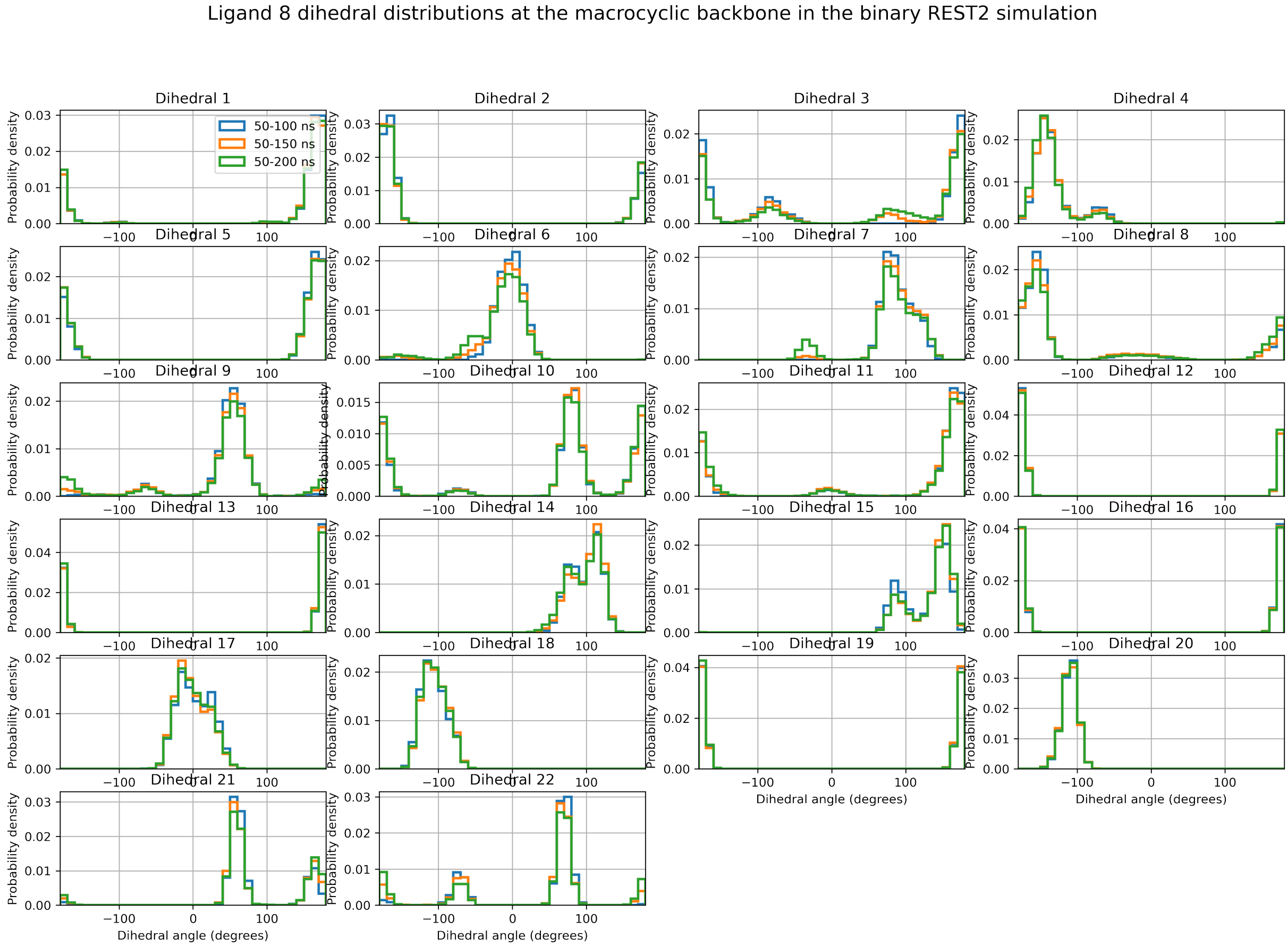

Figure S25: Convergence analysis of the 200 ns REST2 simulations of compound 8 on the backbone dihedral angle in the binary complex by distribution for the time window 50-100 ns (blue), 50-150 ns (orange), and 50-200 ns (green).

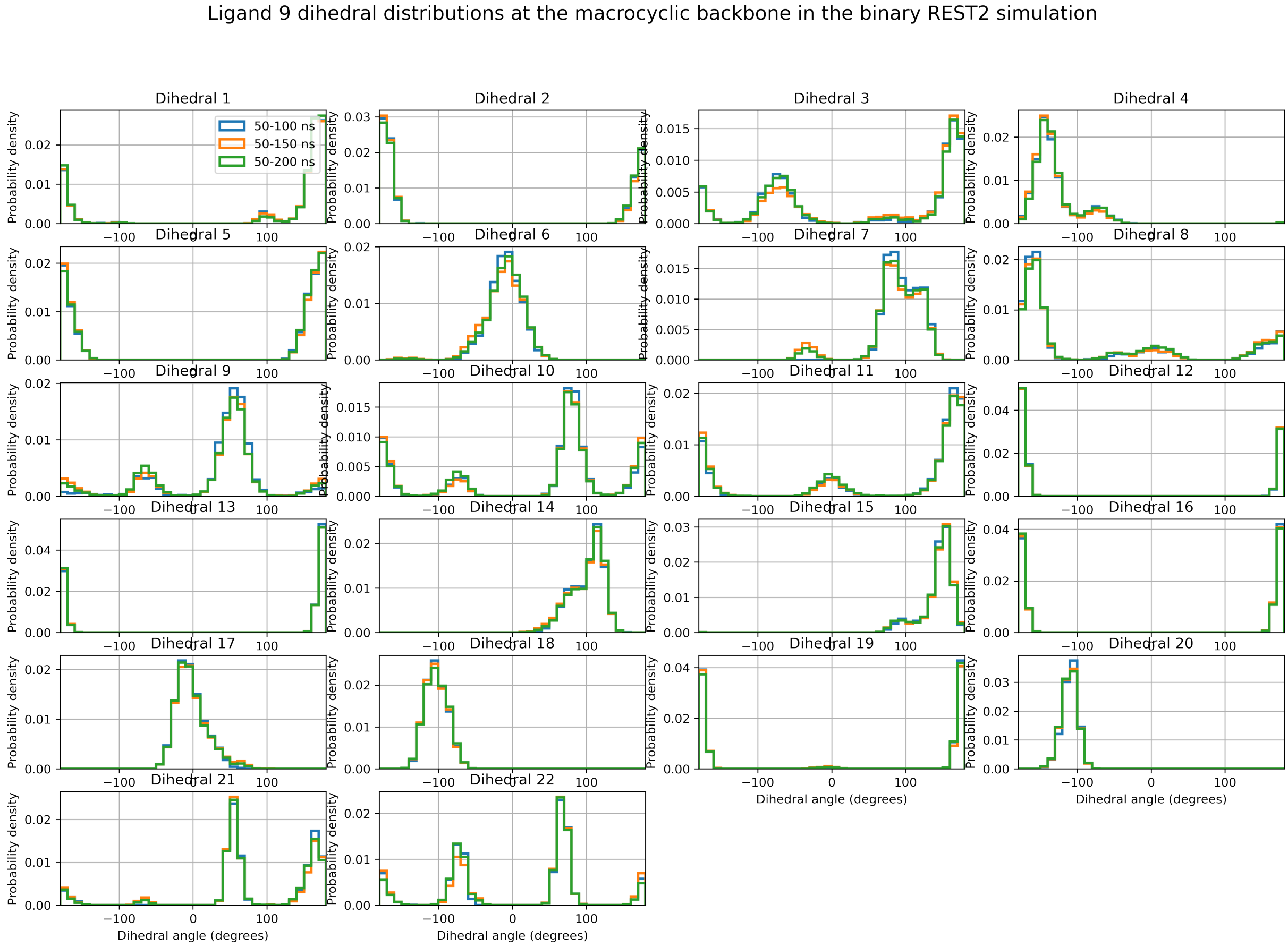

Figure S26: Convergence analysis of the 200 ns REST2 simulations of compound 9 on the backbone dihedral angle in the binary complex by distribution for the time window 50-100 ns (blue), 50-150 ns (orange), and 50-200 ns (green).

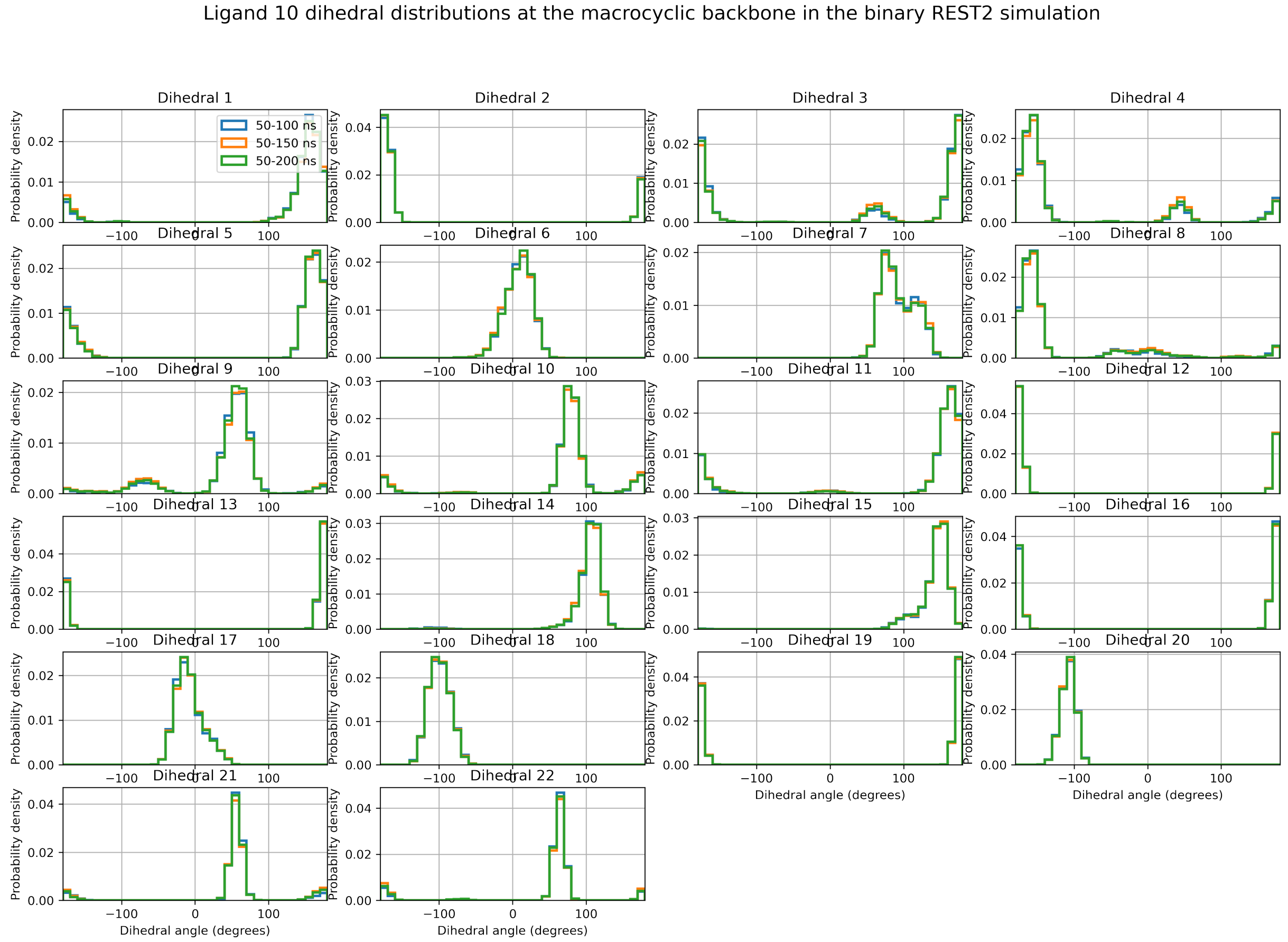

Figure S27: Convergence analysis of the 200 ns REST2 simulations of compound 10 on the backbone dihedral angle in the binary complex by distribution for the time window 50-100 ns (blue), 50-150 ns (orange), and 50-200 ns (green).

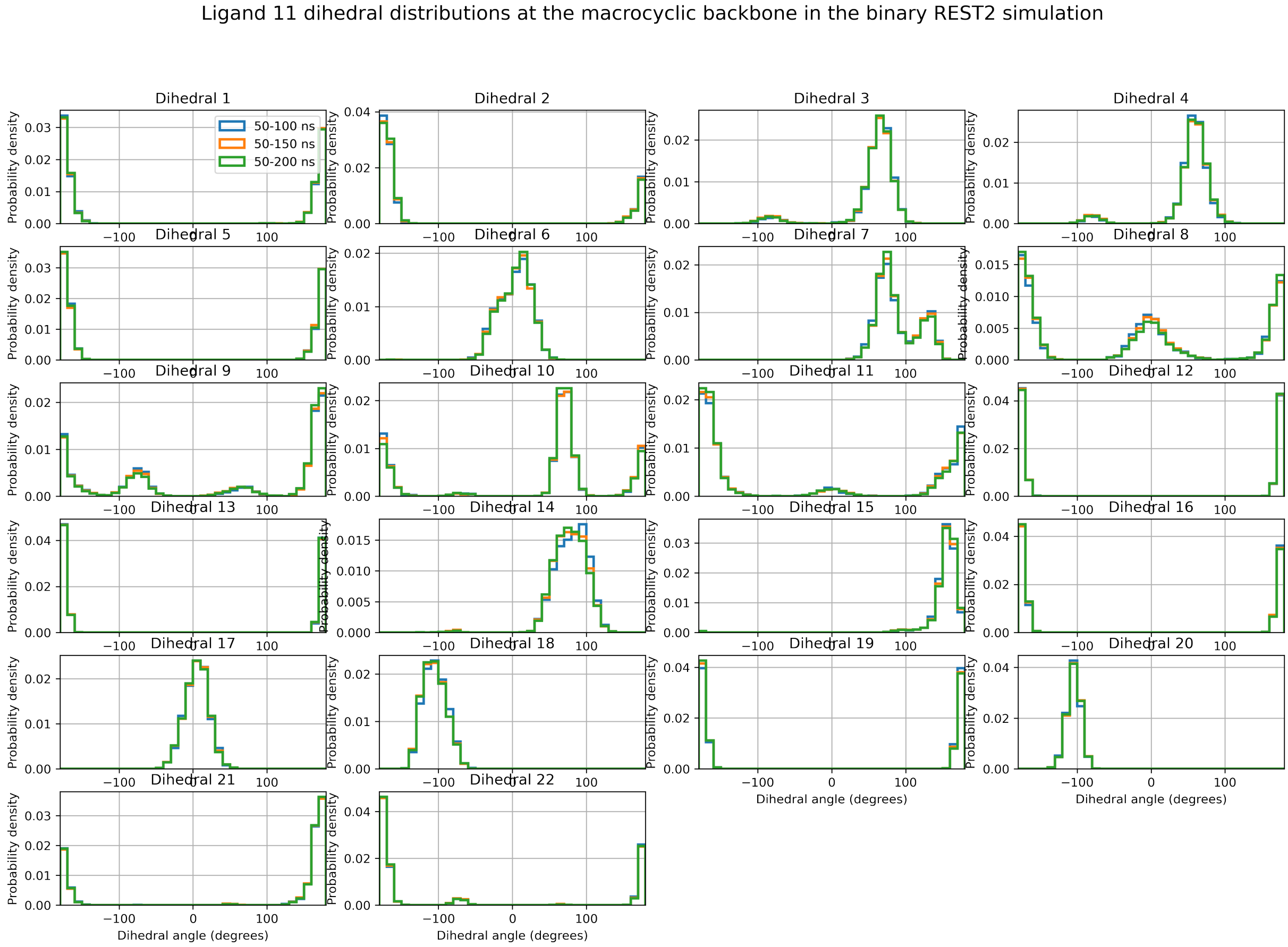

Figure S28: Convergence analysis of the 200 ns REST2 simulations of compound 11 on the backbone dihedral angle in the binary complex by distribution for the time window 50-100 ns (blue), 50-150 ns (orange), and 50-200 ns (green).

Ligand 1a dihedral distributions at the macrocyclic backbone in the ternary REST2 simulation

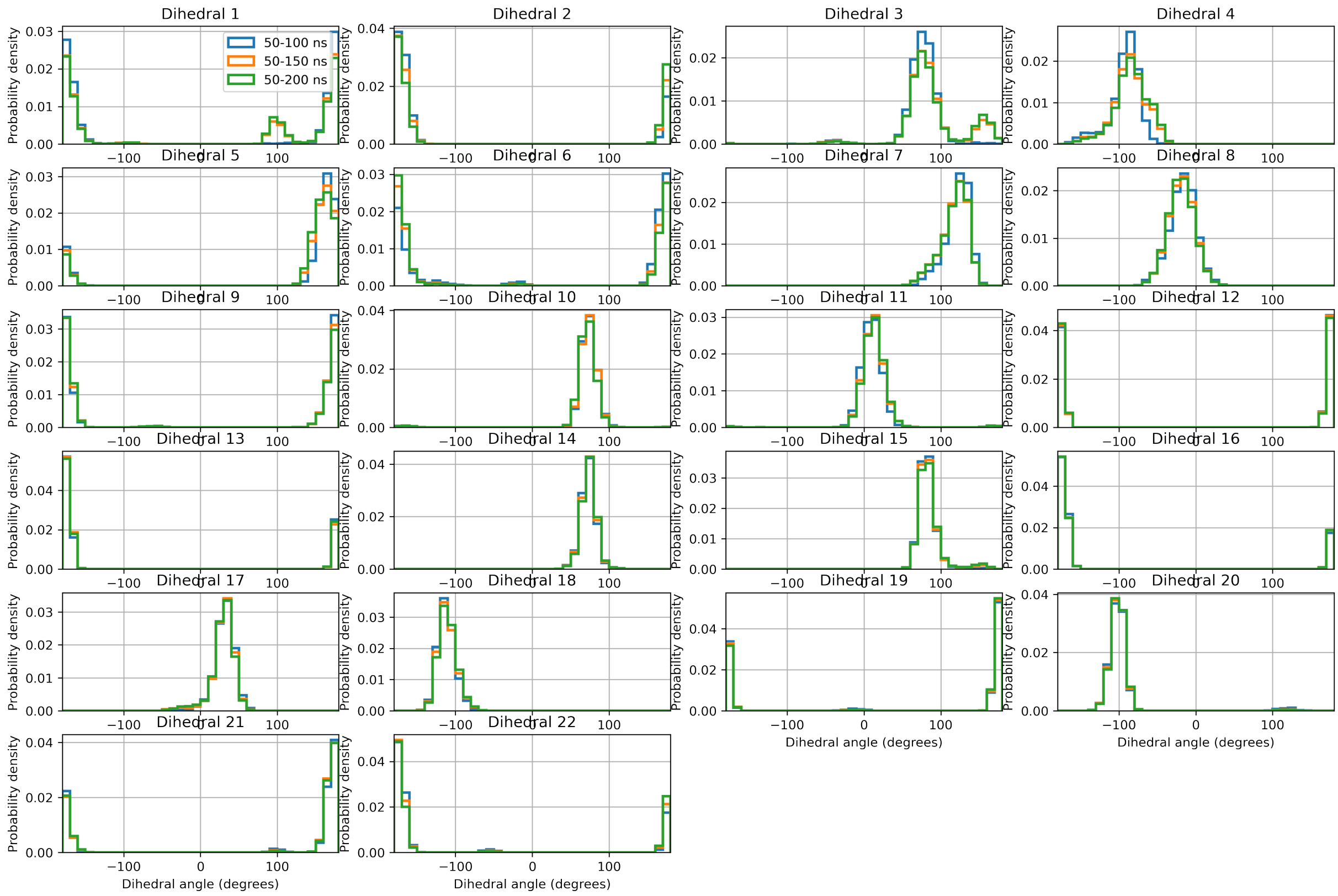

Figure S29: Convergence analysis of the 200 ns REST2 simulations of compound 1a on the backbone dihedral angle in the ternary complex by distribution for the time window 50-100 ns (blue), 50-150 ns (orange), and 50-200 ns (green).

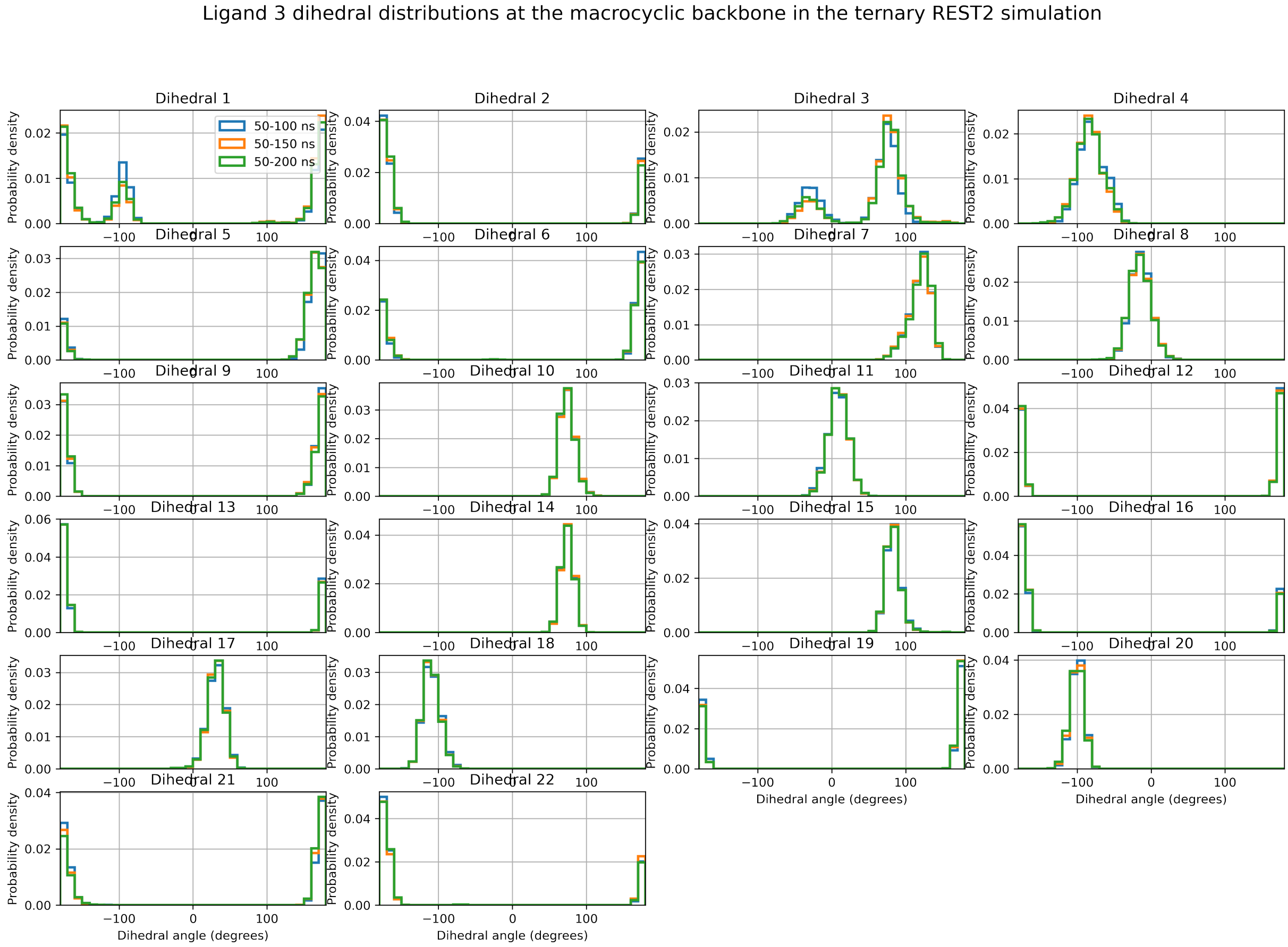

Figure S30: Convergence analysis of the 200 ns REST2 simulations of compound 3 on the backbone dihedral angle in the ternary complex by distribution for the time window 50-100 ns (blue), 50-150 ns (orange), and 50-200 ns (green).

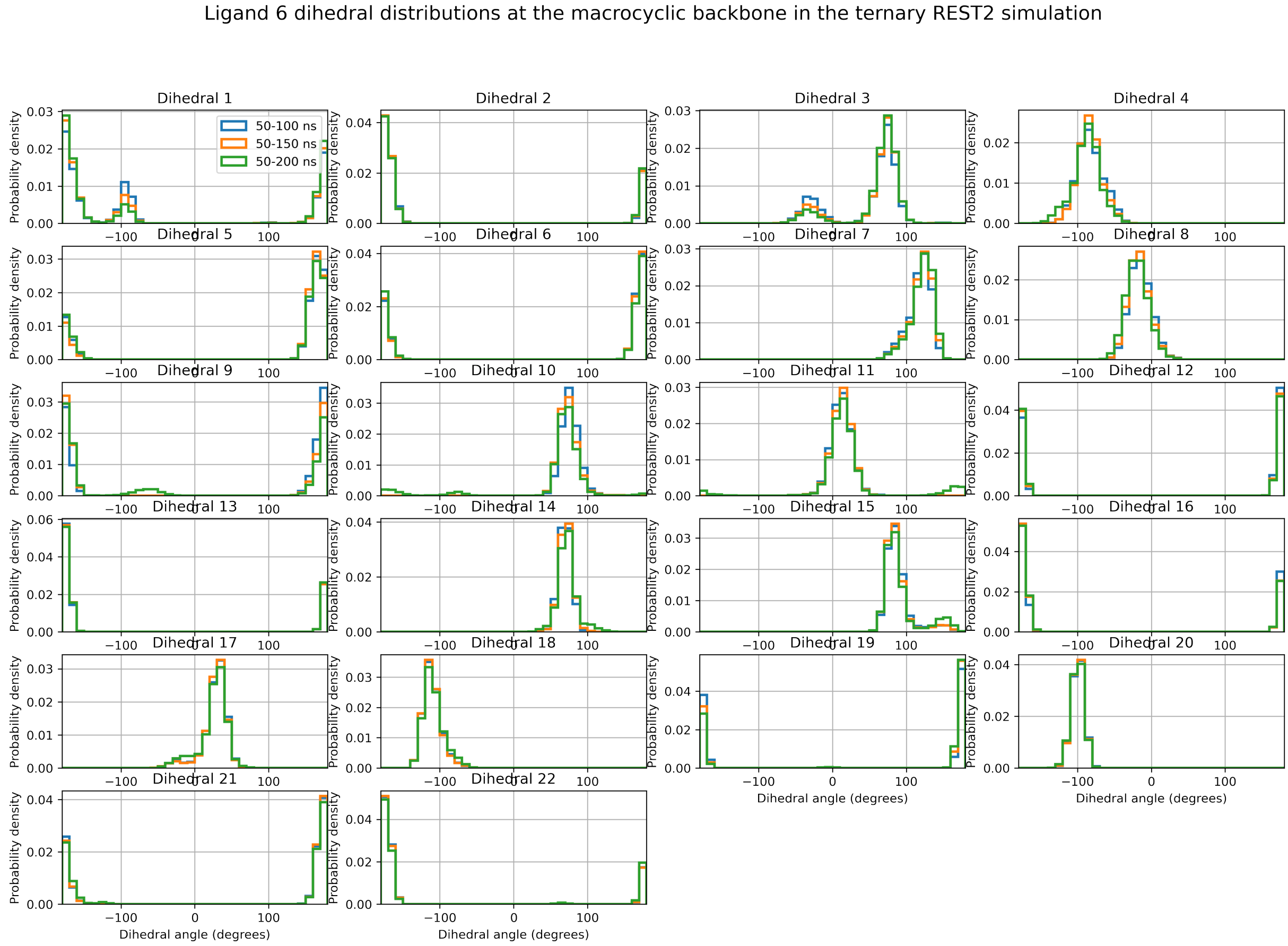

Figure S31: Convergence analysis of the 200 ns REST2 simulations of compound 6 on the backbone dihedral angle in the ternary complex by distribution for the time window 50-100 ns (blue), 50-150 ns (orange), and 50-200 ns (green).

Ligand 7 dihedral distributions at the macrocyclic backbone in the ternary REST2 simulation

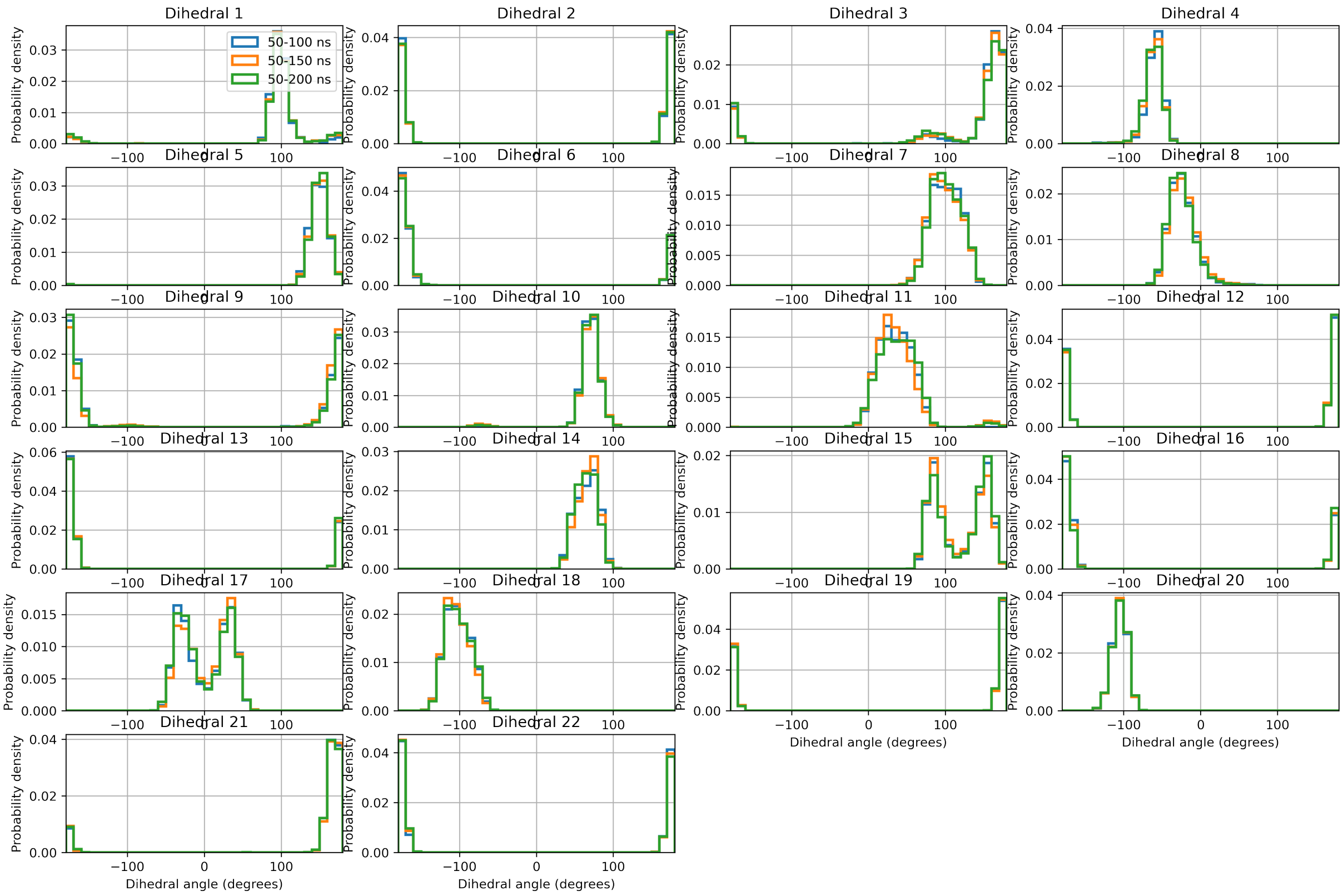

Figure S32: Convergence analysis of the 200 ns REST2 simulations of compound 7 on the backbone dihedral angle in the ternary complex by distribution for the time window 50-100 ns (blue), 50-150 ns (orange), and 50-200 ns (green).

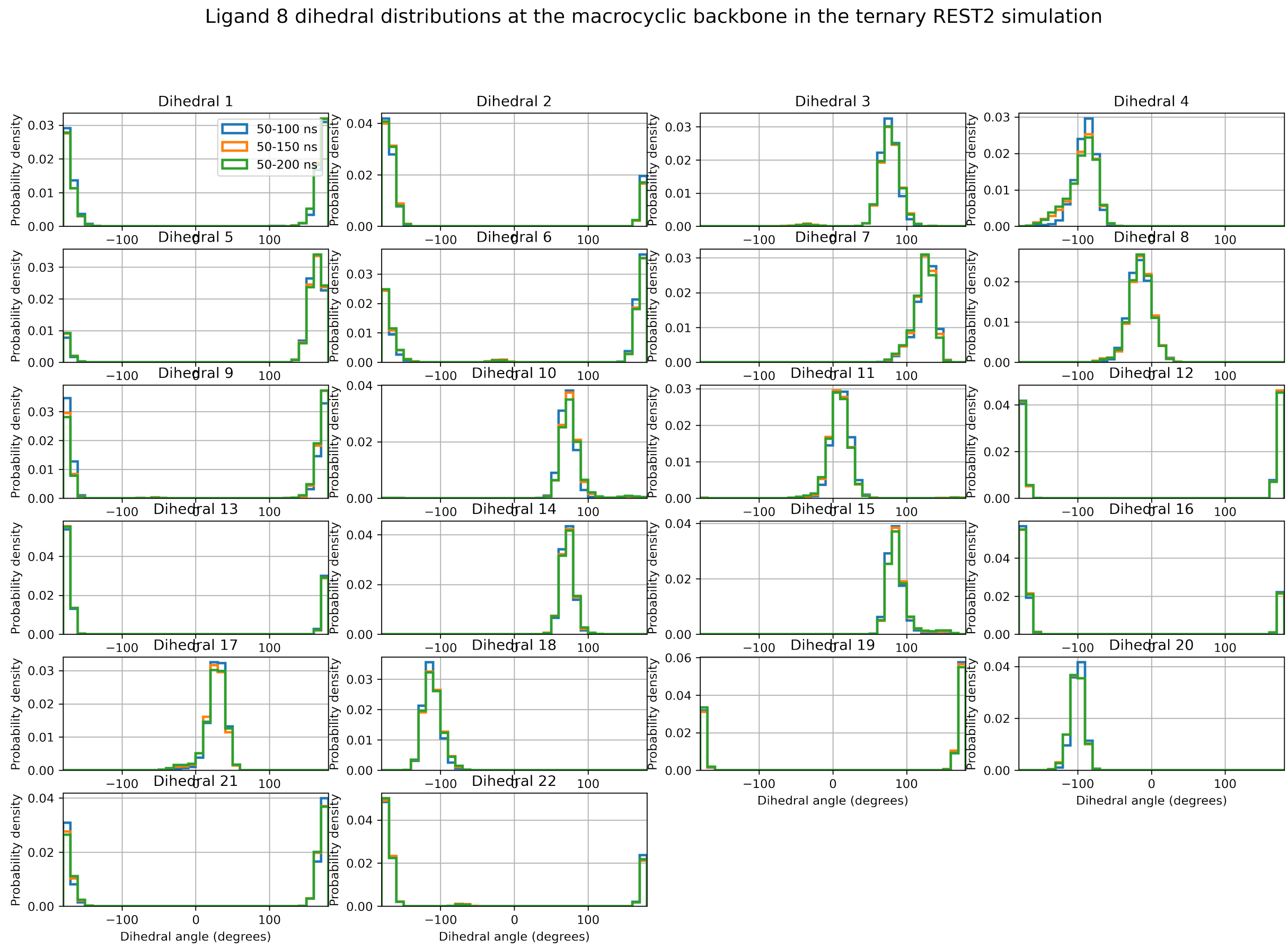

Figure S33: Convergence analysis of the 200 ns REST2 simulations of compound 8 on the backbone dihedral angle in the ternary complex by distribution for the time window 50-100 ns (blue), 50-150 ns (orange), and 50-200 ns (green).

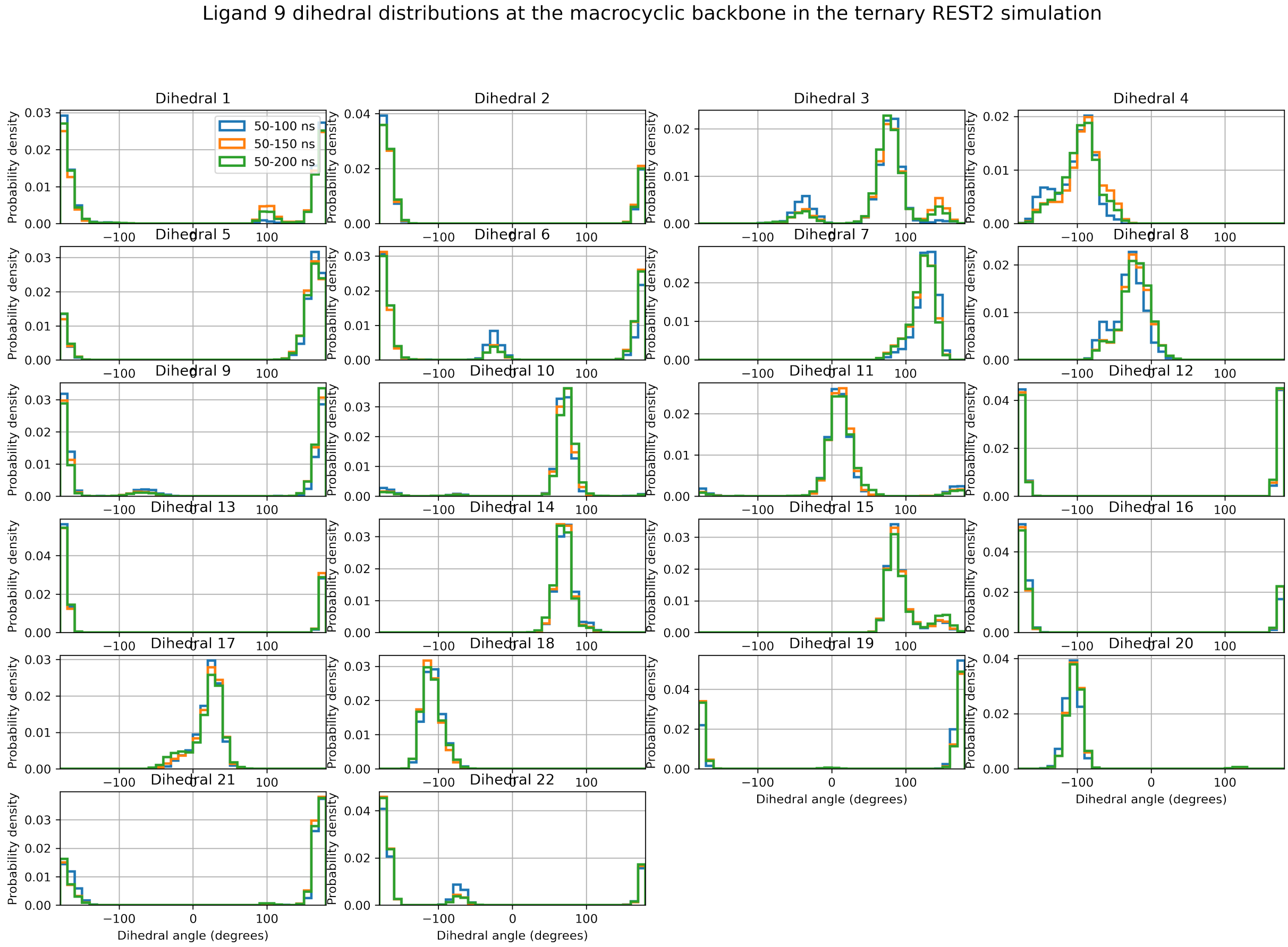

Figure S34: Convergence analysis of the 200 ns REST2 simulations of compound 9 on the backbone dihedral angle in the ternary complex by distribution for the time window 50-100 ns (blue), 50-150 ns (orange), and 50-200 ns (green).

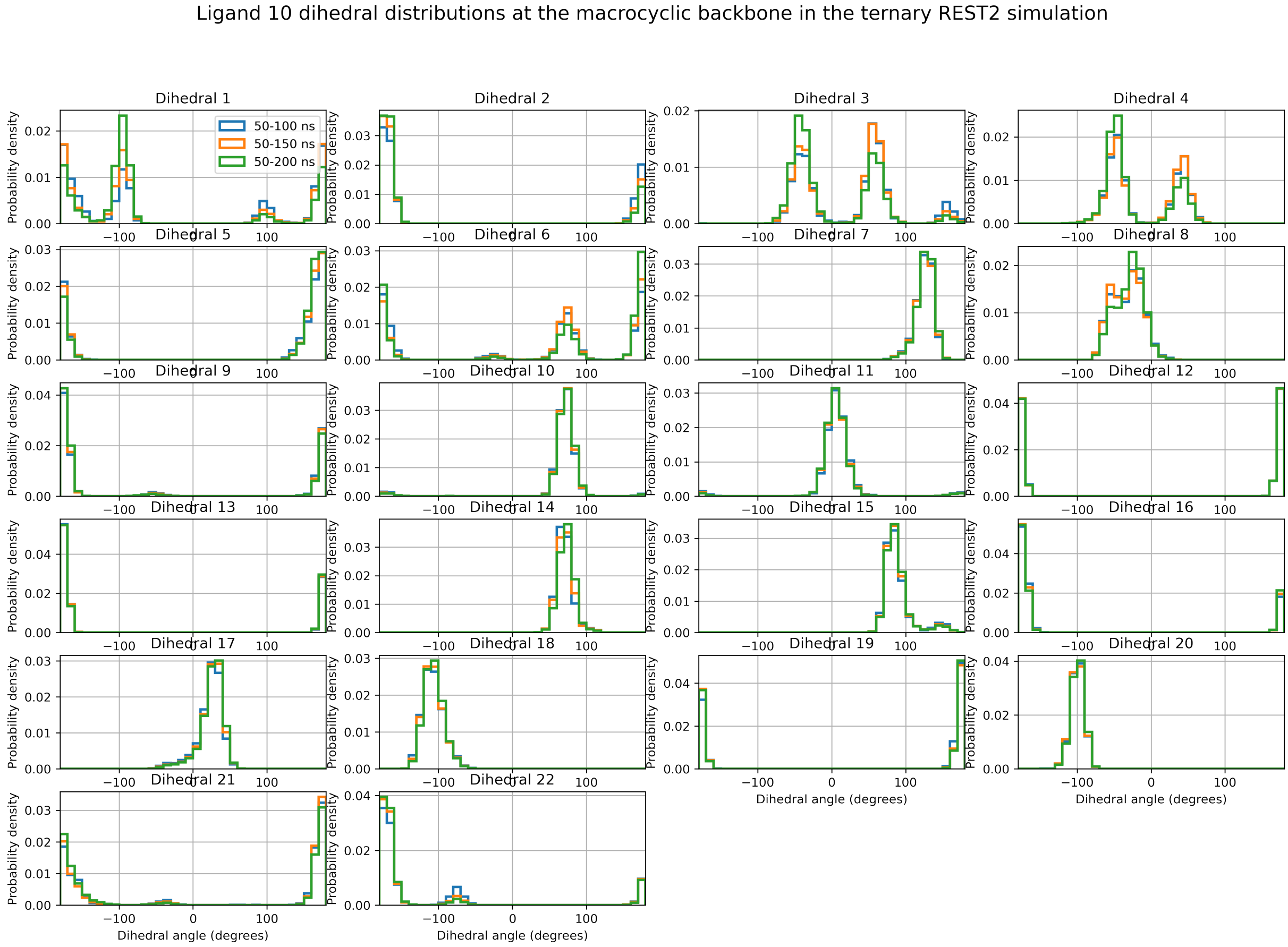

Figure S35: Convergence analysis of the 200 ns REST2 simulations of compound 10 on the backbone dihedral angle in the ternary complex by distribution for the time window 50-100 ns (blue), 50-150 ns (orange), and 50-200 ns (green).

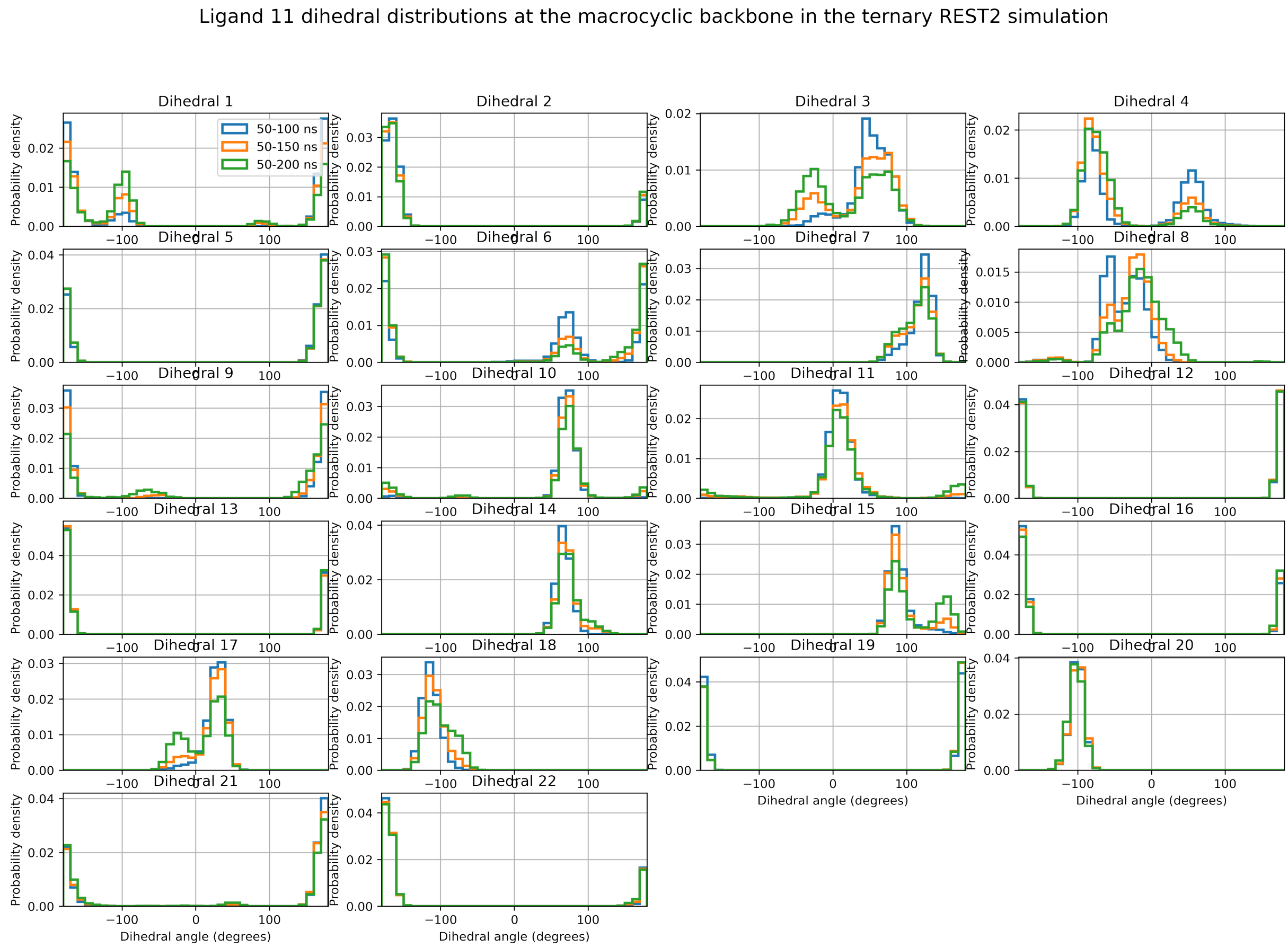

Figure S36: Convergence analysis of the 200 ns REST2 simulations of compound 11 on the backbone dihedral angle in the ternary complex by distribution for the time window 50-100 ns (blue), 50-150 ns (orange), and 50-200 ns (green).

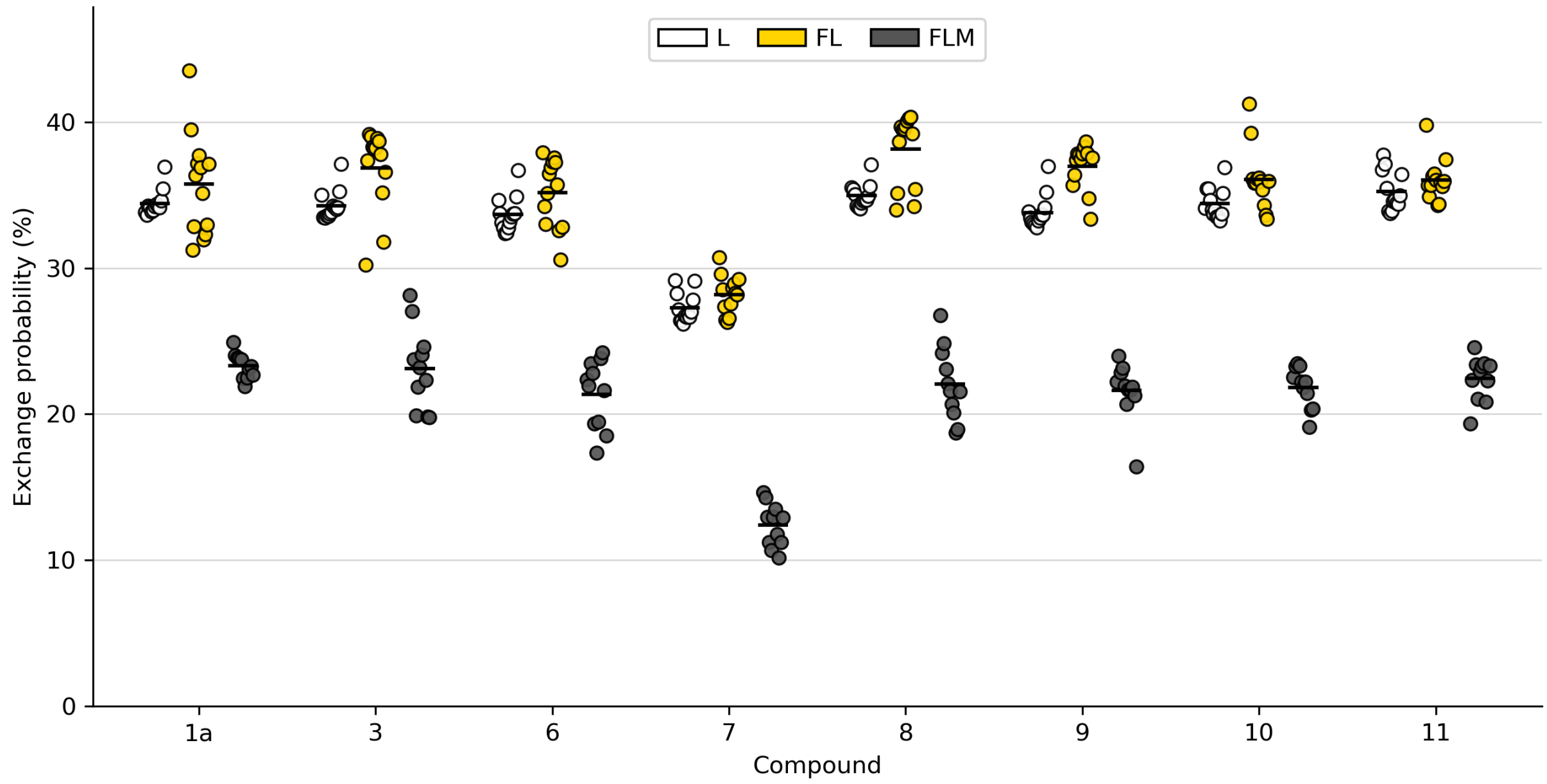

Figure S37: Exchange probability between the neighboring replicas in the REST2 simulations. Each data point represents an average acceptance ratio between replica i and its neighboring replica $i + 1$.

S5 Determination of Stabilized Geometries from the Binary and Ternary Complex Simulations by Frame-to-Frame Alignment

S5.1 Oversimplification of the θ_{lig} Representation

While the bending between the two planes can lead to negative θ_{lig} values, very negative θ_{lig} values were usually an artifact of the plane-fitting protocol when the MAPRE1-plane becomes far from planar. As an example, Figure S38 shows the negative angles observed for compound **10**. An angle of $-16.4°$ was observed when the MAPRE1-recognition core is mostly flat but the carbon atoms around the 4-piperidine moiety are bent downward (Figure S38A). The conformation with the most negative θ_{lig} value of $-40.99°$ observed for compound **10** in the FLM simulations is shown in Figure S38B. Importantly, although θ_{lig} is negative, this conformation does not correspond to any conformation with negative θ_{lig} sampled in the binary FL complex. Instead, the negative θ_{lig} value is an artifact of the poorly fitted plane because the MAPRE1-recognition core becomes non-planar. This shows that θ_{lig} oversimplifies the ligand conformation and is not suitable to describe the shape of the ligand in such conformations.

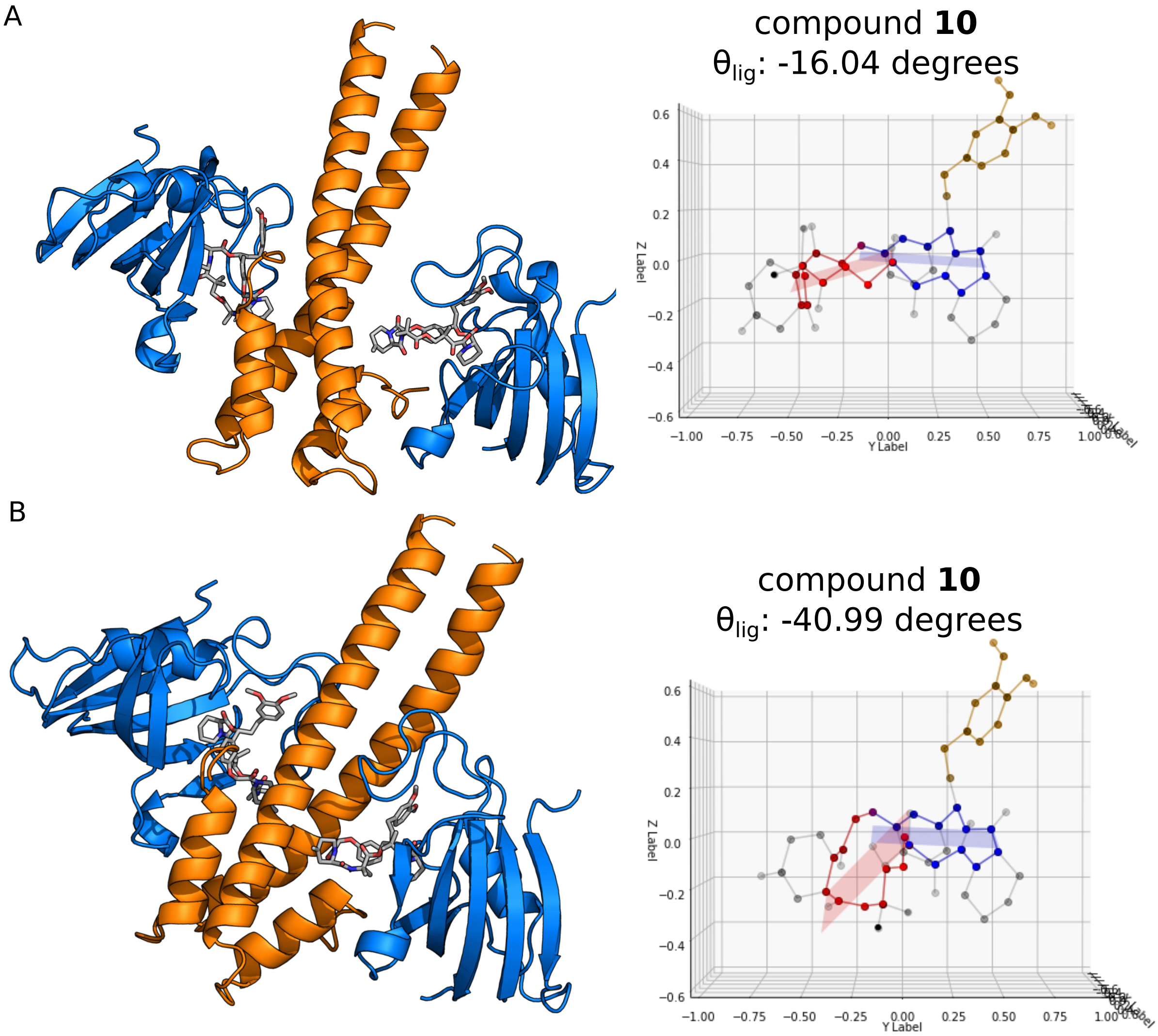

Figure S38: Exemplary ligand conformations from the REST2 simulation of the FLM complex with compound **10**. (A): Conformation with a negative θ_{lig} that shows a slightly bent configuration. (B): Conformation with a negative θ_{lig} that arises because of the non-planar configuration instead of an actual bending.

S5.2 Low Flexibility of FKBP12 in FL and FLM Complexes

In the REST2 simulations of both the FL and FLM complexes, FKBP12 shows a low flexibility despite the changes in the ligand conformation, with the C_α-RMSD mostly staying between 0.1 and 0.15 nm with respect to the first frame after the NPT equilibration (Figure S39). In contrast, MAPRE1 in the FLM ensembles is more flexible.

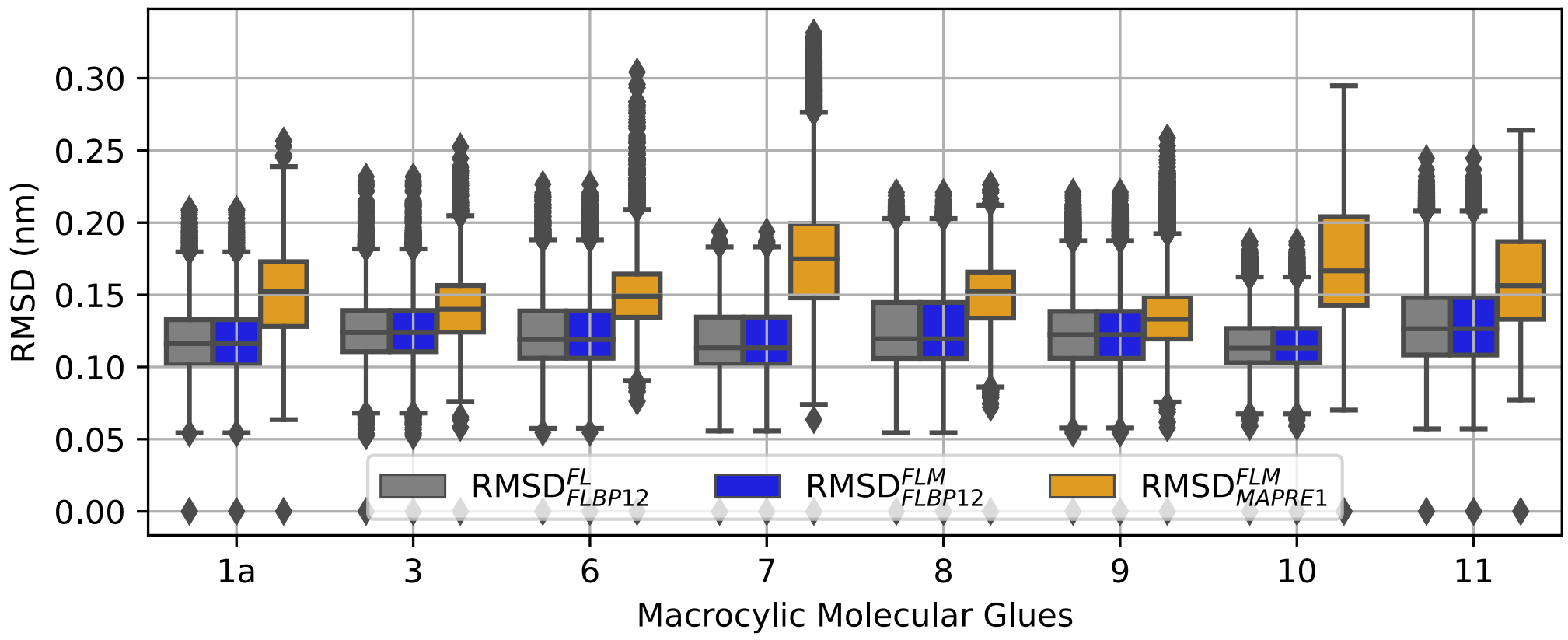

Figure S39: Protein flexibility in the REST2 simulations as measured by C_α-RMSD of the corresponding protein with respect to the first frame after the NPT equilibration. The lines in each box show the median of the distribution $\pm$ interquartile range $\times$ 1.5. FKBP12 is shown in gray (FL) and blue (FLM), MAPRE1 is shown in orange (FLM).

The high rigidity of FKBP12 in both FL and FLM complexes provides an alternative way to assess whether a configuration of the FL complex can be adopted by the FLM complexes. This was done by aligning FKBP12 frame-by-frame, i.e., for each frame in the FL simulation ($\mathbf{r}_{FL}$), FKBP12 was aligned to FKBP12 in each monomer (FLM is a dimer) in each frame of the FLM simulation ($\mathbf{r}_{FLM}$), and a possible steric collision was assessed between the ligand and MAPRE1. To define such a collision between the ligand from $\mathbf{r}_{FL}$ and MAPRE1 from $\mathbf{r}_{FLM}$ after the alignment, the minimal distance between any heavy atoms from MAPRE1 and the ligand was measured in the FLM ensembles. To determine the minimal distance criterion to flag collisions, we looked at the distribution of this distance in the FLM simulations (Figure S40). Across all active compounds, the distribution of the minimal distance is mostly above 0.26 nm, and thus, this value was used as the collision criterion.

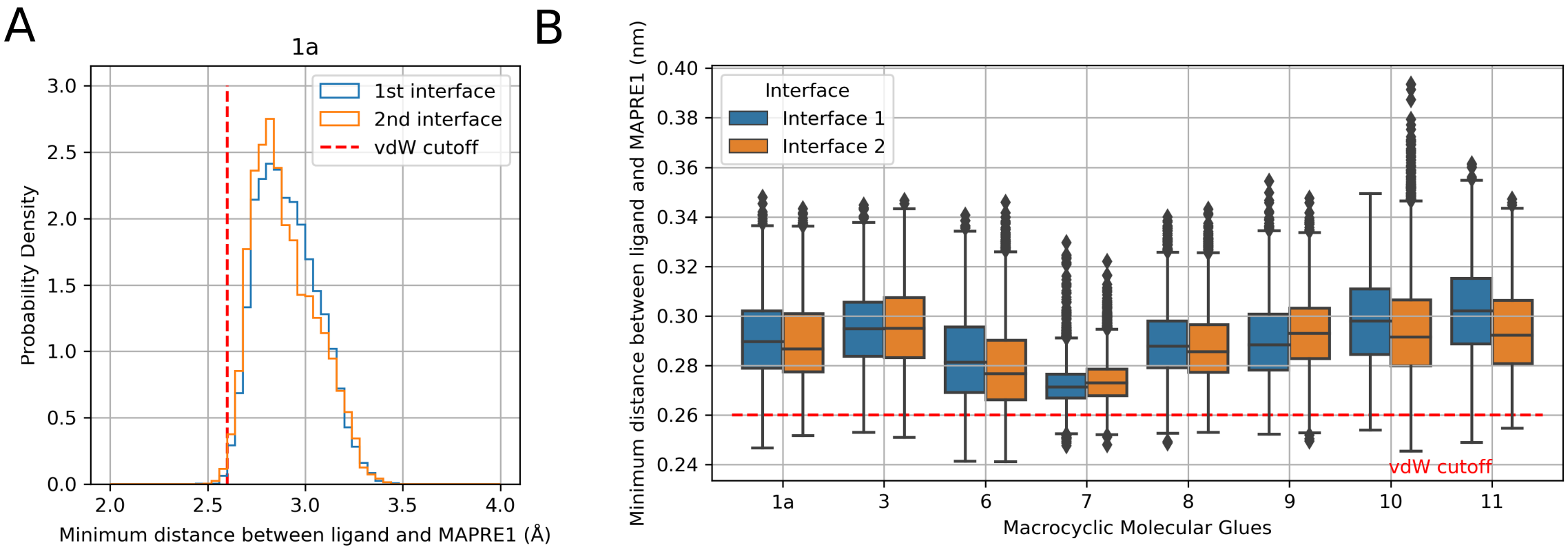

Figure S40: Minimal distance between MAPRE1 and the ligand in the FLM simulations. (A): Comparison of the distribution of this distance between the two interfaces of the FLM complex for compound **1a**. (B): Box plot of the minimal distance in the FLM simulations for all active compounds. The lines in each box show the median $\pm$ interquartile range $\times$ 1.5. The red dashed line is the cutoff of 0.26 nm.

Figure S41 illustrates how the frame-to-frame alignment method was implemented. First, FKBP12 from the FLM candidate configuration ($\mathbf{r}_{FLM}$) is aligned to FKBP12 from the FL candidate configuration ($\mathbf{r}_{FL}$). Due to the high rigidity of FKBP12, the aligned proteins show good overlap except for the slight mismatch in the loop region (Figure S41). Next, the minimal distance between the heavy atoms of the ligand in $\mathbf{r}_{FL}$ and MAPRE1 in $\mathbf{r}_{FLM}$ is measured. If the distance is > 0.26 nm, the configuration $\mathbf{r}_{FLM}$ is considered compatible with the configuration $\mathbf{r}_{FL}$. This is mathematically described by the indicator function $\chi(d_{min}^{L-M} - d_{cut}^{L-M})$, which returns 1 if $d_{min}^{L-M} > d_{cut}^{L-M}$ or 0 otherwise. A cutoff $d_{cut}^{L-M} = 0.26$ nm was used here.

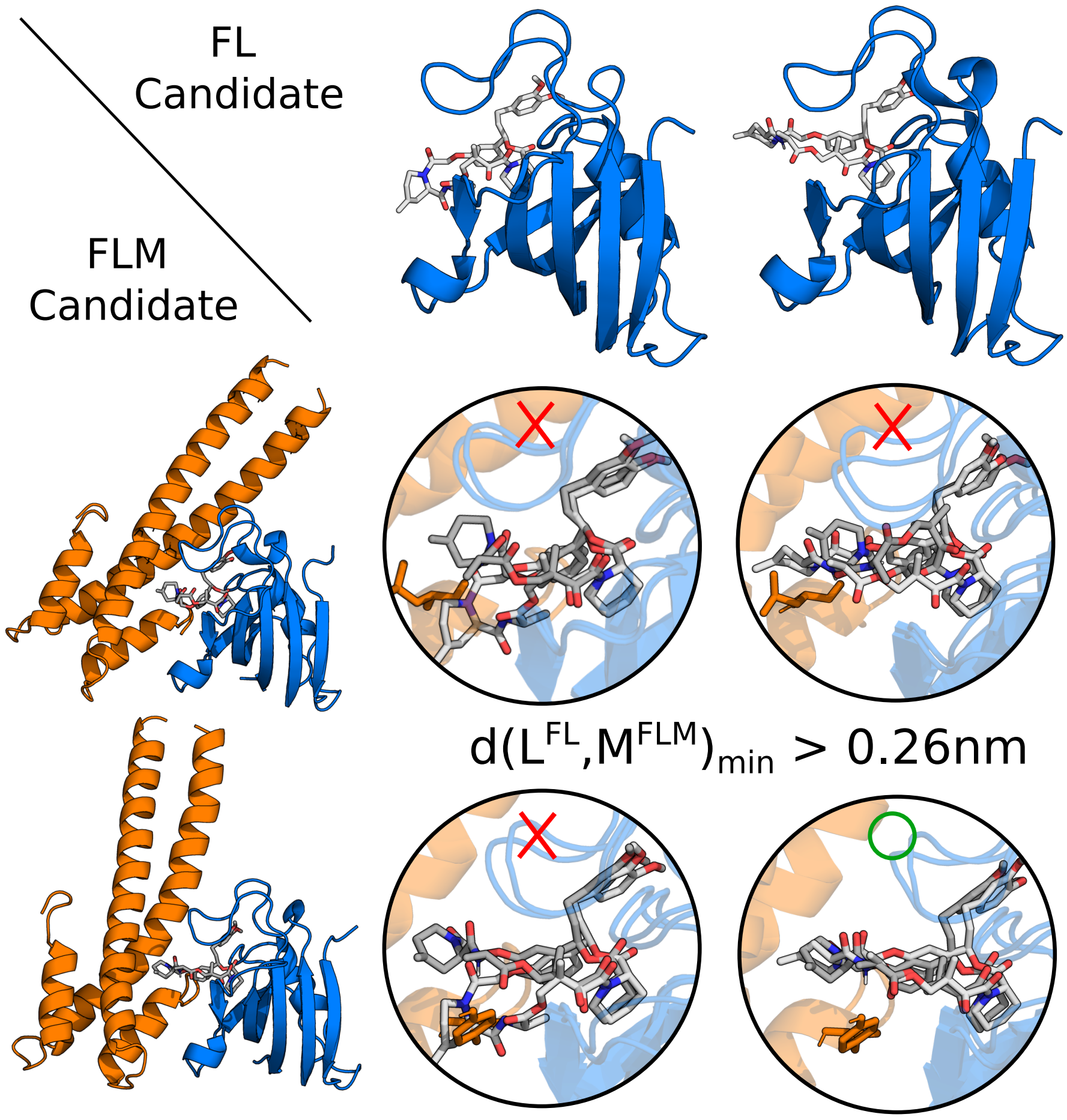

Figure S41: Illustration of the frame-to-frame alignment method. Given one FLM frame and one FL frame, the protein FKBP12 is aligned and the minimal distance between the ligand from the FL complex and MAPRE1 from the FLM complex is measured. The compatible FL and FLM geometry is indicated by a minimal distance > 0.26 nm (red crosses). Otherwise the geometries are incompatible (green circle).

By aligning each FLM frame ($\mathbf{r}_{FLM}$) to each FL frame ($\mathbf{r}_{FL}$), the geometric compatibility fraction was calculated as the ensemble average,

$$P^{acc}(\mathrm{r}_{FLM}) = \langle \chi(d_{min}^{L-M} - d_{cut}^{L-M}) \rangle_{FL}. \tag{4}$$

In other words, a configuration $\mathbf{r}_{FLM}$ has a probability $P^{acc}(\mathrm{r}_{FLM})$ to be sampled from the FL ensemble

without steric collision. To determine whether a FLM configuration is considered to be part of the stabilized geometry, a threshold of the $P^{acc}(\mathrm{r}_{FLM})$ needs to be set. Sensitivity analysis over thresholds near 1% showed similar ranking performance (Figure S42), and thus, 1% was used.

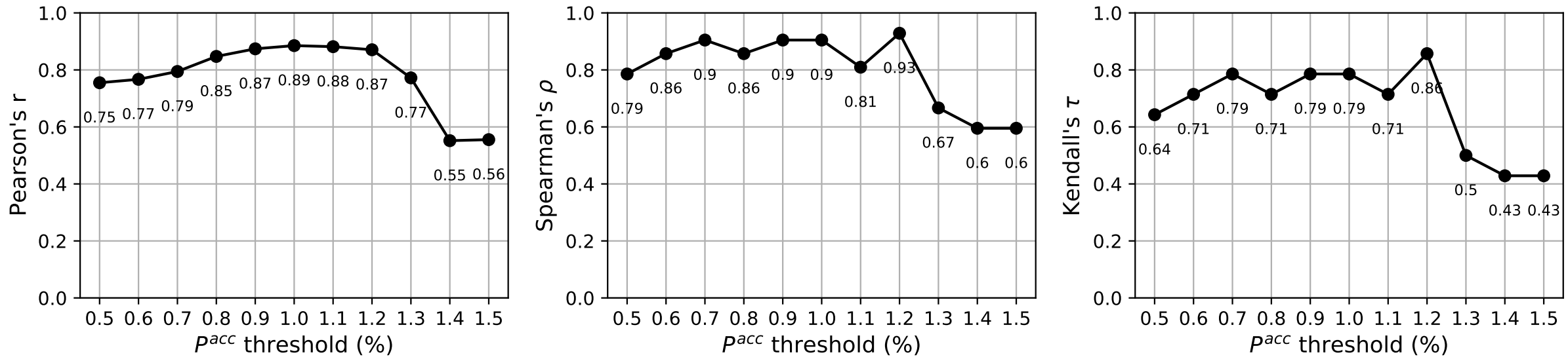

Figure S42: Effect of the choice of P^{acc} on the correlation metrics Pearson coefficient (left), Spearman coefficient (middle), and Kendall's τ (right) between the induced FKBP12-MAPRE1 interaction and the absolute cooperativity of the compounds.

S6 Computational Results and Titration Experiments for Binary and Ternary Assays for the Prospective Compounds

S6.1 θ_{lig} Distribution of the Prospective Compounds

Similar to the REST2 simulations from the known compounds shown in the main text, the prospective compounds also show slightly overlapping distributions in θ_{lig} (Figure S43).

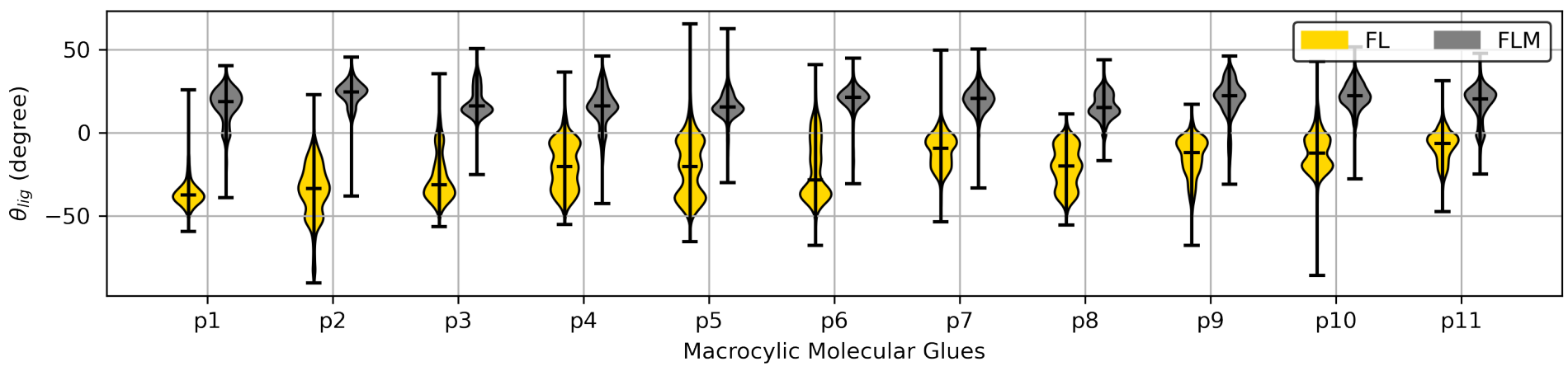

Figure S43: Distribution of θ_{lig} of the prospective compounds in the REST2 simulations of the binary FL (yellow) and ternary FLM (gray) systems.

S6.2 Titration Experiment with MAPRE1-Binding Assay

To compute the absolute cooperativity α_{abs} between ligand (L), MAPRE1 (M), and FKBP12 (F), four EC50 values must be obtained from binary and ternary assays in both binding pathways (FKBP12-first and MAPRE1-first pathways, see Figure 6A in the main text). However, in the MAPRE1-binding assays, signal saturation was not observed without the presence of FKBP12 even at the highest ligand concentration 400 μM (Figure S44). Therefore, only the apparent cooperativity α_{app}^{FKBP12} of the FKBP12-binding assay can be computed.

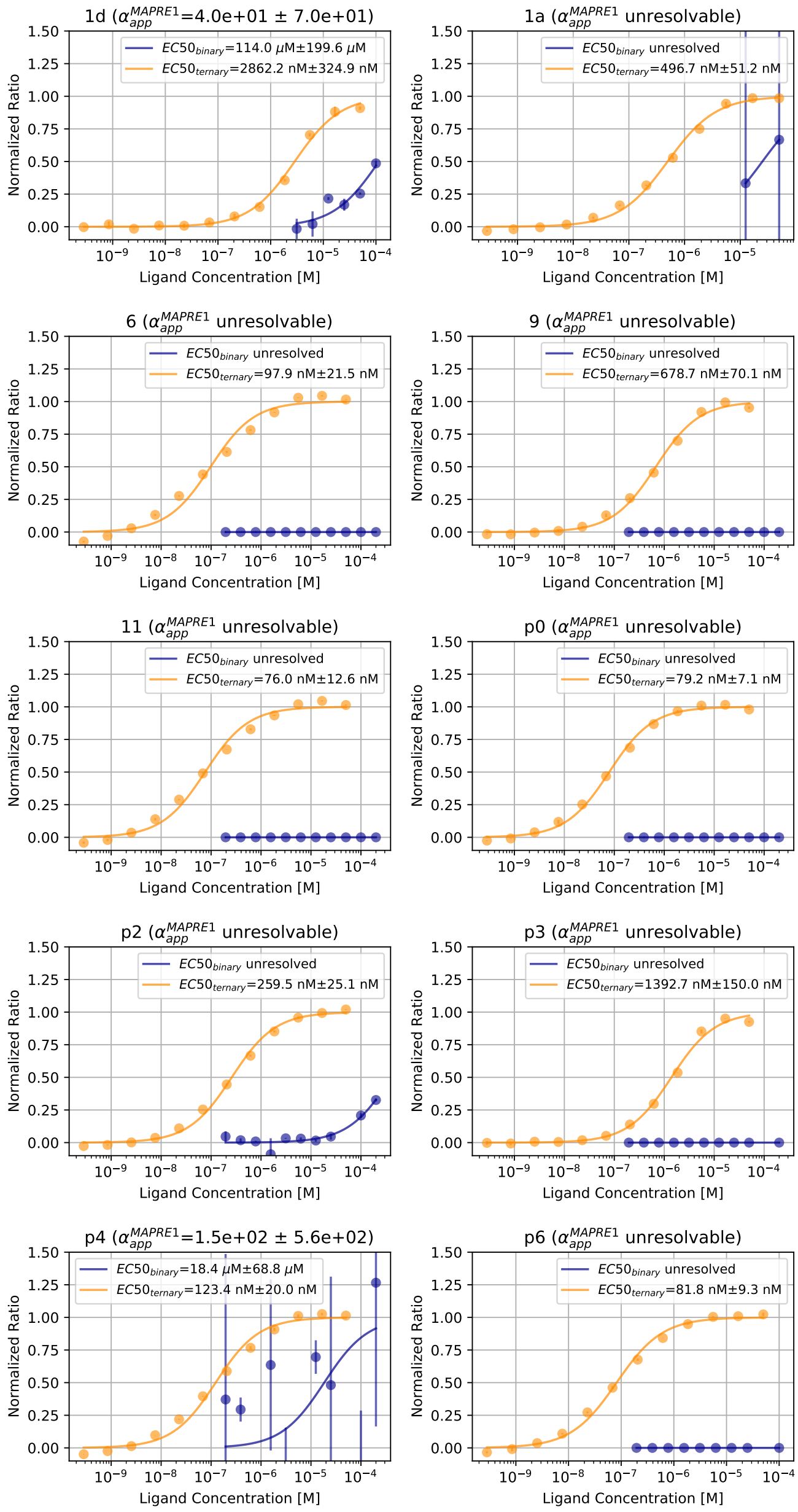

Figure S44: Binary and ternary titration experiment of the ligand with NHS-NT650-fluorophore-labeled MAPRE1 with normalized signal. The assays were performed at 5 nM MAPRE1 concentration with and without 10 μM FKBP12.

S6.3 EC50 Fitting of the FKBP12-Binding Titration Data

In the FKBP12-binding assays, sigmoid-like curves were observed in both binary and ternary assays. This allows for the estimation of the EC50 values by fitting the titration curve with the Hill binding equation (Figure S45). Although most of the titration experiments exhibit a nice sigmoid shape, compound **p6** shows a sudden drop in the signal between ligand concentrations at 0.1 μM and 1 μM in the binary

assays, and the unsaturated signal in the low-concentration regime in the ternary complex. Therefore, the estimated error for this compound is exceptionally large.

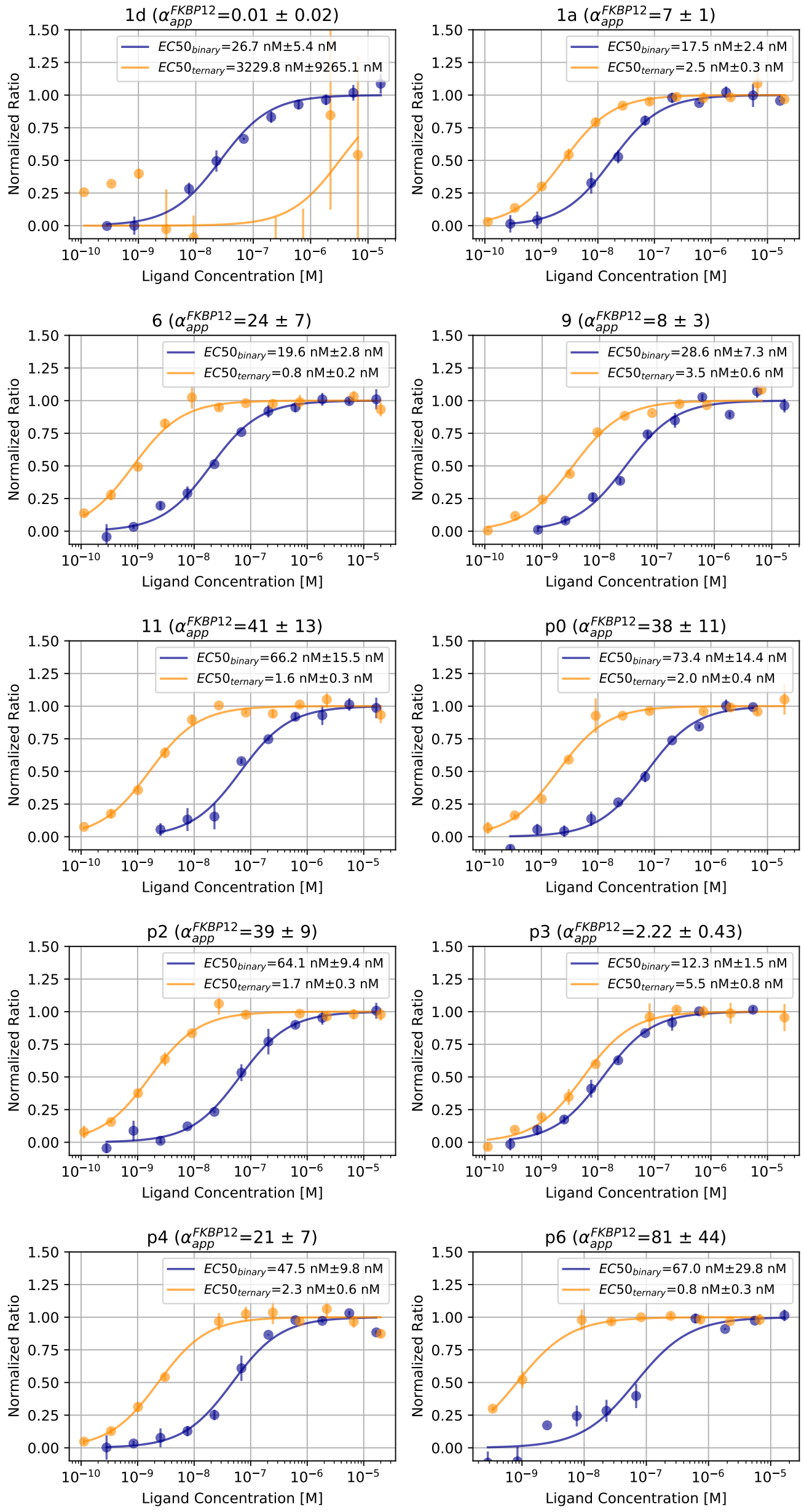

Figure S45: Binary and ternary titration experiment of the ligand with ED-tris-NTA 2nd gen labeled FKBP12. The assays were performed at 40 nM FKBP12 concentration with and without 10 μM MAPRE1.

S6.4 Non-normalized Fitting Curves and Filtered Data Points

Since binding assays detect binding events by monitoring the ratio of fluorescence intensity measured at wavelengths of 670 nm and 650 nm, the minimum and maximum signals as well as the direction

of curvature vary, as illustrated in Figure S46 for the FKBP12-binding assays and Figure S47 for the MAPRE1-binding assays. In addition, data points for which the duplicates show large variation were filtered out to avoid instability in the fitting. The filtered data points are shown in transparent color in Figures S46 and S47.

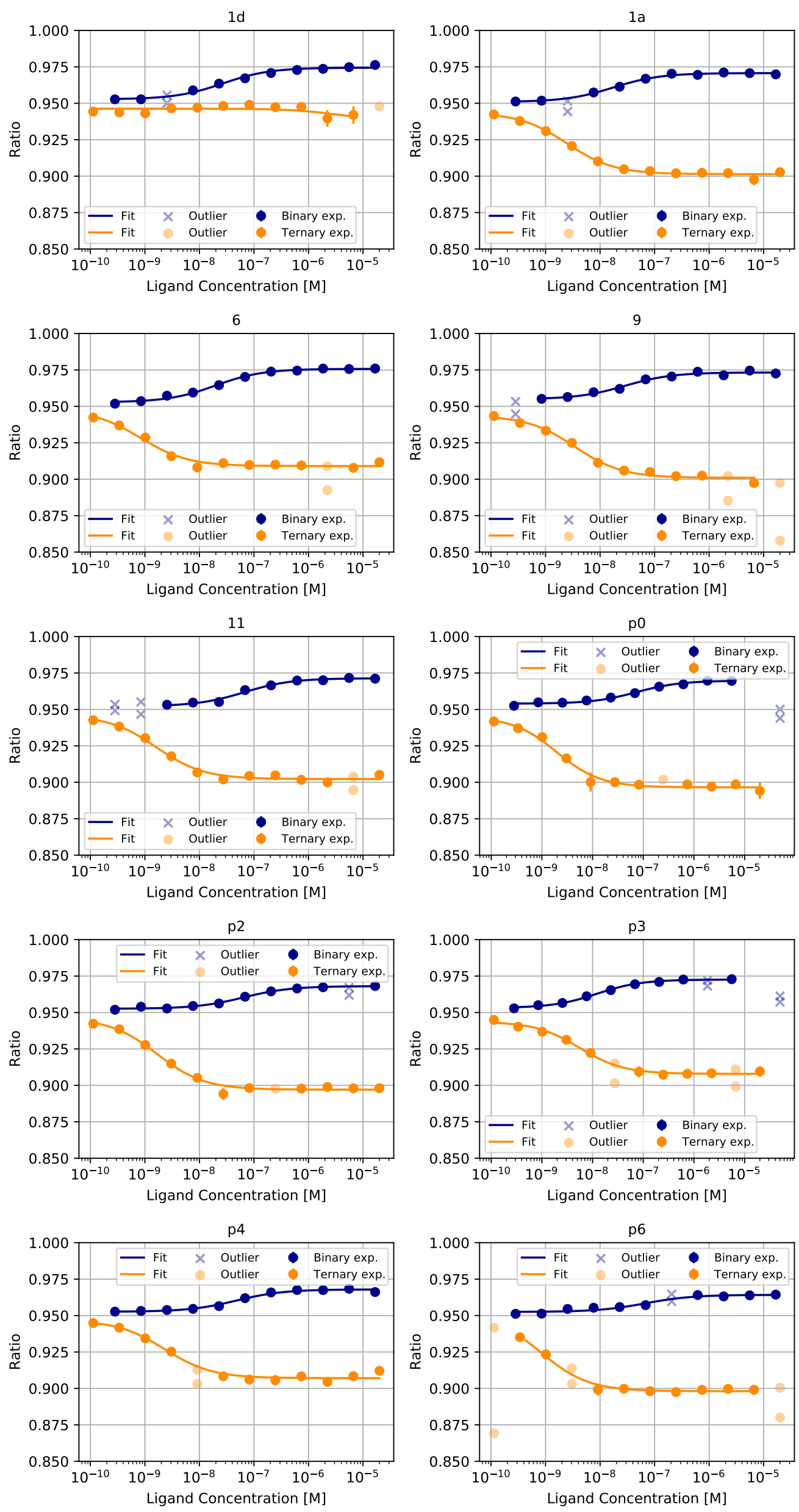

Figure S46: Non-normalized signal from the FKBP12-binding assays. Data points with a standard deviation larger than 10% of the min-max-difference between duplicates are shown in transparent color and are excluded from the fitting process.

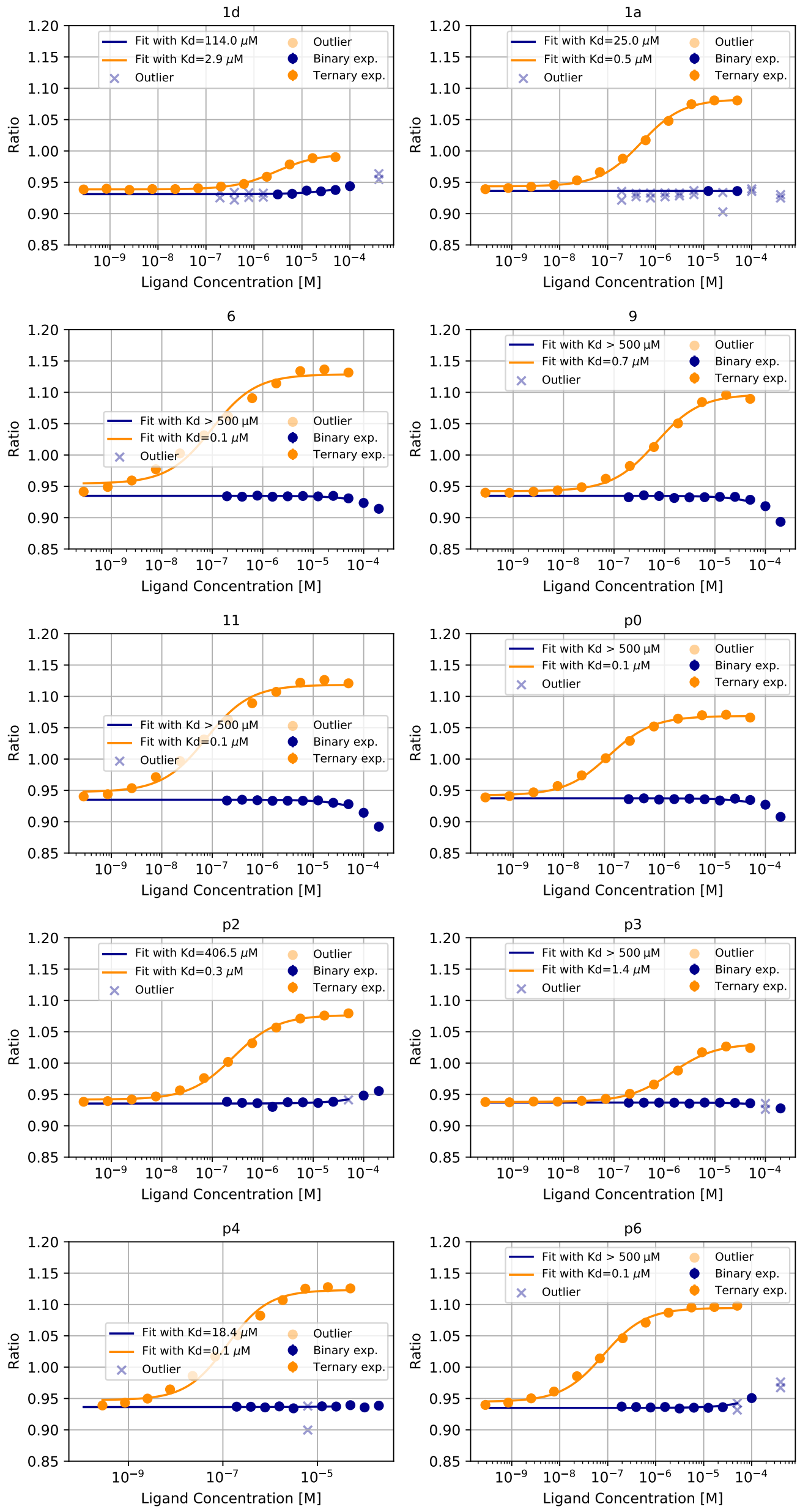

Figure S47: Non-normalized signal from the MAPRE1-binding assays. Data points with a standard deviation larger than 10% of the min-max-difference between duplicates are shown in transparent color and are excluded from the fitting process.

S7 Synthesis of Prospective Compounds

Precursor 1_1 was prepared according to Ref. [1].

S7.1 Synthesis of Compound p0

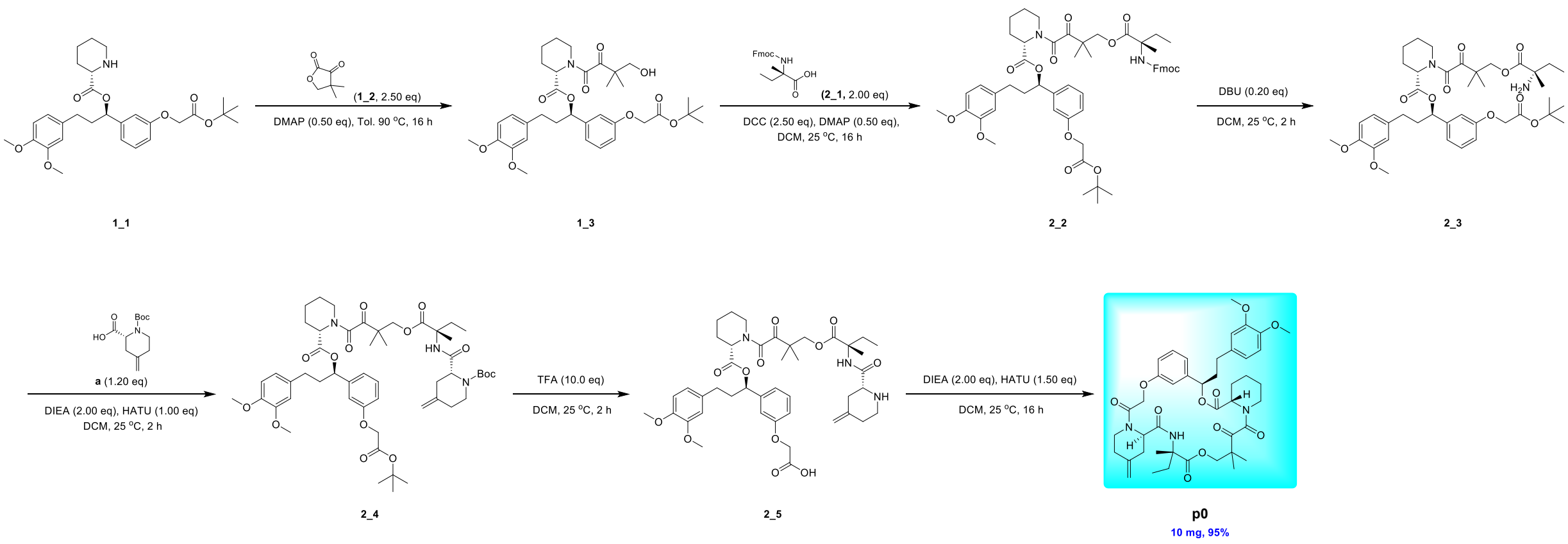

Figure S48: Synthesis scheme for compound **p0**.

Intermediate 1_3. A 250 mL one neck round bottom flask with a magnetic stir bar was charged with toluene (150 mL), followed by addition of 1_1 (12.0 g, 23.4 mmol, 1.00 eq), 1_2 (7.48 g, 58.4 mmol, 2.50 eq) and DMAP (1.43 g, 11.7 mmol, 0.50 eq) at 25 °C (external temperature). The mixture was stirred at 90 °C (external temperature) for 16 h. TLC (petroleum ether : ethyl acetate = 1:1, $R_f = 0.35$) indicated 1_1 was consumed completely, and several new spots were formed. The mixture was concentrated directly. The residue was purified by flash silica gel chromatography (ISCO®; X50 g SepaFlash® Silica Flash Column, eluent of 0–43% ethyl acetate/n-hexane gradient @ 80 mL/min) and the collected fraction was concentrated under reduced pressure to give the product. Compound 1_3 (12.0 g, 18.1 mmol, 77.4% yield, 96.7% purity) was obtained as colorless gum and confirmed by ^{1}H NMR, SFC, and LCMS.

Intermediate 2_2. To a solution of 1_3 (2.10 g, 3.27 mmol, 1.00 eq) in DCM (60 mL) was added 2_1 (2.22 g, 6.54 mmol, 2.00 eq). Then, DCC (1.69 g, 8.18 mmol, 1.65 mL, 2.50 eq) and DMAP (200 mg, 1.64 mmol, 0.50 eq) were added. The reaction mixture was stirred at 25 °C for 16 h. TLC (petroleum ether : ethyl acetate = 3:1, $R_f = 0.15$) indicated most of 1_3 was consumed, and several new spots were formed. The mixture was filtered, and the filtrate was concentrated directly. The residue was purified by flash silica gel chromatography (ISCO®; X25 g SepaFlash® Silica Flash Column, eluent of 0–50% ethyl acetate/n-hexane gradient @ 80 mL/min) and the collected fraction was concentrated under reduced pressure to give the product. Compound 2_2 (1.70 g, 1.72 mmol, 52.5% yield, 97.2% purity) was obtained as a white solid and confirmed by ^{1}H NMR, SFC, and LCMS.

Intermediate 2_3. A 100 mL one-neck round bottom flask with a magnetic stir bar was charged with DCM (16 mL), followed by addition of 2_2 (1.60 g, 1.66 mmol, 1.00 eq) and DBU (50.6 mg, 332 μmol, 50.1 μL, 0.20 eq) at 25 °C (external temperature). Then the mixture was stirred at 25 °C (external temperature) for 2 h. TLC (petroleum ether : ethyl acetate = 0:1, $R_f = 0.05$, UV) indicated 2_2 was consumed, and one main new spot was formed. The mixture was concentrated directly. The residue was

purified by flash silica gel chromatography (ISCO®; X25 g SepaFlash® Silica Flash Column, eluent of 0–100% n-hexane/ethyl acetate gradient @ 80 mL/min) and the collected fraction was concentrated under reduced pressure to give the product. Compound 2_3 (1.20 g, 1.59 mmol, 95.5% yield, 97.9% purity) was obtained as colorless gum and confirmed by ^{1}H NMR, SFC, and LCMS.

Intermediate 2_4. A 100 mL one-neck round bottom flask with a magnetic stir bar was charged with DCM (10 mL), followed by addition of 2_3 (1.10 g, 1.48 mmol, 1.00 eq), reagent a (430 mg, 1.78 mmol, 1.20 eq), DIEA (384 mg, 2.97 mmol, 517 μL, 2.00 eq) and HATU (565 mg, 1.48 mmol, 1.00 eq) at 25 °C (external temperature). The mixture was stirred at 25 °C (external temperature) for 2 h. LCMS showed 2_3 was consumed, and one peak was observed at RT = 1.728 min. M-1 = 962.6. TLC (n-hexane : ethyl acetate = 1:1, $R_f = 0.45$, UV) indicated 2_3 was consumed, and one main new spot was formed. The mixture was concentrated directly. The residue was purified by flash silica gel chromatography (ISCO®; X25 g SepaFlash® Silica Flash Column, eluent of 0–100% n-hexane/ethyl acetate gradient @ 80 mL/min) and the collected fraction was concentrated under reduced pressure to give the product. Compound 2_4 (1.40 g, 1.40 mmol, 93.96% yield, 96.07% purity) was obtained as colorless gum and confirmed by LCMS.

Intermediate 2_5. A 100 mL one-neck round bottom flask with a magnetic stir bar was charged with DCM (10 mL), followed by addition of 2_4 (1.40 g, 1.45 mmol, 1.00 eq) and TFA (1.66 g, 14.5 mmol, 1.08 mL, 10.0 eq) at 25 °C (external temperature). Then the mixture was stirred at 25 °C (external temperature) for 2 h. LCMS showed 2_4 was consumed, and one peak was observed at RT = 0.491 min. The mixture was concentrated directly. The mixture was used for next step directly without further purification. Compound 2_5 (1.30 g, 1.41 mmol, 97.11% yield, TFA salt) was obtained as a yellow solid and confirmed by SFC.

Compound p0. A 250 mL one-neck round bottom flask equipped with pressure equalizing addition funnel was charged with a magnetic stir bar, HATU (155 mg, 407 μmol, 1.50 eq), DIEA (70.1 mg, 542 μmol, 94.5 μL, 2.00 eq) dissolved in DCM (150 mL), 2_5 (250 mg, 271 μmol, 1.00 eq, TFA) in DCM (20 mL) was added dropwise to the above mixture slowly, then the mixture was stirred at 25 °C for 16 h. LCMS showed 2_5 was consumed, and one peak was observed at RT = 1.450 min. The mixture was concentrated directly. The mixture was purified by prep-HPLC (column: Waters Xbridge BEH C18 100*25mm*10um; mobile phase: [H_2O (10 mM NH_4HCO_3)-ACN]; gradient: 50%–85% B over 8.0 min). Compound **p0** (130 mg, 161 μmol, 59.33% yield, 97.7% purity) was obtained as a white solid and confirmed by ^{1}H NMR, SFC, and LCMS.

S7.2 Synthesis of Compound p2

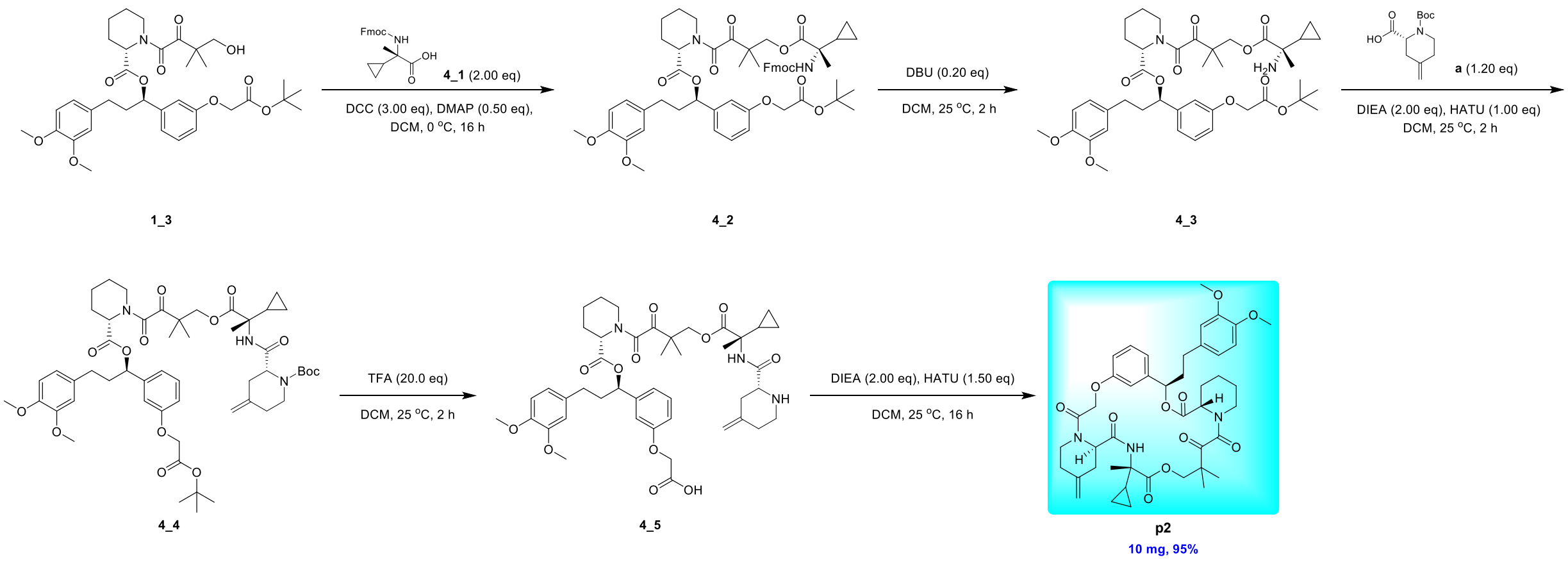

Figure S49: Synthesis scheme for compound **p2**.

Intermediate 4_2. To a solution of 1_3 (200 mg, 312 μmol, 1.00 eq) in DCM (5 mL) was added 4_1 (219.02 mg, 623.30 μmol, 2.00 eq). Next, DCC (161 mg, 779 μmol, 158 μL, 2.50 eq) and DMAP (19.0 mg, 156 μmol, 0.50 eq) were added. The reaction mixture was stirred at 0 °C for 16 h. TLC (petroleum ether : ethyl acetate = 2:1, $R_f = 0.30$) indicated 1_3 was remaining, and several new spots were formed. LCMS showed 1_3 was remaining, and one peak was observed at RT = 1.839 min. The mixture was filtered, and the filtrate was concentrated directly. The residue was purified by flash silica gel chromatography (ISCO®; X10 g SepaFlash® Silica Flash Column, eluent of 0–100% n-hexane/ethyl acetate gradient @ 80 mL/min) and the collected fraction was concentrated under reduced pressure to give the product. Compound 4_2 (880 mg, 665 μmol, 21.3% yield, 73.6% purity) was obtained as a white solid and detected by LCMS.

Intermediate 4_3. A 100 mL one-neck round bottom flask with a magnetic stir bar was charged with DCM (10 mL), followed by addition of 4_2 (880 mg, 665 μmol, 1.00 eq) and DBU (20.2 mg, 133 μmol, 20.0 μL, 0.20 eq) at 25 °C (external temperature). Then the mixture was stirred at 25 °C (external temperature) for 2 h. TLC (eetroleum ether : ethyl acetate = 0:1, $R_f = 0.05$, UV) indicated 4_2 was consumed, and one main new spot was formed. The mixture was concentrated directly. The residue was purified by flash silica gel chromatography (ISCO®; X4 g SepaFlash® Silica Flash Column, eluent of 0–100% n-hexane/ethyl acetate gradient @ 80 mL/min) and the collected fraction was concentrated under reduced pressure to give the product. Compound 4_3 (500 mg, 634 μmol, 95.5% yield, 95.5% purity) was obtained as colorless gum and confirmed by LCMS.

Intermediate 4_4. A 100 mL one-neck round bottom flask with a magnetic stir bar was charged with DCM (20 mL), followed by addition of 4_3 (500 mg, 664 μmol, 1.00 eq), reagent a (192 mg, 797 μmol, 1.20 eq), DIEA (172 mg, 1.33 mmol, 231 μL, 2.00 eq) and HATU (253 mg, 664 μmol, 1.00 eq) at 25 °C (external temperature). The mixture was stirred at 25 °C (external temperature) for 2 h. LCMS showed 4_3 was consumed, and one peak was observed at RT = 1.723 min. TLC (petroleum ether : ethyl acetate = 1:1, $R_f = 0.45$, UV) indicated 4_3 was consumed, and one main new spot was formed. The mixture was concentrated directly. The residue was purified by flash silica gel chromatography (ISCO®; X2 g

SepaFlash® Silica Flash Column, eluent of 0–100% n-hexane/ethyl acetate gradient @ 60 mL/min) and the collected fraction was concentrated under reduced pressure to give the product. Compound 4_4 (600 mg, 530 μmol, 79.8% yield, 86.2% purity) was obtained as colorless gum and confirmed by LCMS.

Intermediate 4_5. A 40 mL vial with a magnetic stir bar was charged with DCM (5 mL), followed by addition of 4_4 (300 mg, 307 μmol, 1.00 eq) and TFA (700 mg, 6.15 mmol, 456 μL, 20.0 eq) at 25 °C (external temperature). Then the mixture was stirred at 25 °C (external temperature) for 2 h. LCMS showed 4_4 was consumed, and one peak was observed at RT = 0.482 min. The mixture was concentrated directly. The mixture was used for next step directly without further purification. Compound 4_5 (280 mg, 249 μmol, 81.1% yield, 83.1% purity, TFA salt) was obtained as a yellow solid and confirmed by LCMS.

Compound p2. A 250 mL one-neck round bottom flask equipped with pressure equalizing addition funnel was charged with a magnetic stir bar, DIEA (116 mg, 899 μmol, 157 μL, 3.00 eq), HATU (171 mg, 450 μmol, 1.50 eq) dissolved in DCM (200 mL), 4_5 (280 mg, 300 μmol, 1.00 eq, TFA salt) in DCM (60 mL) was added dropwise to the above mixture slowly, then the mixture was stirred at 25 °C for 16 h. LCMS showed 4_5 was consumed, and one peak was observed at RT = 1.488 min. The mixture was concentrated directly. The mixture was purified by prep-HPLC (column: Waters Xbridge BEH C18 100*25mm*10um; mobile phase: [H_2O (10 mM NH_4HCO_3)-ACN]; gradient: 55%–85% B over 8.0 min). Compound **p2** (10.42 mg, 12.72 μmol, 4.24% yield, 97.885% purity) was obtained as a white solid and confirmed by ^{1}H NMR, SFC, and LCMS.

S7.3 Synthesis of Compound p3

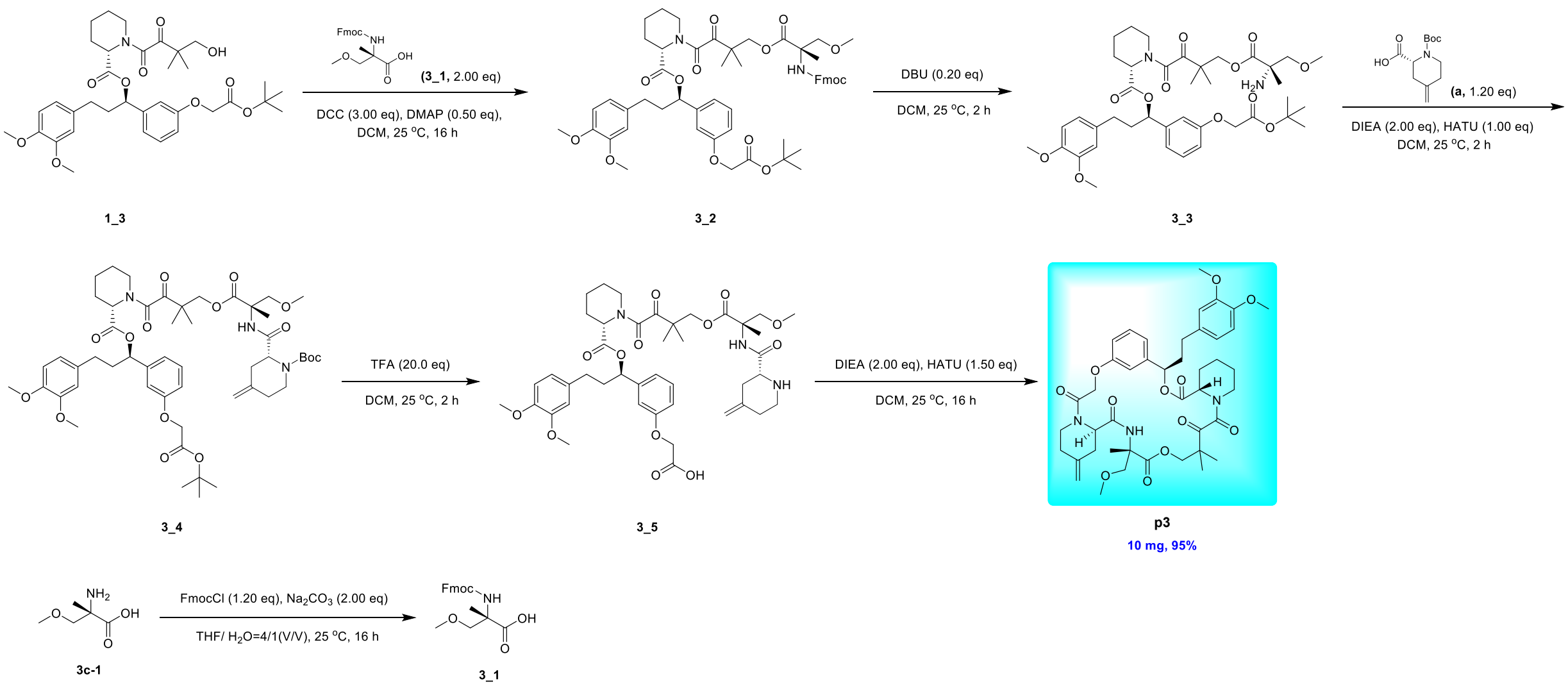

Figure S50: Synthesis scheme for compound **p3**.

Intermediate 3_1. A 100 mL one neck-round bottom flask with a magnetic stir bar was charged with THF (20 mL)/H_2O (5 mL), followed by addition of 3c_1 (1.00 g, 7.51 mmol, 1.00 eq), Fmoc-Cl (2.33 g, 9.01 mmol, 1.20 eq) and Na_2CO_3 (1.59 g, 15.0 mmol, 2.00 eq) at 25 °C (external temperature). Then the mixture was stirred at 25 °C (external temperature) for 16 h. LCMS showed 3c_1 was consumed, and

one peak was observed at RT = 0.896 min. TLC (n-hexane : EtOAc = 0:1, PMA) indicated reactant $3c$_1 ($R_f = 0.00$) was fully consumed and one major new spot ($R_f = 0.10$) was detected. The mixture was poured into 30 mL H_2O at 25 °C (external temperature) and then extracted with EtOAc (20 mL × 3). The combined organic layers were washed with H_2O (20 mL × 3), dried over Na_2SO_4. Then the Na_2SO_4 was filtered off and further washed with 10 mL EtOAc. The obtained filtrate was concentrated under reduced pressure to give a residue. The residue was purified by flash silica gel chromatography (ISCO®; X5 g SepaFlash® Silica Flash Column, eluent of 0–100% ethyl acetate/n-hexane gradient @ 60 mL/min) and the collected fraction was concentrated under reduced pressure to give the product. Compound 3_1 (1.00 g, 2.81 mmol, 37.47% yield) was obtained as a white solid and confirmed by ^{1}H NMR.

Intermediate 3_2. To a solution of 1_3 (1.00 g, 1.56 mmol, 1.00 eq) in DCM (30 mL) was added 3_1 (1.00 g, 2.81 mmol, 1.81 eq). After that, DCC (965 mg, 4.67 mmol, 946 μL, 3.00 eq) and DMAP (95.2 mg, 779 μmol, 0.50 eq) were added. The reaction mixture was stirred at 25 °C for 16 h. TLC (petroleum ether : ethyl acetate = 2:1, $R_f = 0.13$) indicated 1_3 was remaining, and several new spots were formed. LCMS showed 1_3 was remaining, and one peak with desired mass was observed at RT = 1.825 min. The mixture was filtered, and the filtrate was concentrated directly. The residue was purified by flash silica gel chromatography (ISCO®; X10 g SepaFlash® Silica Flash Column, eluent of 0–100% n-hexane/ethyl acetate gradient @ 80 mL/min) and the collected fraction was concentrated under reduced pressure to give the product. Compound 3_2 (0.40 g, 409 μmol, 26.22% yield) was obtained as a white solid.

Intermediate 3_3. A 100 mL one-neck round bottom flask with a magnetic stir bar was charged with DCM (4 mL), followed by addition of 3_2 (0.40 g, 409 μmol, 1.00 eq) and DBU (12.4 mg, 81.7 μmol, 12.3 μL, 0.20 eq) at 25 °C (external temperature). Then the mixture was stirred at 25 °C (external temperature) for 2 h. TLC (petroleum ether : ethyl acetate = 0:1, $R_f = 0.05$, UV) indicated 3_2 was consumed, and one main new spot was formed. The mixture was concentrated directly. The residue was purified by flash silica gel chromatography (ISCO®; X25 g SepaFlash® Silica Flash Column, eluent of 0–100% n-hexane/ethyl acetate gradient @ 80 mL/min) and the collected fraction was concentrated under reduced pressure to give the product. Compound 3_3 (220 mg, 236.3 μmol, 57.8% yield, 81.3% purity) was obtained as colorless gum and confirmed by LCMS.

Intermediate 3_4. A 40 mL vial with a magnetic stir bar was charged with DCM (5 mL), followed by addition of 3_3 (220 mg, 291 μmol, 1.00 eq), reagent a (84.2 mg, 349 μmol, 1.20 eq), DIEA (75.1 mg, 581 μmol, 101 μL, 2.00 eq) and HATU (111 mg, 291 μmol, 1.00 eq) at 25 °C (external temperature). The mixture was stirred at 25 °C (external temperature) for 2 h. LCMS showed 3_3 was consumed, and one peak was observed at RT = 1.712 min. TLC (petroleum ether : ethyl acetate = 1:1, $R_f = 0.45$, UV) indicated 3_3 was consumed and one main new spot was formed. The mixture was concentrated directly. The residue was purified by flash silica gel chromatography (ISCO®; X2 g SepaFlash® Silica Flash Column, eluent of 0–100% n-hexane/ethyl acetate gradient @ 60 mL/min) and the collected fraction was concentrated under reduced pressure to give the product. Compound 3_4 (200 mg, 152 μmol, 52.2% yield, 74.4% purity) was obtained as colorless oil and confirmed by LCMS.

Intermediate 3_5. A 40 mL vial with a magnetic stir bar was charged with DCM (2 mL), followed by addition of 3_4 (200 mg, 204 μmol, 1.00 eq) and TFA (465 mg, 4.08 mmol, 303 μL, 20.0 eq) at 25 °C (external temperature). Then the mixture was stirred at 25 °C (external temperature) for 2 h. LCMS showed 3_4 was consumed, and one peak was observed at RT = 1.566 min. The mixture was concentrated

directly. The mixture was used for next step directly without further purification. Compound 3_5 (160 mg, 106.93 μmol, 52.40% yield, 62.683% purity, TFA salt) was obtained as a white solid and confirmed by LCMS.

Compound p3. A 250 mL one-neck round bottom flask equipped with pressure equalizing addition funnel was charged with a magnetic stir bar, DIEA (27.6 mg, 214 μmol, 37.3 μL, 2.00 eq), HATU (61.0 mg, 160 μmol, 1.50 eq) dissolved in DCM (100 mL), 3_5 (160 mg, 107 μmol, 1.00 eq, TFA) in DCM (30 mL) was added dropwise to the above mixture slowly, then the mixture was stirred at 25 °C for 16 h. LCMS showed 3_5 was consumed, and one peak was observed at RT = 1.428 min. The mixture was concentrated directly. The mixture was purified by prep-HPLC (column: Waters Xbridge BEH C18 100*25mm*10um; mobile phase: [H_2O (10 mM NH_4HCO_3)-ACN]; gradient: 50%–80% B over 8.0 min). Compound **p3** (11.07 mg, 13.7 μmol, 12.8% yield, 100% purity) was obtained as a white solid and confirmed by ^{1}H NMR, SFC, and LCMS.

S7.4 Synthesis of Compound p4

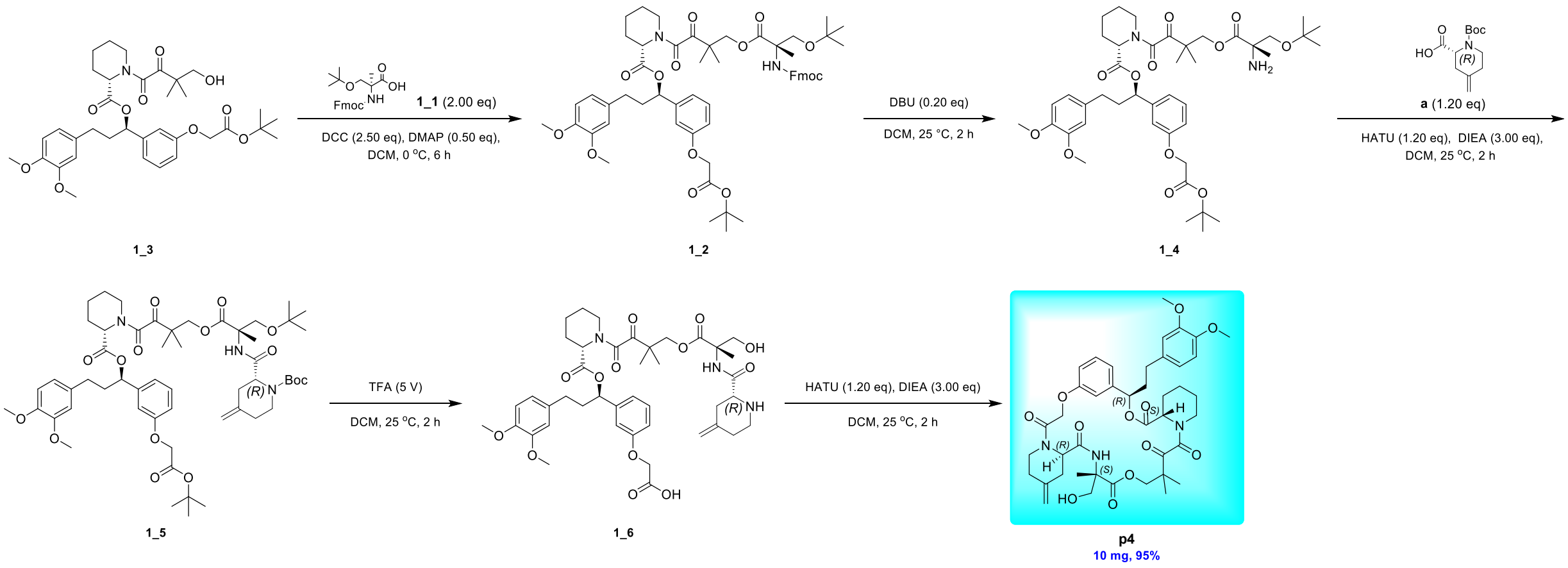

Figure S51: Synthesis scheme for compound **p4**.

Compound 1_2. A 40 mL vial with a magnetic stir bar was charged with DCM (10 mL), followed by addition of 1_3 (800 mg, 1.25 mmol, 1.00 eq), 1_1 (991 mg, 2.49 mmol, 2.00 eq), DCC (643 mg, 3.12 mmol, 630 μL, 2.50 eq) and DMAP (76.2 mg, 623 μmol, 0.50 eq) at 0 °C. The mixture was purged with N_2 for 2 min before vigorously stirring at 0 °C for 6 h. LCMS indicated 14.8% of 1_3 (RT = 0.613 min) remained in the reaction mixture, and 43.8% of peak with desired mass value was detected at RT = 0.784 min. TLC (commercial hexanes : ethyl acetate = 2:1, UV) indicated 1_3 (R_f = 0.24) was remaining, and one major new spot (R_f = 0.44) was detected. The reaction mixture was filtered, and the filtrate was concentrated under reduced pressure to give a residue. The residue was purified by flash silica gel chromatography (ISCO®; 40 g SepaFlash® Silica Flash Column, eluent of 0–30% ethyl acetate/n-hexane gradient @ 60 mL/min) and the collected fraction was concentrated under reduced pressure to give the product. Compound 1_2 (1.00 g, 979 μmol, 78.6% yield) was obtained as colorless oil.

Compound 1_4. A 40 mL vial with a magnetic stir bar was charged with DCM (10 mL), followed by addition of 1_2 (1.00 g, 979 μmol, 1.00 eq) and DBU (29.8 mg, 196 μmol, 29.5 μL, 0.20 eq) at 25 °C.

The mixture was stirring at 25 °C for 2 h. LCMS indicated 1_2 was fully consumed, and 37.2% of peak with desired mass value was observed at RT = 0.578 min. TLC (commercial hexanes : ethyl acetate = 0:1, phosphomolybdic acid) indicated 1_2 (R_f = 0.71) was consumed completely, and one major new spot (R_f = 0.16) was detected. The reaction mixture was concentrated under reduced pressure to give a residue. The residue was diluted with 30 mL H_2O and extracted with 30 mL DCM (10 mL x 3). The combined organic layers were dried over Na_2SO_4, filtered and the filtrate was concentrated under reduced pressure to give a residue. The residue was purified by flash silica gel chromatography (ISCO®; 12 g SepaFlash® Silica Flash Column, eluent of 0–80% ethyl acetate/n-hexane gradient @ 60 mL/min) and the collected fraction was concentrated under reduced pressure to give the product. Compound 1_4 (655 mg, 819 μmol, 83.7% yield) was obtained as colorless oil.

Compound 1_5. A 40 mL vial with a magnetic stir bar was charged with DCM (7 mL), followed by addition of 1_4 (655 mg, 819 μmol, 1.00 eq), reagent a (237 mg, 984 μmol, 1.20 eq), DIEA (318 mg, 2.46 mmol, 428 μL, 3.00 eq) and HATU (374 mg, 984 μmol, 1.20 eq) at 25 °C. The mixture was stirring at 25 °C for 2 h. LCMS indicated 1_4 was fully consumed, and 64.0% of peak with desired mass value was observed at RT = 1.817 min. TLC (commercial hexanes : ethyl acetate = 2:1, phosphomolybdic acid) indicated reactant 1_4 (R_f = 0.10) was consumed completely, and one major new spot (R_f = 0.63) was detected. The reaction mixture was diluted with 30 mL H_2O and extracted with 30 mL EtOAc (10 mL × 3). The combined organic layers were dried over Na_2SO_4, filtered and the filtrate was concentrated under reduced pressure to give a residue. The residue was purified by flash silica gel chromatography (ISCO®; 12 g SepaFlash® Silica Flash Column, eluent of 0–50% ethyl acetate/n-hexane gradient @ 60 mL/min) and the collected fraction was concentrated under reduced pressure to give the product. Compound 1_5 (1.00 g, crude) was obtained as colorless oil.

Compound 1_6. A 40 mL vial with a magnetic stir bar was charged with DCM (10 mL), followed by addition of 1_5 (1.00 g, 978 μmol, 1.00 eq) and TFA (7.68 g, 67.3 mmol, 5 mL, 68.8 eq) at 25 °C. The mixture was stirring at 25 °C for 2 h. LCMS indicated 1_5 was fully consumed, and 55.1% of peak with desired mass value was observed at RT = 0.454 min. The reaction mixture was concentrated under reduced pressure to give a residue. Then the residue was diluted with ACN (10 mL) and adjusted to pH = 7 with DIEA. The mixture was concentrated under reduced pressure to give a crude product. This crude product used for the next step without purification. Compound 1_6 (1.50 g, crude) was obtained as yellow oil.

Compound p4. A 100 mL one-neck round bottom flask with a magnetic stir bar was charged with DCM (15 mL), followed by addition of DIEA (478 mg, 3.70 mmol, 645 μL, 3.00 eq) and HATU (563 mg, 1.48 mmol, 1.20 eq) at 25 °C. Then 1_6 (1.00 g, 1.23 mmol, 1.00 eq) in DCM (15 mL) was added to the mixture at 25 °C. The mixture was stirring at 25 °C for 2 h. LCMS indicated 1_6 was fully consumed, and 30.5% of peak with desired mass value was observed at RT = 1.336 min. The crude reaction mixture was concentrated under reduced pressure directly to give the crude product. The residue was purified by prep-HPLC (column: WePure Biotech XPt C18 150×50×7 μm; mobile phase: [H_2O (10 mM NH_4HCO_3)–ACN]; gradient: 40–70% B over 10.0 min). The collected fraction underwent lyophilization to give the desired product. Compound **p4** (12.1 mg, 15.1 μmol, 1.22% yield, 99.0% purity) was obtained as a white solid and confirmed by HPLC, ^{1}H NMR, LCMS and SFC.

S7.5 Synthesis of Compound p6

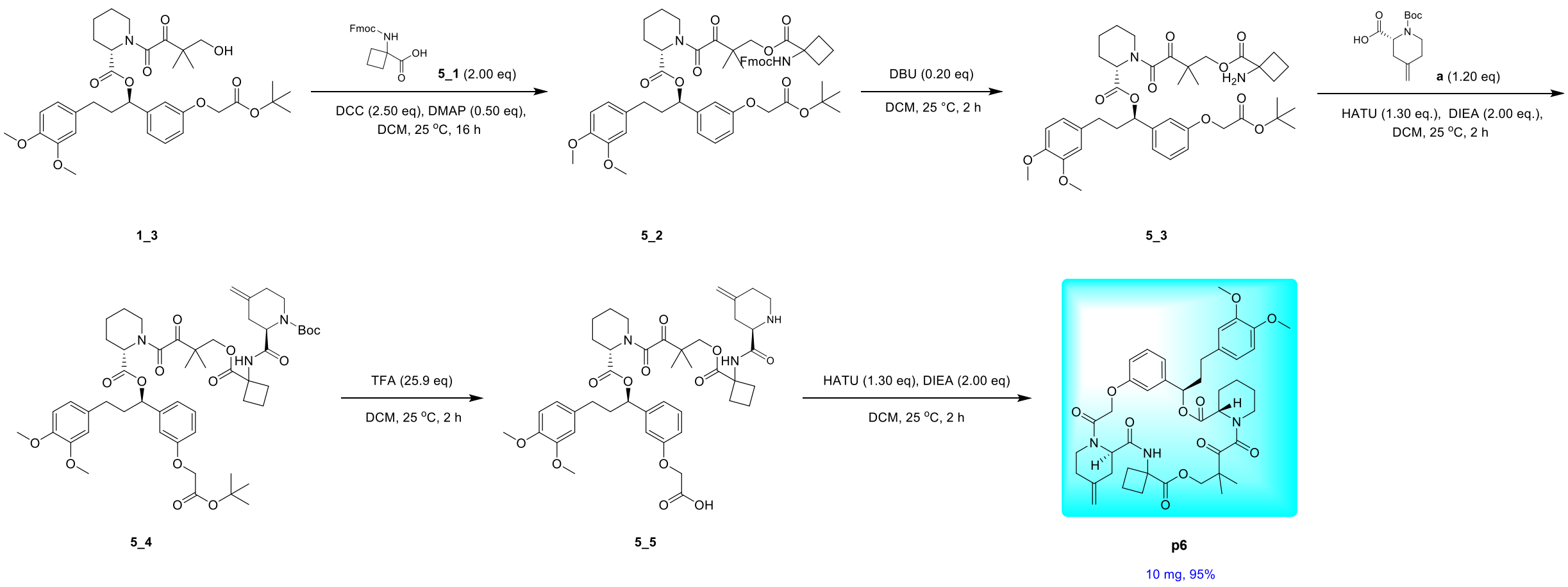

Figure S52: Synthesis scheme for compound **p6**.

Intermediate 5_2. A 250 mL 3-neck round bottom flask (with a refluxing condenser) with a magnetic stir bar was charged with DCM (60 mL), followed by the addition of 1_3 (2.00 g, 3.12 mmol, 1.00 eq), 5_1 (2.10 g, 6.23 mmol, 2.00 eq), DCC (1.61 g, 7.79 mmol, 1.58 mL, 2.50 eq) and DMAP (190 mg, 1.56 mmol, 0.50 eq) at 25 °C (internal temperature). Then the reaction mixture was allowed to stir vigorously at 25 °C (internal temperature) for 16 h. TLC (n-hexane : EtOAc = 2:1, UV) indicated 1_3 ($R_f = 0.32$) was consumed completely, and one major new spot ($R_f = 0.45$) was detected. The reaction mixture was filtered through a pad of paper under reduced pressure and the filter cake was rinsed with 10 mL DCM. The filtrate was concentrated under reduced pressure to give a residue. The residue was purified by flash silica gel chromatography (ISCO®; 80 g SepaFlash® Silica Flash Column, eluent of 0–40% ethyl acetate/n-hexane gradient @ 100 mL/min) and the collected fraction was concentrated under reduced pressure to give the product. Compound 5_2 (2.90 g, 2.29 mmol, 73.6% yield, 76.1% purity) was obtained as colorless oil and confirmed by LCMS.

Intermediate 5_3. A 100 mL one-neck round bottom flask with a magnetic stir bar was charged with DCM (26 mL), followed by addition of 5_2 (2.60 g, 2.71 mmol, 1.00 eq) and DBU (82.4 mg, 541 μmol, 81.6 μL, 0.20 eq) at 25 °C (external temperature). The mixture was stirred at 25 °C (external temperature) for 2 h. LCMS indicated 5_2 was fully consumed, and a peak with desired mass value was observed at RT = 0.515 min. TLC (n-hexane : EtOAc = 0:1, phosphomolybdic acid) indicated reactant 5_2 ($R_f = 0.73$) was consumed completely and one major new spot ($R_f = 0.31$) was detected. The crude reaction mixture was concentrated under reduced pressure directly to give the crude product. The residue was purified by flash silica gel chromatography (ISCO®; 40 g SepaFlash® Silica Flash Column, eluent of 0–100% ethyl acetate/n-hexane gradient @ 60 mL/min) and the collected fraction was concentrated under reduced pressure to give the product. Compound 5_3 (1.60 g, 2.17 mmol, 80.1% yield) was obtained as colorless oil and confirmed by ^{1}H NMR and SFC.

Intermediate 5_4. A 100 mL one-neck round bottom flask with a magnetic stir bar was charged with DCM (14 mL), followed by addition of 5_3 (1.50 g, 2.03 mmol, 1.00 eq), reagent **a** (588 mg, 2.44 mmol, 1.20 eq), DIEA (525 mg, 4.06 mmol, 707 μL, 2.00 eq) and HATU (1.00 g, 2.64 mmol, 1.30 eq) at 25 °C

(external temperature). The mixture was stirred at 25 °C (external temperature) for 2 h. LCMS indicated 5_3 was fully consumed, and a peak with desired mass value was observed at RT = 0.711 min. TLC (n-hexane : EtOAc = 2:1, UV) indicated 5_3 ($R_f = 0.26$) was consumed completely, and one major new spot ($R_f = 0.64$) was detected. The residue was diluted with 100 mL H_2O and extracted with DCM (30 mL × 3). The combined organic layers were dried over Na_2SO_4, filtered and the filtrate was concentrated under reduced pressure to give a residue. The residue was purified by flash silica gel chromatography (ISCO®; 120 g SepaFlash® Silica Flash Column, Eluent of 0–50% Ethyl acetate/n-hexane gradient @ 80 mL/min) and the collected fraction was concentrated under reduced pressure to give the product. Compound 5_4 (1.90 g, 1.97 mmol, 97.8% yield) was obtained as colorless oil and confirmed by SFC.

Intermediate 5_5. A 40 mL vial with a magnetic stir bar was charged with DCM (5 mL), followed by addition of 5_4 (500 mg, 519 μmol, 1.00 eq) and TFA (1.54 g, 13.5 mmol, 1 mL, 25.9 eq) at 25 °C (external temperature). The mixture was stirred at 25 °C (external temperature) for 2 h. LCMS indicated 5_4 was fully consumed, and a peak with desired mass value was observed at RT = 0.455 min. The crude reaction mixture was concentrated under reduced pressure directly to give the crude product. This crude product was used for the next step without purification. Compound 5_5 (440 mg, 478 μmol, 92.0% yield, TFA salt) was obtained as a pale-yellow solid.

Compound p6. A 1000 mL 3-neck round bottom flask with a magnetic stir bar was charged with DCM (400 mL), followed by the addition of DIEA (124 mg, 956 μmol, 167 μL, 2.00 eq) and HATU (236 mg, 622 μmol, 1.30 eq) at 25 °C (external temperature). Then 5_5 (440 mg, 478 μmol, 1.00 eq, TFA) in DCM (40 mL) was added to the mixture. The reaction mixture was allowed to stir vigorously at 25 °C (internal temperature) for 2 h. LCMS indicated 5_5 was fully consumed, and a peak with desired mass value was observed at RT = 1.435 min. The reaction mixture was concentrated under reduced pressure to give a residue. The residue was purified by prep-HPLC (column: Waters Xbridge BEH C18 100*25mm*10um; mobile phase: [H_2O (10 mM NH_4HCO_3)-ACN]; gradient: 40%–80% B over 8.0 min). The collected fraction underwent lyophilization to give the desired product. Compound **p6** (11.9 mg, 14.7 μmol, 3.08% yield, 97.4% purity) was obtained as a white solid and confirmed by ^{1}H NMR, LCMS, and SFC.

S8 LC-MS of Prospective Compounds

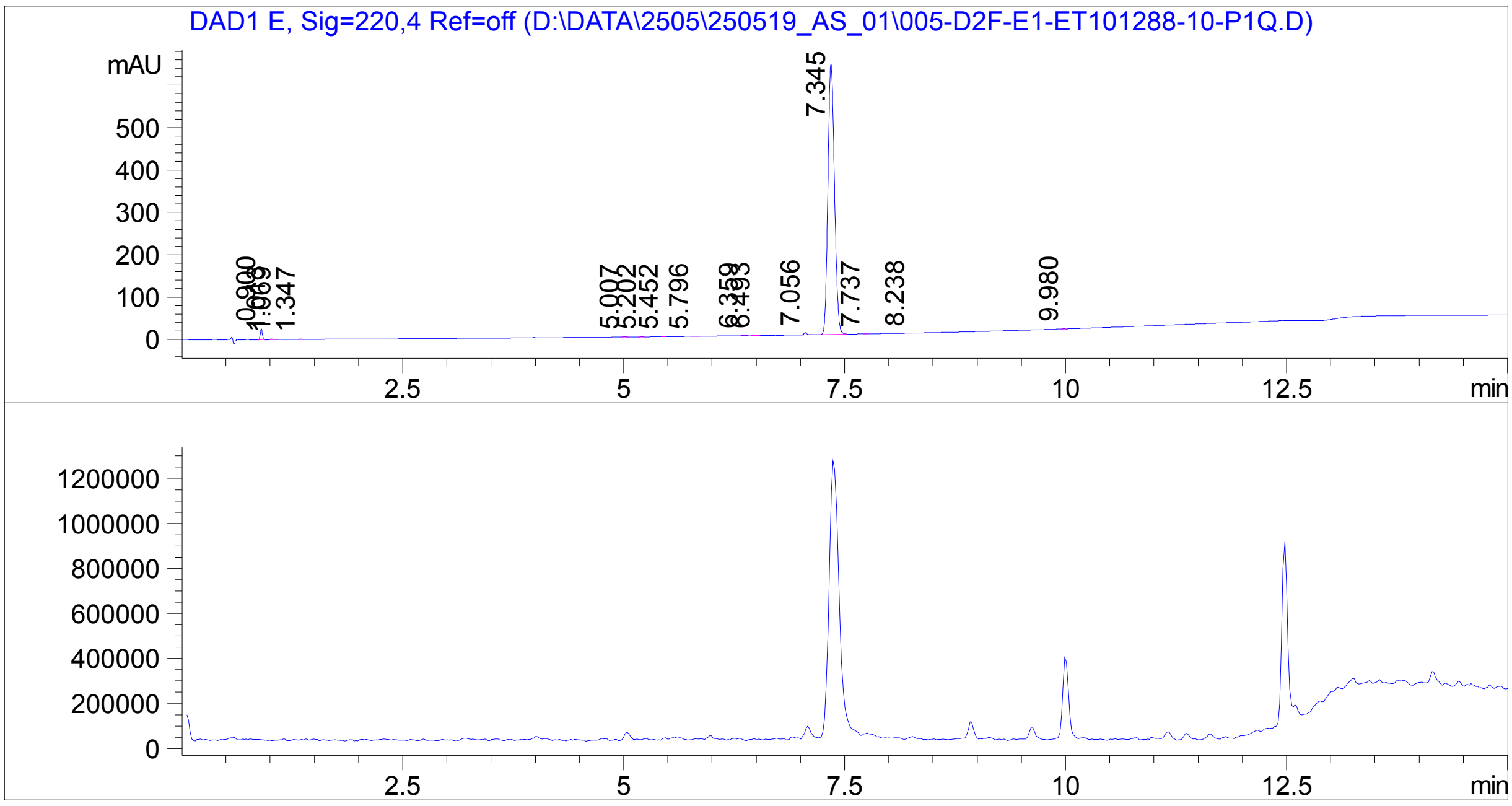

Figure S53: Chromatogram for compound **p0**.

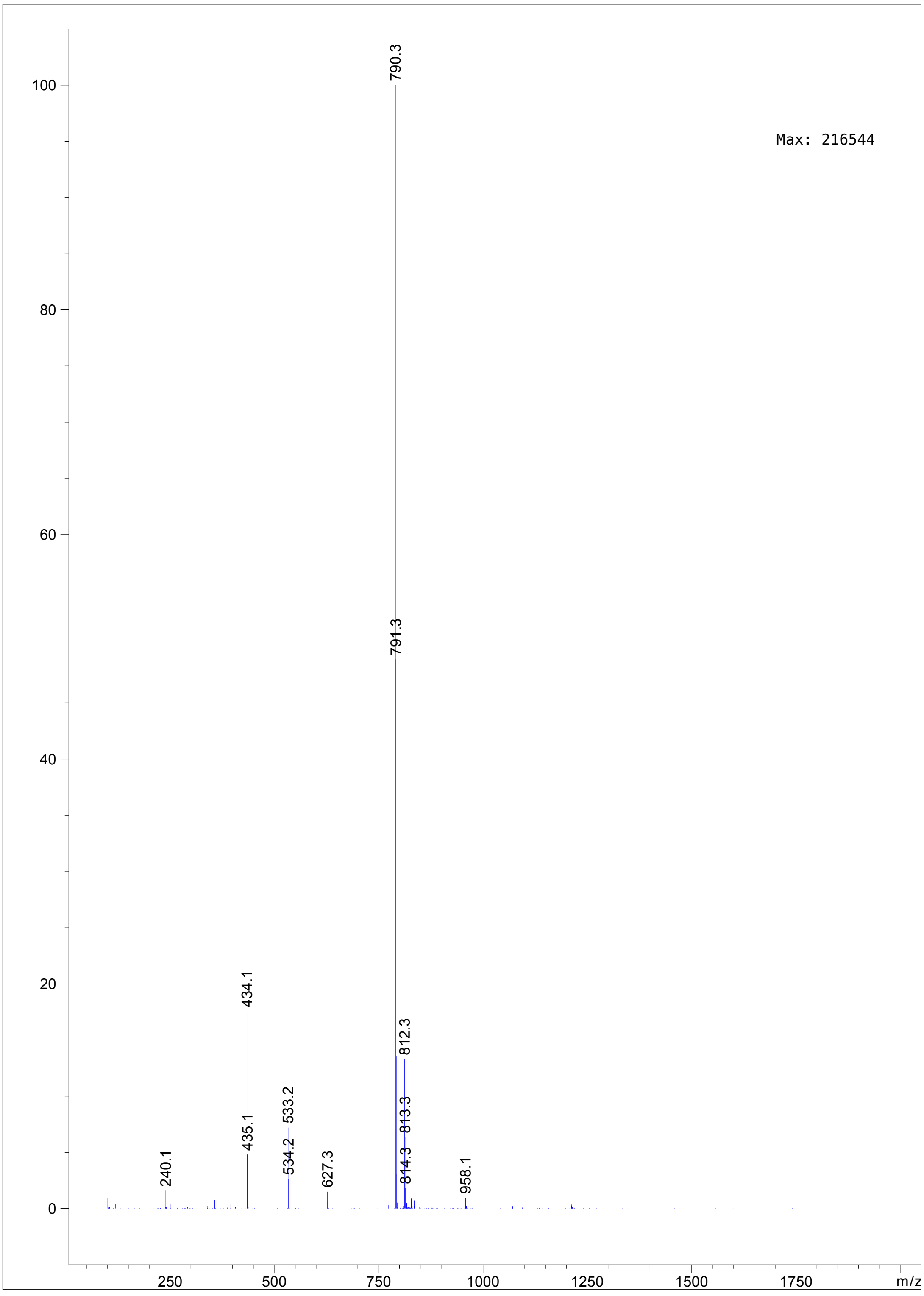

Figure S54: MS spectra for compound **p0**.

The compound p0 was observed by LC–MS at $t_R = 7.345$ min with $m/z = 790.3$, in agreement with the expected protonated molecular ion $[\mathrm{M}+\mathrm{H}]^+$ ($C_{43}H_{56}N_3O_{11}^+$, calculated $m/z = 790.39$).

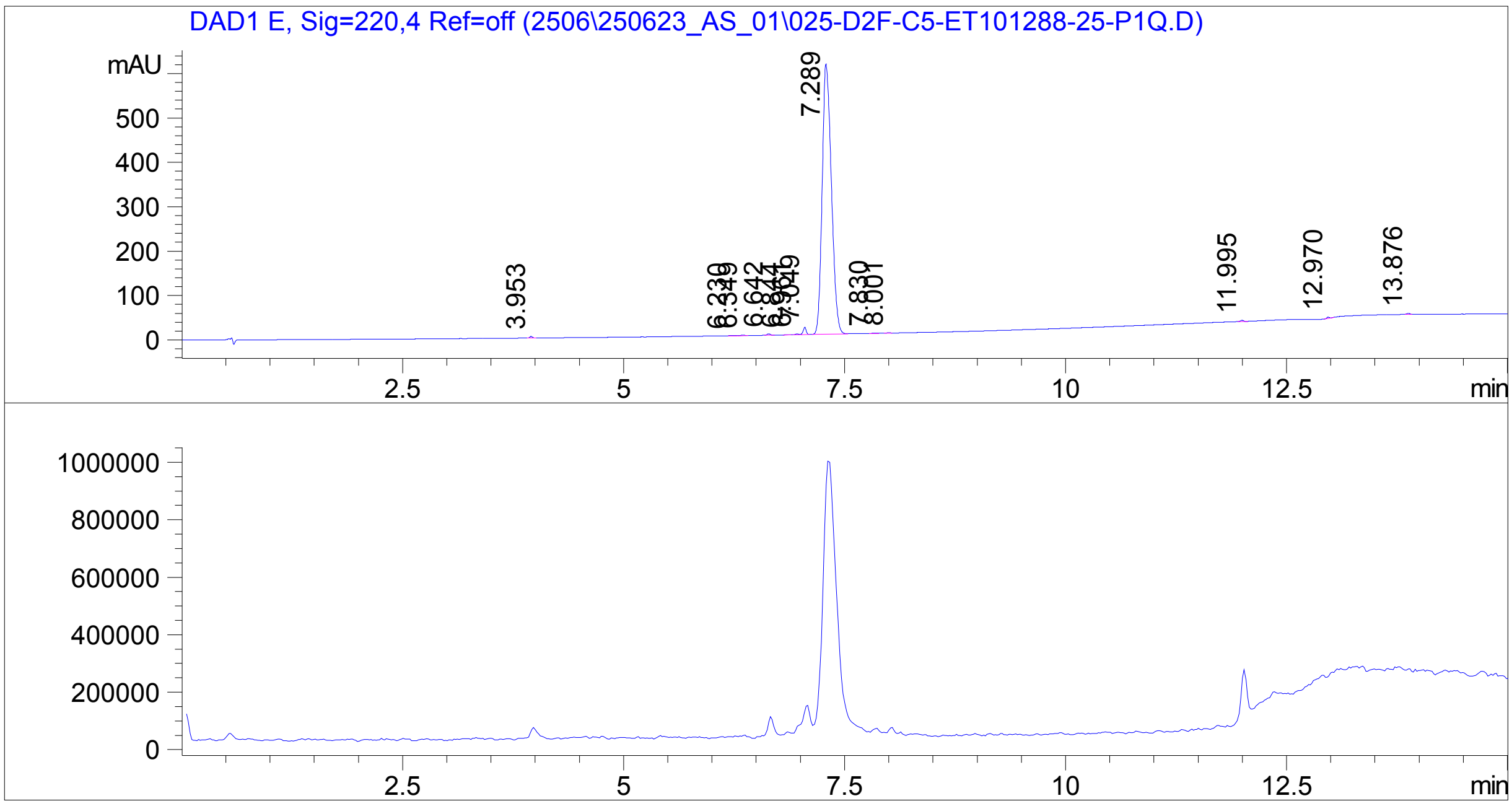

Figure S55: Chromatogram for compound **p2**.

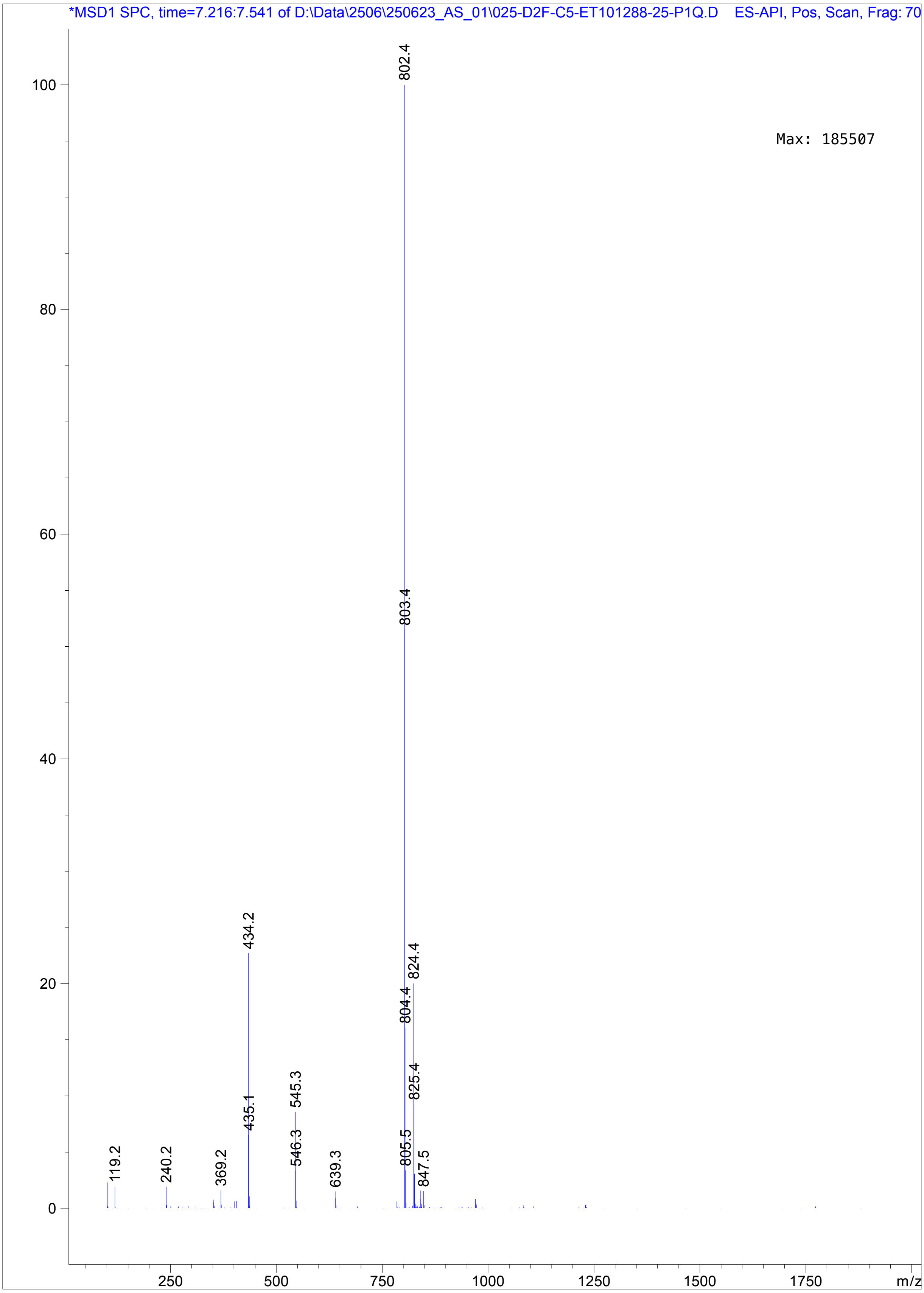

Figure S56: MS spectra for compound **p2**.

The compound p2 was observed by LC–MS at $t_R = 7.289$ min with $m/z = 802.4$, in agreement with the expected protonated molecular ion $[\mathrm{M+H}]^+$ ($C_{44}H_{56}N_3O_{11}^+$, calculated $m/z = 802.39$).

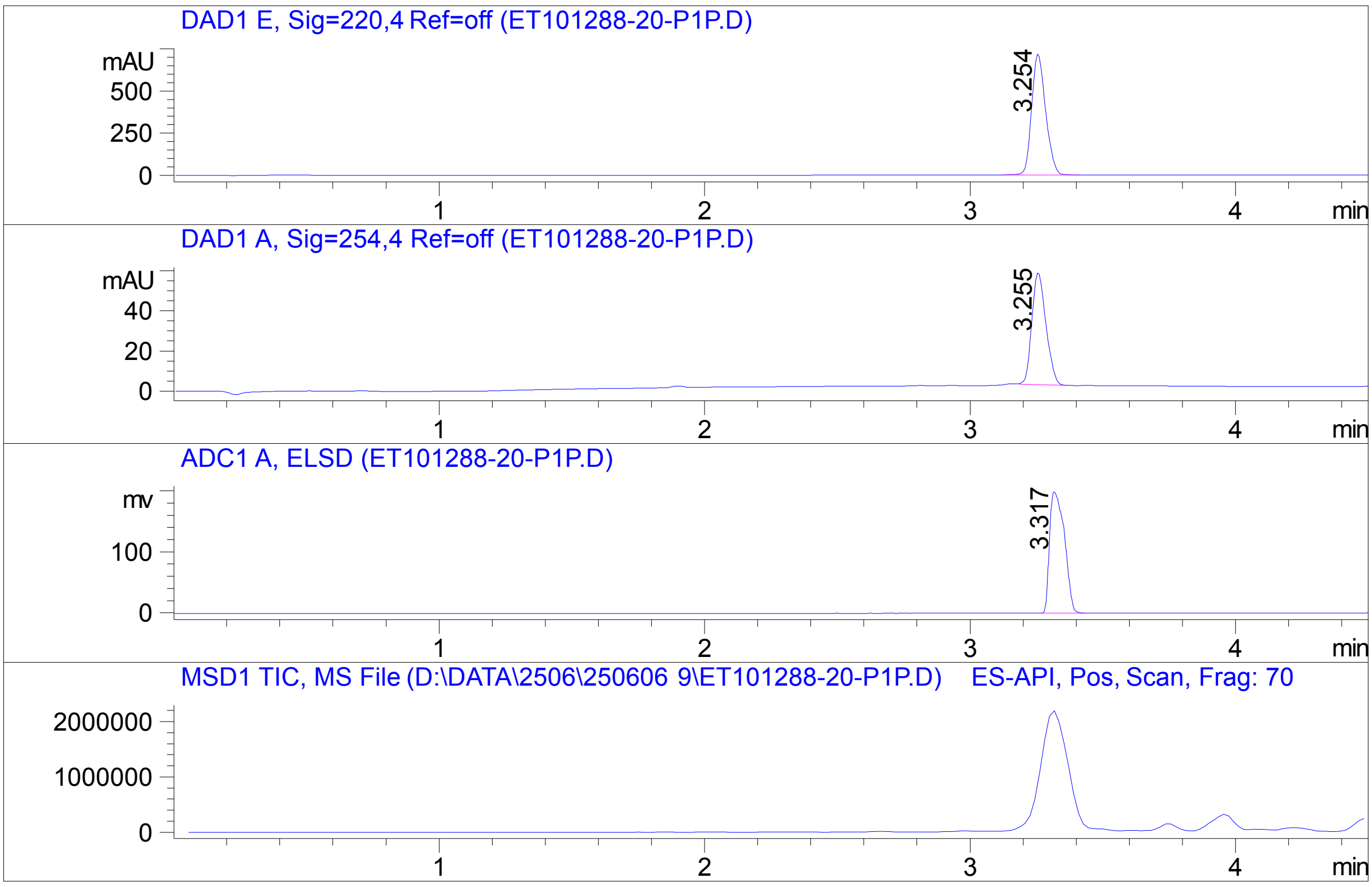

Figure S57: Chromatogram for compound **p3**.

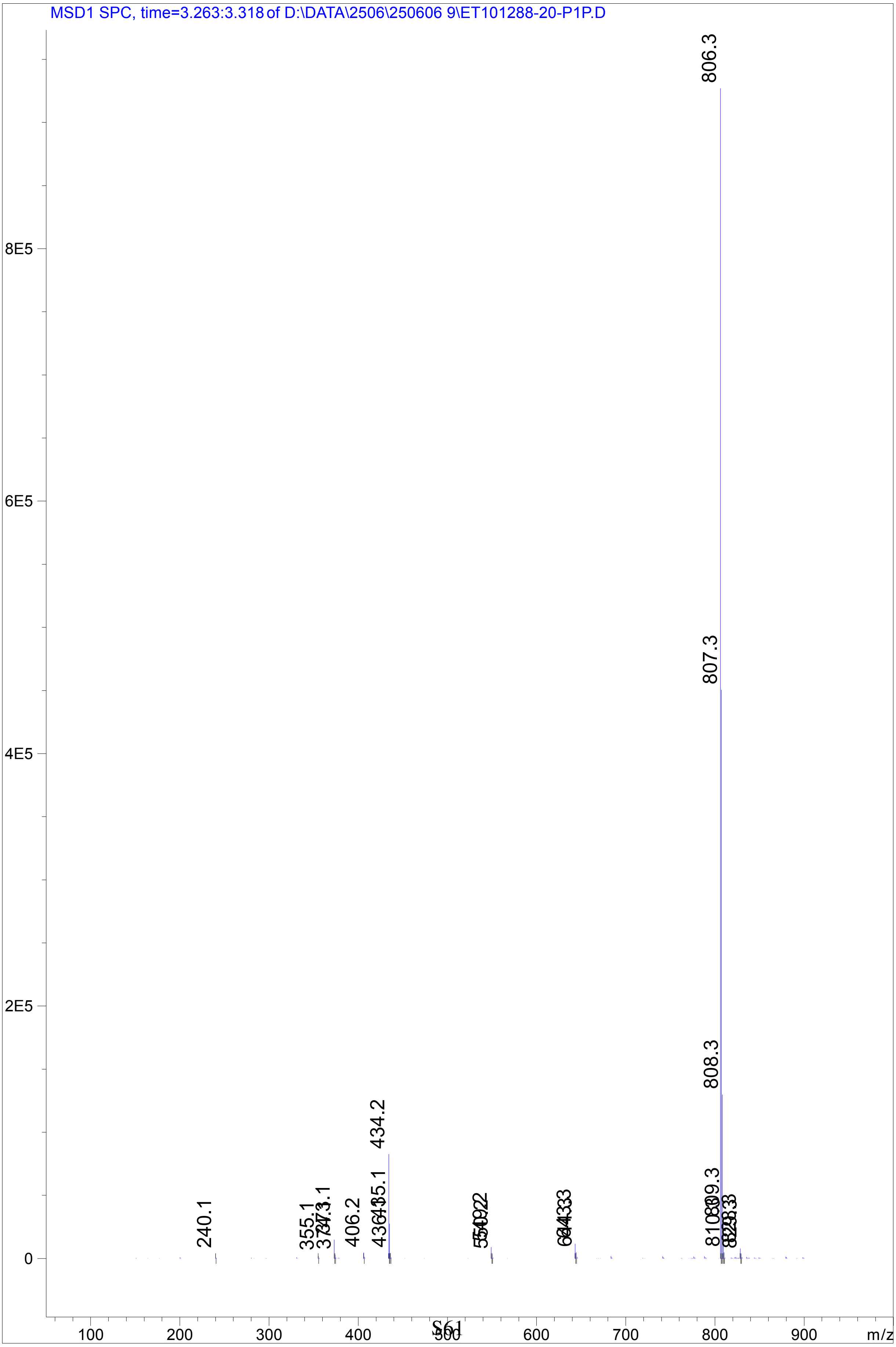

Figure S58: MS spectra for compound **p3**.

The compound p3 was observed by LC–MS at $t_R = 3.254$ min with $m/z = 806.3$, in agreement with the expected protonated molecular ion $[\mathrm{M}+\mathrm{H}]^+$ ($C_{43}H_{56}N_3O_{12}^+$, calculated $m/z = 806.39$).

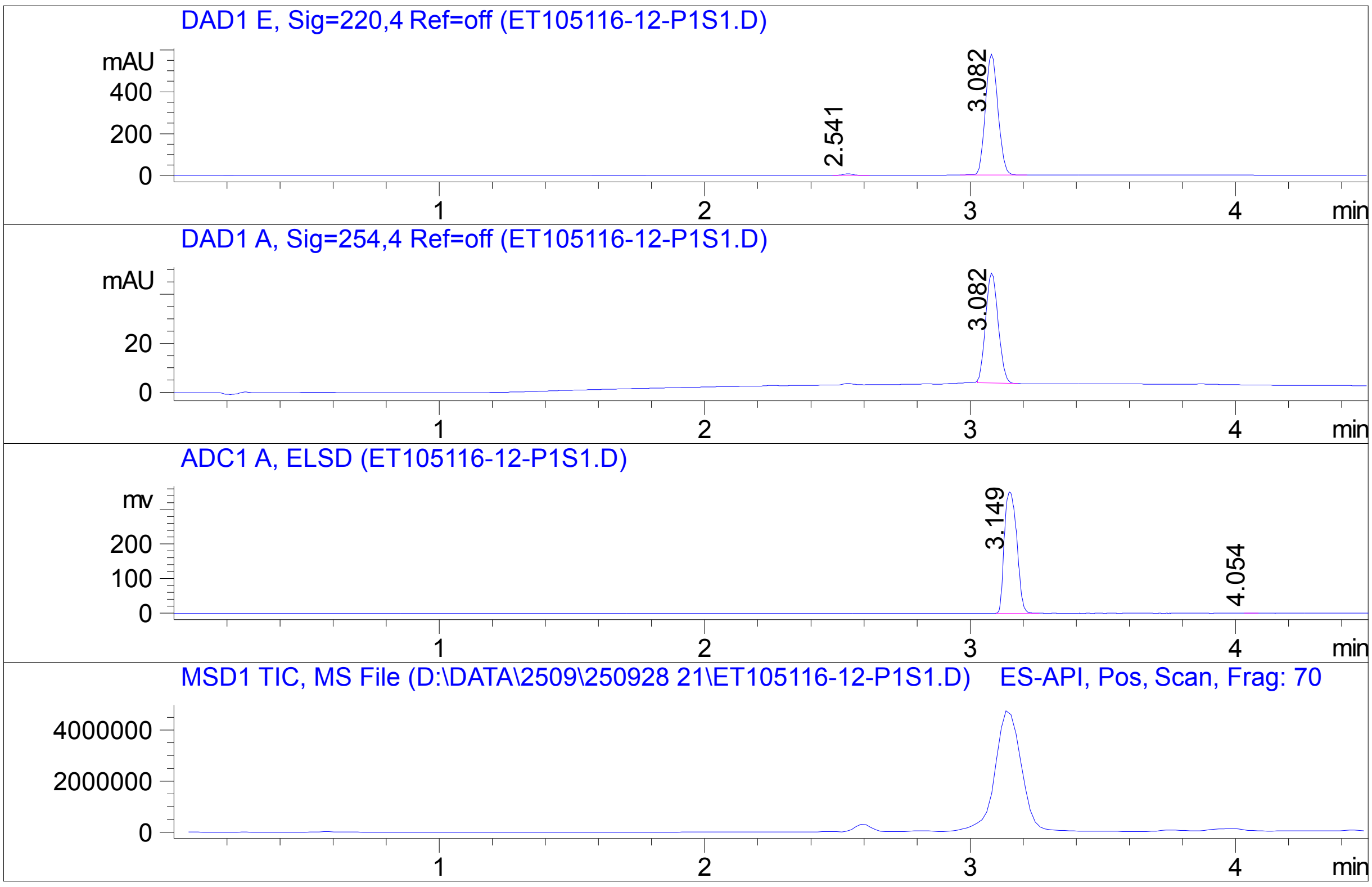

Figure S59: Chromatogram for compound **p4**.

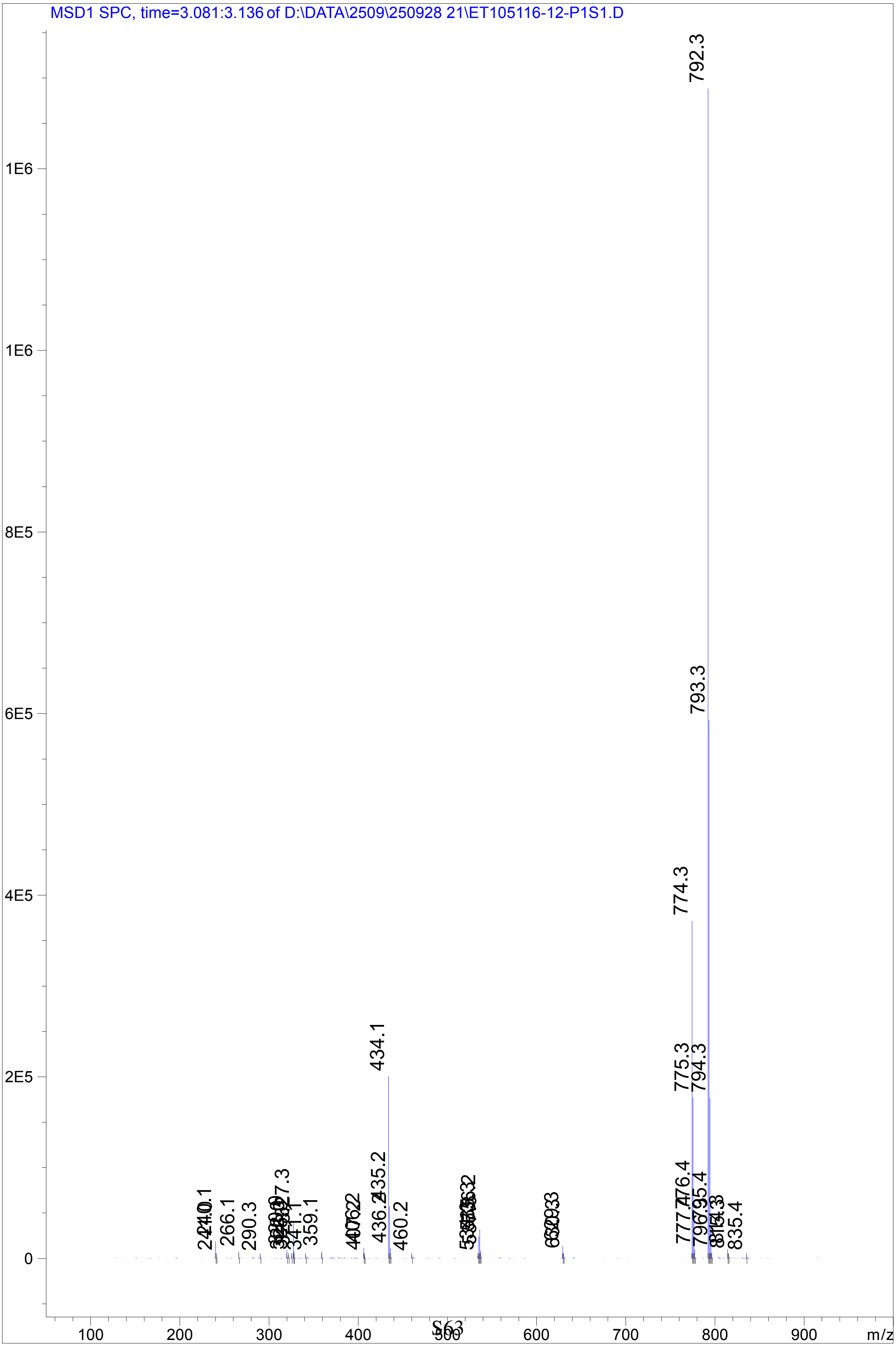

Figure S60: MS spectra for compound **p4**.

The compound p4 was observed by LC–MS at $t_R = 3.082$ min with $m/z = 792.3$, in agreement with the expected protonated molecular ion $[\mathrm{M+H}]^+$ ($C_{42}H_{54}N_3O_{12}^+$, calculated $m/z = 792.37$).

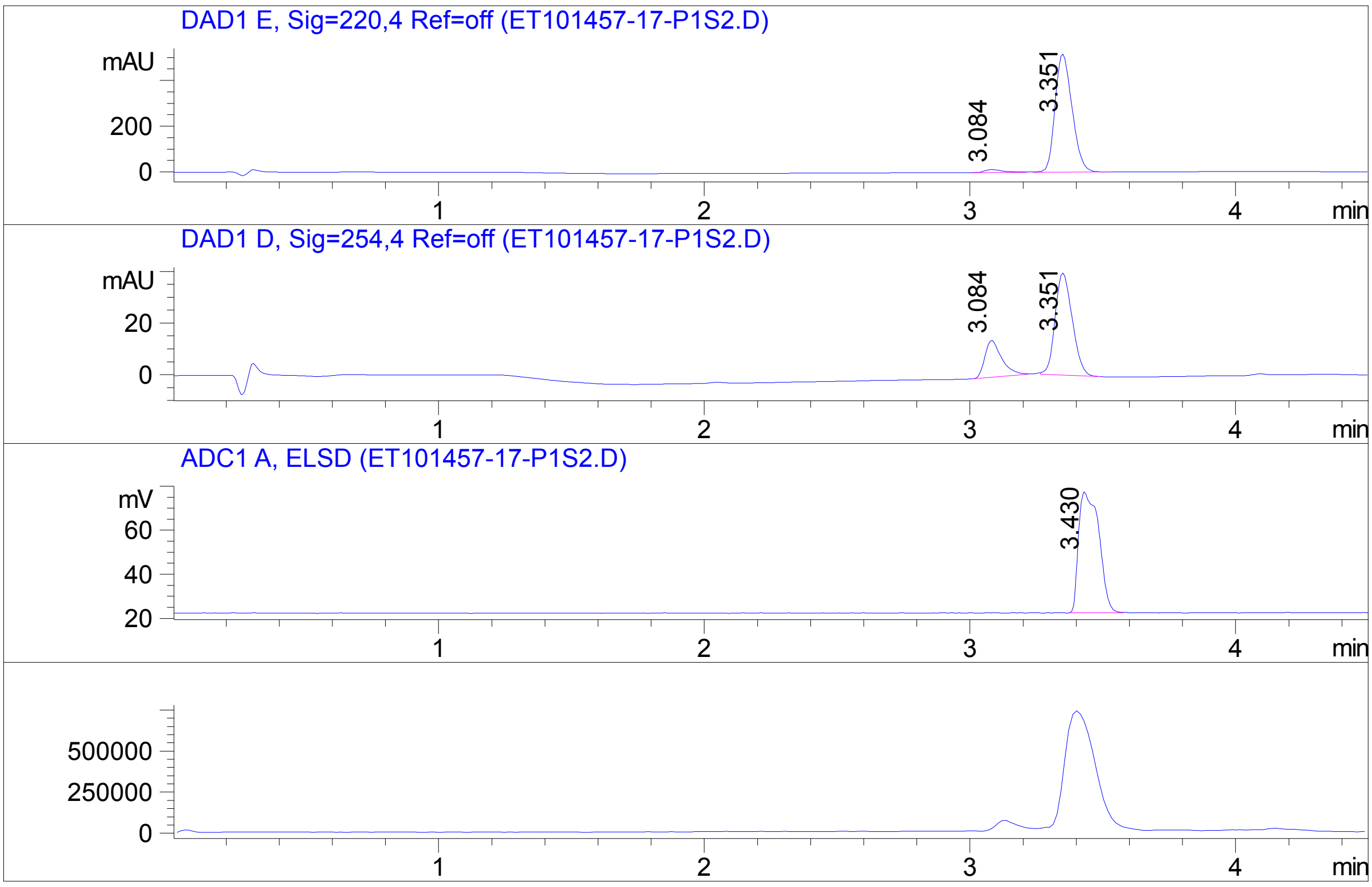

Figure S61: Chromatogram for compound **p6**.

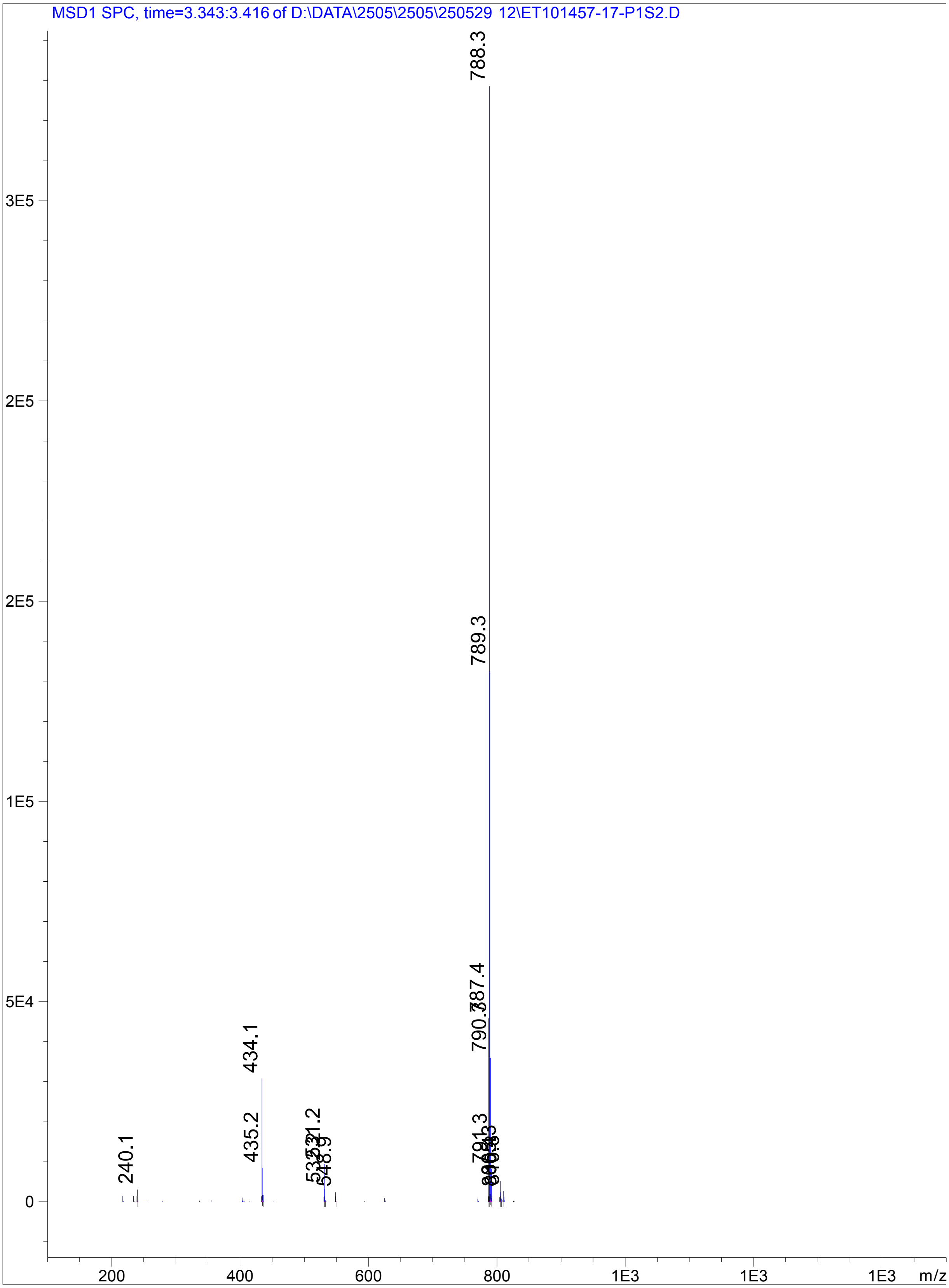

Figure S62: MS spectra for compound **p6**.

The compound p6 was observed by LC–MS at $t_R = 3.351$ min with $m/z = 788.3$, in agreement with the expected protonated molecular ion $[\mathrm{M+H}]^+$ ($C_{43}H_{54}N_3O_{11}^+$, calculated $m/z = 788.38$).

S9 ^{1}H-NMR Spectra of Prospective Compounds

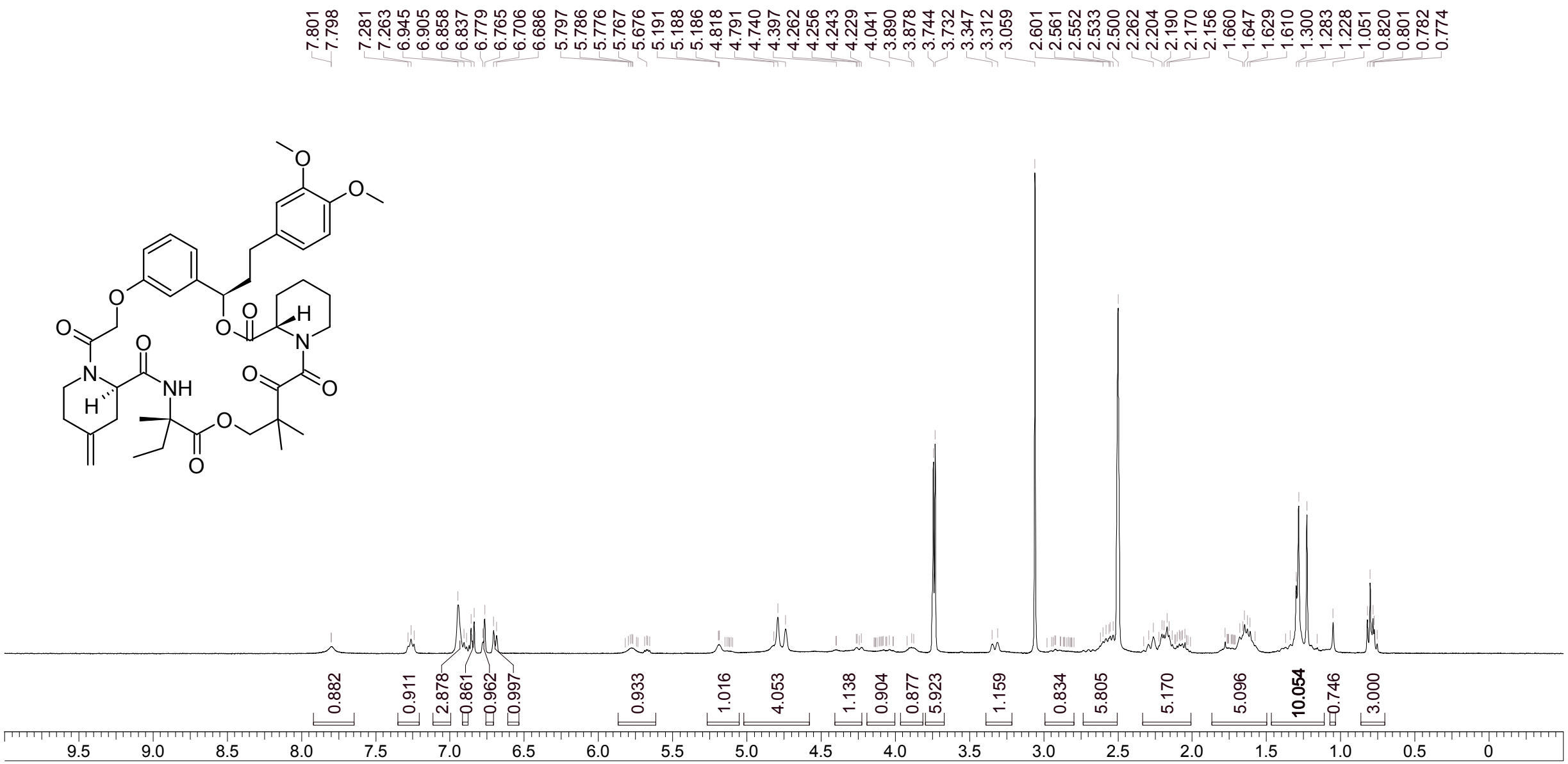

Figure S63: ^{1}H-NMR spectrum for compound **p0**.

p0. ^{1}H NMR (400 MHz, DMSO-d_6, 353 K) δ 7.81 (br d, J = 1.1 Hz, 1H), 7.27 (br t, J = 7.6 Hz, 1H), 7.01 - 6.88 (m, 3H), 6.86 (d, J = 8.1 Hz, 1H), 6.81 - 6.76 (m, 1H), 6.70 (br d, J = 8.1 Hz, 1H), 5.93 - 5.53 (m, 1H), 5.31 - 5.06 (m, 1H), 4.99 - 4.59 (m, 4H), 4.46 - 4.24 (m, 1H), 4.16 - 3.99 (m, 1H), 3.96 - 3.82 (m, 1H), 3.75 (d, J = 4.9 Hz, 6H), 3.34 (br d, J = 14.1 Hz, 1H), 2.97 - 2.78 (m, 1H), 2.54 (br s, 6H), 2.35 - 1.98 (m, 5H), 1.85 - 1.50 (m, 5H), 1.48 - 1.14 (m, 10H), 1.06 (s, 1H), 0.91 - 0.67 (m, 3H).

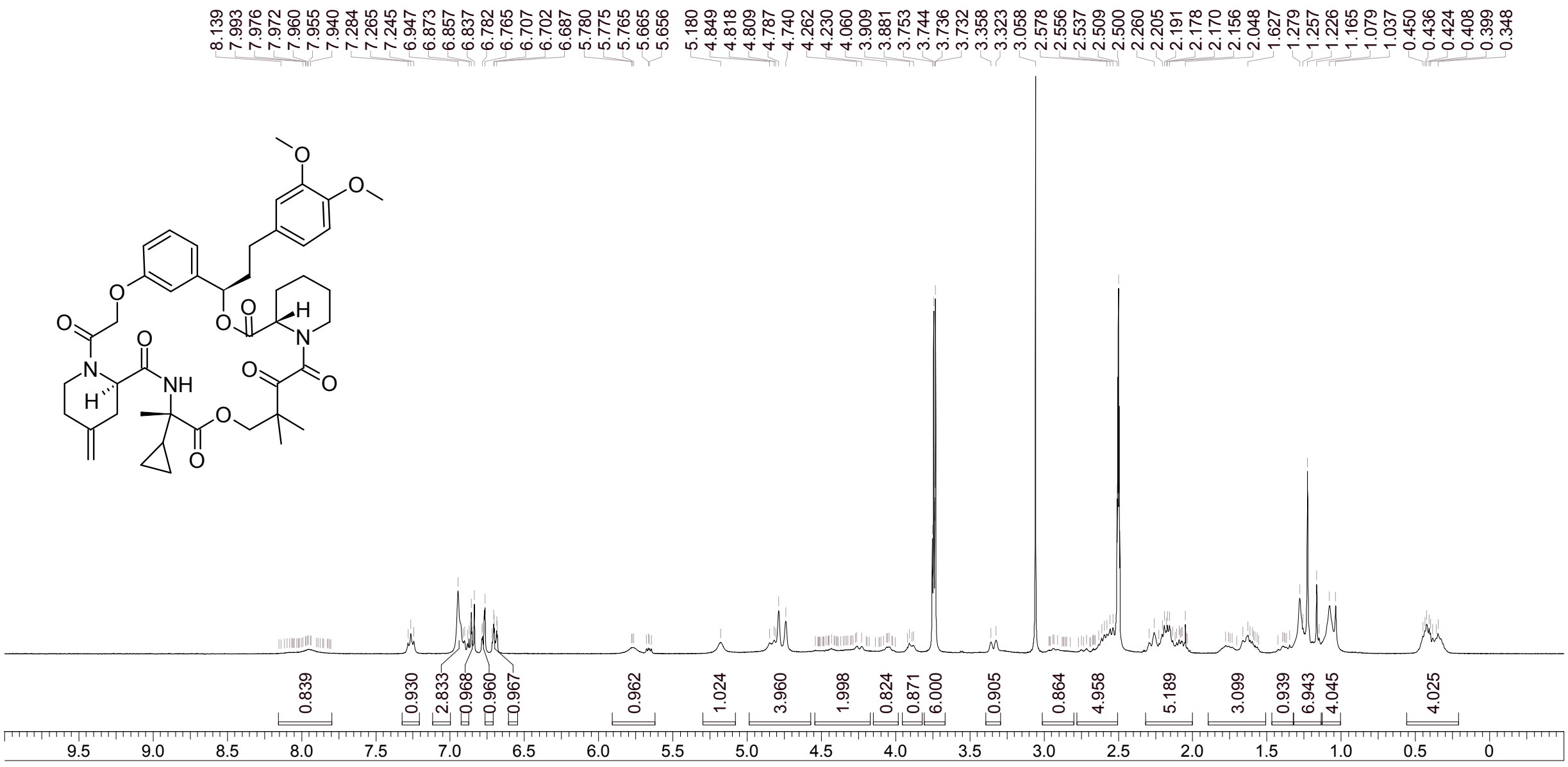

Figure S64: ^{1}H-NMR spectrum for compound **p2**.

p2. ^{1}H NMR (400 MHz, DMSO-d_6, 353 K) δ 8.16–7.80 (m, 1H), 7.26 (br t, J = 7.7 Hz, 1H), 7.00–6.88 (m, 3H), 6.88–6.83 (m, 1H), 6.80–6.75 (m, 1H), 6.73–6.67 (m, 1H), 5.91–5.62 (m, 1H), 5.18 (br s, 1H), 4.99–4.57 (m, 4H), 4.55–4.17 (m, 2H), 4.15–3.98 (m, 1H), 3.95–3.82 (m, 1H), 3.81–3.67 (m, 6H), 3.34 (br d, J = 14.1 Hz, 1H), 3.01–2.80 (m, 1H), 2.78–2.51 (m, 5H), 2.32–2.00 (m, 5H), 1.89–1.51 (m, 3H), 1.47–1.32 (m, 1H), 1.32–1.13 (m, 7H), 1.06 (br d, J = 16.6 Hz, 4H), 0.56–0.21 (m, 4H).

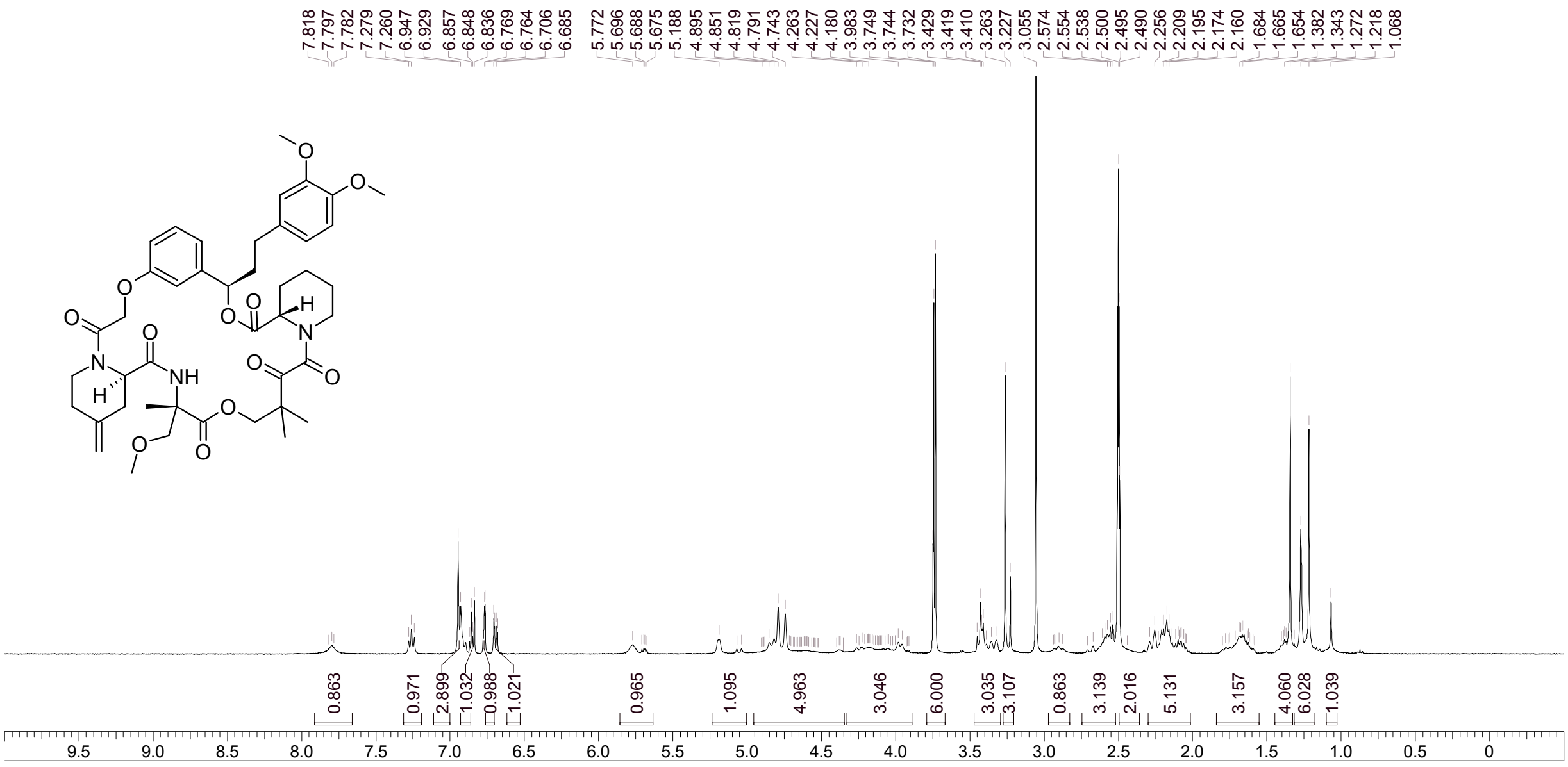

Figure S65: ^{1}H-NMR spectrum for compound **p3**.

p3. ^{1}H NMR (400 MHz, DMSO-d_6, 353 K) δ 7.91–7.66 (m, 1H), 7.26 (t, J = 7.7 Hz, 1H), 6.99–6.89 (m, 3H), 6.88–6.82 (m, 1H), 6.77 (d, J = 2.0 Hz, 1H), 6.74–6.65 (m, 1H), 5.86–5.63 (m, 1H), 5.24–5.00

(m, 1H), 4.95–4.35 (m, 5H), 4.33–3.89 (m, 3H), 3.79–3.67 (m, 6H), 3.47–3.29 (m, 3H), 3.28–3.21 (m, 3H), 2.97–2.83 (m, 1H), 2.74–2.52 (m, 3H), 2.49 (br s, 2H), 2.30–2.02 (m, 5H), 1.84–1.55 (m, 3H), 1.44–1.33 (m, 4H), 1.32–1.18 (m, 6H), 1.07 (s, 1H).

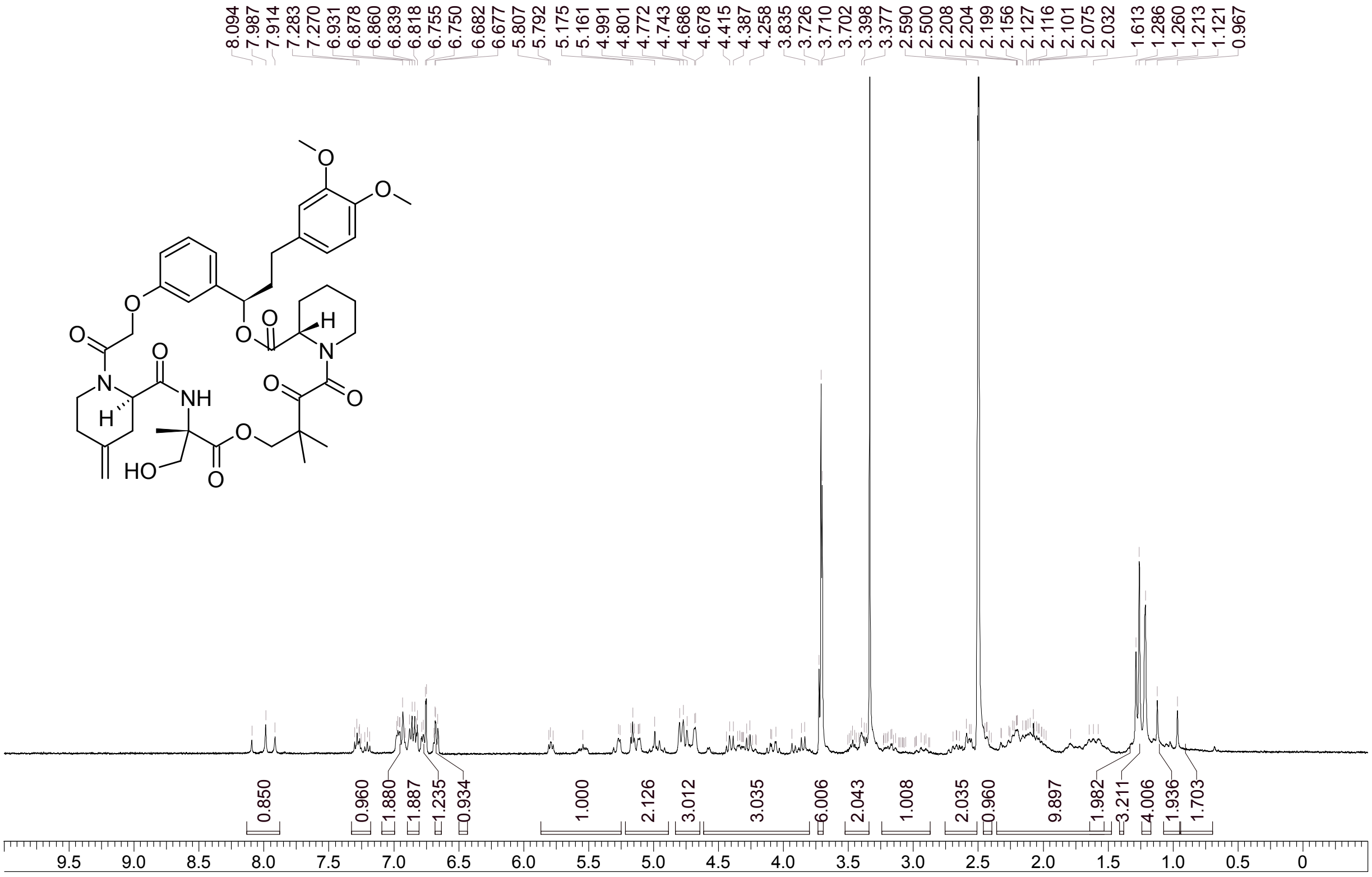

Figure S66: ^{1}H-NMR spectrum for compound **p4**.

p4. ^{1}H NMR (400 MHz, DMSO-d_6, 293.70 K) δ 8.13–7.88 (m, 1H), 7.33–7.18 (m, 1H), 7.00–6.90 (m, 2H), 6.90–6.81 (m, 2H), 6.79–6.74 (m, 1H), 6.71–6.64 (m, 1H), 5.87–5.25 (m, 1H), 5.21–4.89 (m, 2H), 4.83–4.64 (m, 3H), 4.62–3.80 (m, 3H), 3.74–3.69 (m, 6H), 3.53–3.34 (m, 2H), 3.24–2.87 (m, 1H), 2.76–2.51 (m, 2H), 2.46–2.39 (m, 1H), 2.36–1.47 (m, 10H), 1.29 (s, 2H), 1.26 (s, 3H), 1.21 (br s, 4H), 1.12 (s, 2H), 0.97 (s, 2H).

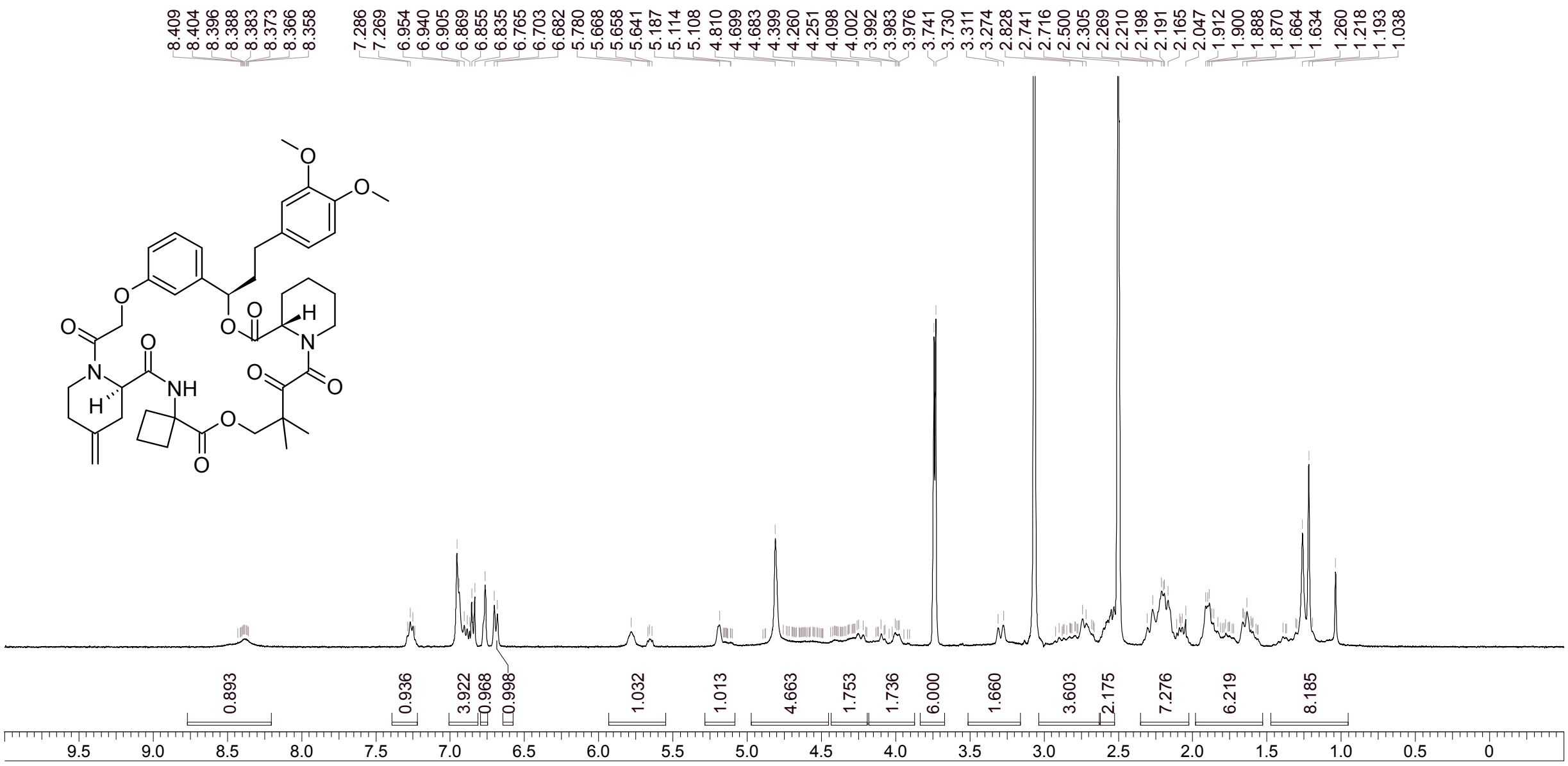

Figure S67: ^{1}H-NMR spectrum for compound **p6**.

p6. ^{1}H NMR (400 MHz, DMSO-d_6, 353 K) δ 8.77–8.21 (m, 1H), 7.39–7.22 (m, 1H), 7.01–6.81 (m, 4H), 6.77 (s, 1H), 6.69 (br d, J = 8.1 Hz, 1H), 5.93–5.55 (m, 1H), 5.28–5.08 (m, 1H), 4.97–4.45 (m, 4H), 4.44–4.19 (m, 2H), 4.18–3.87 (m, 2H), 3.74 (d, J = 4.5 Hz, 6H), 3.51–3.16 (m, 2H), 3.04–2.63 (m, 4H), 2.62–2.53 (m, 2H), 2.35–2.03 (m, 7H), 1.98–1.53 (m, 6H), 1.47–0.95 (m, 8H).

S10 Analytical Characterization of Final Compounds

Compound	Isolated amount	Yield	Physical form	Reported purity	Chiral chromatography	LC–MS
**p0**	130 mg	59.33%	white solid	97.70%	$t_R = 2.005$ min	t_R = 7.345 min; $[\mathrm{M+H}]^+$ 790.3
**p2**	10.42 mg	4.24%	white solid	97.89%	$t_R = 2.435$ min	t_R = 7.289 min; $[\mathrm{M+H}]^+$ 802.4
**p3**	11.07 mg	12.80%	white solid	100%	$t_R = 2.514$ min	t_R = 3.254 min; $[\mathrm{M+H}]^+$ 806.3
**p4**	12.1 mg	1.22%	white solid	99.00%	$t_R = 2.196$ min	t_R = 3.082 min; $[\mathrm{M+H}]^+$ 792.3
**p6**	11.90 mg	3.08%	white solid	97.40%	$t_R = 1.259$ min (2.13%), 1.662 min (97.87%)	t_R = 3.351 min; $[\mathrm{M+H}]^+$ 788.3

Table S1: Summary of the analytical characterization of the prospective compounds. Chiral chromatographic retention times and, where applicable, relative peak areas are reported.

References

[1] Salcius, M. et al. Identification and Characterization of Ternary Complexes Consisting of FKBP12, MAPRE1 and Macrocyclic Molecular Glues. *RSC Chem. Biology* **2025**, *6*, 788–799.

[2] Schnatwinkel, J.; Stein, R. R.; Salcius, M.; Wong, J. L.; Chen, S.-Y.; Fouché, M.; Roth, H.-J. Experimental Conditions to Retrieve Intrinsic Cooperativity α Directly From Single Binding Assay Data

Exemplified by the Ternary Complex Formation of FKBP12, MAPRE1 and Macrocyclic Molecular Glues. *Int. J. Mol. Sci.* **2025**, *26*, 2936.
